# The response of wheat yield, water productivity, and partial factor productivity of nitrogen to straw return and biochar application: a global meta-analysis

**DOI:** 10.3389/fpls.2026.1923733

**Published:** 2026-09-30

**Authors:** Zhihao Yang, Weiwei Chen, Mingan Lu, Penghui Ma, Xing Yu, Ling Li, Shufang Wu, Yajin Hu

**Affiliations:** 1 College of Resources and Environment, Henan Agricultural University, Zhengzhou, China; 2 Yellow River Institute of Hydraulic Research, Yellow River Conservancy Commission, Zhengzhou, China; 3 State Key Laboratory of Soil Erosion and Dryland Farming on the Loess Plateau, Northwest A&F University, Yangling, Shanxi, China; 4 Postdoctoral station of Crop science, Henan Agricultural University, Zhengzhou, Henan, China

**Keywords:** circular economy, grain, meta-analysis, organic materials, production, use of stabilized compost

## Abstract

Straw return (SR) and biochar application (BA) are widely used organic amendments with potential to improve wheat productivity and resource-use efficiency. However, their responses across environmental and field-management conditions remain poorly synthesized. We conducted a global multilevel meta-analysis of 2,242 yield, 1,239 water productivity (WP), and 1,988 partial factor productivity of nitrogen (PFPN) observation pairs from 385 publications. SR increased wheat yield, WP, and PFPN by 8.31%, 8.76%, and 8.28%, respectively, whereas BA increased them by 10.34%, 11.48%, and 9.96%. Subgroup and Random Forest analyses showed that the magnitude of these benefits was mainly associated with soil properties and field management. Lower nitrogen inputs favored the yield and PFPN responses to SR, whereas increasing biochar application rate strengthened yield and PFPN responses to BA. Water supply was an important predictor of WP under both amendments. These results demonstrate that SR and BA can simultaneously enhance wheat productivity and water and nitrogen use. Their agronomic benefits can be further improved by matching amendment practices with local soil fertility, water availability, and nutrient-management conditions.

## Introduction

Wheat is one of the three major staple crops in the world, accounting for 26% of global grain production (FAO, 2013), and more than 40% of the world’s population consumes wheat as a staple food. China, as the dominant global producer of wheat, plays a critical role in maintaining stability in international grain markets through sustained improvements in wheat production systems (He et al., 2001; Islam et al., 2022). Furthermore, the World Resources Institute (WRI) (2019) reported that the gap between food supply and demand will widen by 2050. While global agricultural output has doubled since the late 20th century, intensive farming systems significantly contribute to environmental degradation and soil health deterioration (Liu et al., 2024). Therefore, improving crop productivity per unit area with limited arable land is key to food security at regional, national, and global scales (Crystal-Ornelas et al., 2021). The 2030 Sustainable Development Goals related to food and agriculture are still far from being achieved (FAO, 2023). Optimizing straw return and biochar application methods will be needed to enhance wheat yield, water productivity (WP), and partial factor productivity of nitrogen (PFPN). In turn, this will require the adoption of appropriate institutions, capacity building, and incentivized policies.

Straw residue, as an organic fertilizer, is abundant in nutrients, including nitrogen, phosphorus, and potassium, making it one of the most significant renewable resources in the world (Singh and Sidhu, 2014; Jin et al., 2020). Straw return (SR) is one of the most convenient and efficient ways to utilize straw residue in agricultural production (Jin et al., 2017). More importantly, direct burning of straw will release pollutants and damage the ecological environment (Liu et al., 2021). SR can positively affect the structure of bacterial communities (Chen et al., 2017), reduce soil bulk density, decrease soil evaporation and erosion (Malhi et al., 2011), improve soil fertility (Blanco-Canqui and Lal, 2017), and enhance soil enzyme activity (Qin et al., 2013). The effects of SR on crops under different field tillage methods also showed significant differences (Chen et al., 2022). Akhtar et al. (2023) reported that SR achieved higher crop yields, WP, and PFPN in a maize–wheat crop rotation system. Nitrogen fertilizers and inappropriate straw return amounts (SRA) can trigger nitrogen competition between crops and microorganisms, thereby decreasing crop yield and PFPN (Chen et al., 2014). The influence of SR on crops should be evaluated by taking into account factors such as experimental location, climatic conditions, soil characteristics, and field management (Karan and Hamelin, 2021). Independent experiments have demonstrated the effects of SR with nitrogen fertilizer, tillage methods, and irrigation on wheat yield, WP, PFPN, and greenhouse gas emissions (Thomsen and Christensen, 2004; Yao et al., 2009; Wang and Shangguan, 2015; Chen et al., 2017; Guo et al., 2024). These independent studies cannot provide a comprehensive understanding on a larger scale. Thus, meta-analysis can provide an accurate overall analysis of the results by summarizing independent experimental data under the same theme; it also minimizes subjective judgment and ensures the consistency and validity of the analysis (Hedges et al., 1999). The majority of meta-analyses of SR have analyzed individual topics, such as yield (Zhang et al., 2024; Moura Chagas et al., 2022; Cheng et al., 2025); soil organic carbon (SOC) (Wang et al., 2021; Xin et al., 2024; Berhane et al., 2020); greenhouse gas emissions (Liu et al., 2023b; Hu et al., 2025); soil microbial diversity (Zhang et al., 2024a; Geng et al., 2024); and nitrogen fertilizers and farming methods (Yan et al., 2024; Lin et al., 2024; Islam et al., 2023; Huang et al., 2023). However, attention to wheat at the global scale is limited, and an overall analysis covering the countrywide scale is essential for evaluating the response of straw return to wheat under different natural and field management conditions.

Biochar is charcoal made from agricultural materials pyrolyzed under low-oxygen and high temperature conditions. It has a high surface area and low density (Smith, 2016). These features can create an environment that stimulates soil microorganisms and crop root growth, improve soil enzyme activity and soil nutrient availability (Jiao et al., 2011; Dai et al., 2017), and improve crop yield and PFPN (Jeffery et al., 2011). Moreover, as a kind of potential biochar soil conditioner, BA can enhance soil physical characteristics, soil water retention, and WP (Mak-Mensah et al., 2022), reduce soil bulk weight (Burrell et al., 2016), improve soil carbon sequestration capacity (Biederman and Harpole, 2013; Chen et al., 2018), and reduce fertilizer use to save resources (Guo et al., 2024). The efficacy of BA is influenced by its intrinsic characteristics and the environment in which it is applied (Gu et al., 2020). For example, Tang et al. (2023) reported that biochar improved deteriorated soil structure in saline-alkali soils and increased nitrogen absorption and utilization. Guo et al. (2024) showed that a high biochar application rate (BAR) can enhance water-holding capacity in coarse soils. Chen et al. (2018) showed that biochar produced at high pyrolysis temperatures can improve field capacity. However, long-term field studies are required to determine the extent of biochar’s effects (Blanco-Canqui, 2017). Meta-analysis examines the impact of variables on a specific topic and can inform modeling decisions (Smith and Kaoru, 1990). For instance, Han et al. (2023) found that soil and biochar properties are critical to the effect of BA. Liu et al. (2013) examined the relationships among BA, soil pH, and the biochar carbon-to-nitrogen (C/N) ratio and their effects on crop productivity. Moura et al. (2022) showed that BA improved C sequestration in the soil. Furthermore, Hu et al. (2025) also found that biochar plus nitrogen fertilizer significantly improved crop yield more than biochar alone. Moreover, Liu et al. (2022) and Wu et al. (2022) indicated that different biochar usage led to different improvements in water and nitrogen use efficiencies. Different emphasis also leads to different conclusions. Although meta-analysis can identify more ideal ranges of variables, subgroup analyses may identify field-management operations that lack practical significance (Liu et al., 2023a). A random forest model can further identify key variables that affect crop growth (Dang et al., 2022; Liu et al., 2023b). In this study, a systematic survey was conducted based on data for wheat yield, WP, and PFPN under SR and BA. The meta-analysis aims to: (1) assess the overall influence of SR and BA on wheat yield, WP, and PFPN in a global context; (2) examine the effects of biochar on wheat yield, WP, and PFPN under various climatic conditions, soil characteristics, and agricultural field practices; and (3) identify the principal elements affecting the efficacy of SR and BA.

## Materials and methods

### Data collection

In this study, we collected literature data from 2000 to 2025 to investigate the effects of SR and BA on wheat yield, WP, and PFPN. Data were collected from Web of Science (http://apps.webofknowledge.com/), Google Scholar (https://scholar.google.com/), and China National Knowledge Infrastructure (https://www.cnki.net/); keywords including (“wheat”) AND (“straw return” OR “straw mulching” OR “biochar”) AND (“yield” OR “production” OR “water productivity (WP)” OR “partial factor productivity of nitrogen (PFPN)”) were employed to search for peer-reviewed journal articles. The literature selected for inclusion in the study had to meet the following criteria: (1) Field experiments with specific sites and years clearly identified; (2) Each independent study included a control (no straw or no biochar: CK) and treatments (straw return or biochar application); (3) The study used a replicated plot design with at least three replicates per treatment; pot experiments, rainproof shelters, and model simulations were excluded. Data on wheat yield, WP, or PFPN under SR or BA had to be available in the reported results. The primary metrics for comparison were wheat yield, WP, and PFPN. The ratios of crop yield to evaporation and to total nitrogen fertilizer input were used to impute missing WP and PFPN values to improve data coverage. (4) The mean, standard deviation (or standard error), and sample size of the variables were either readily accessible or could be derived from the data. (5) When WP and PFPN were directly reported in the original studies, the reported values were extracted; otherwise, when the primary data required for their calculation were available, these indicators were derived from the reported data according to the standard definitions adopted in previous meta-analyses (Qin et al., 2015; Li et al., 2020; Adil et al., 2022). (6) When a publication reported multiple observations from different organic material treatments, multifactorial experiments, or multiple years or growing seasons, each eligible treatment–control comparison was extracted separately and treated as an individual study unit in the dataset. Secondary screening was conducted following evaluation of the titles and abstracts to exclude studies that did not meet the selection criteria. A total of 385 articles were selected, including 247 publications on SR for wheat (1,388 pairs of yield, 843 pairs of WP, and 1,225 pairs of PFPN) and 138 publications on BA (854 pairs of yield, 396 pairs of WP, and 752 pairs of PFPN). The locations of the wheat experimental sites in the meta-analysis are shown in **Figure 1**.

**Figure 1:**
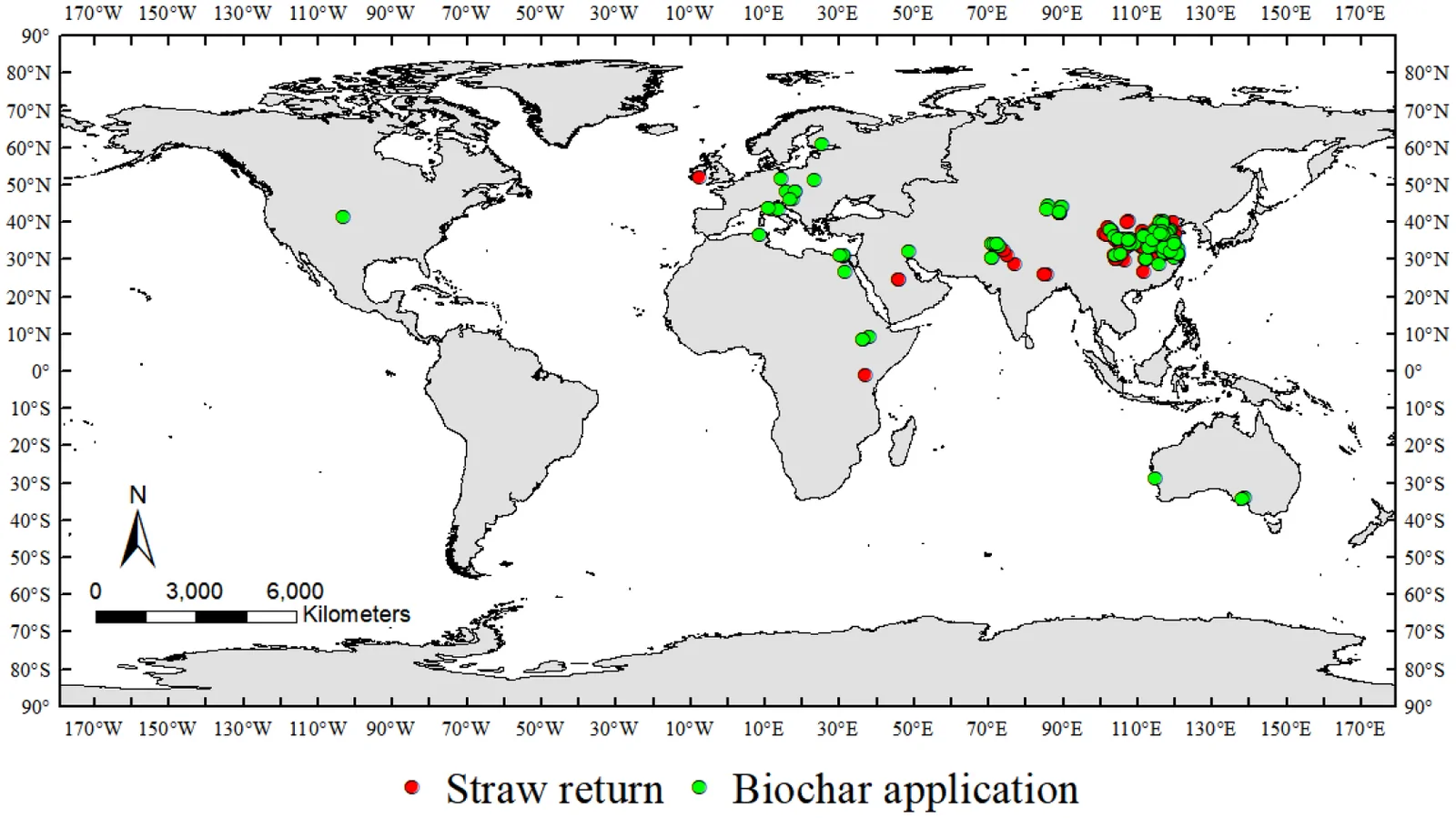
Global spatial distribution of experimental sites used in the meta-analysis.

### Data classification

We used the following criteria to determine the effects of SR and BA on improvements in wheat yield, WP, and PFPN. (1) Climatic conditions included growing season precipitation (GSP) (< 150 mm, 150–300 mm, 300–450 mm, and > 450 mm) and growing season temperature (GST) (< 10 °C, 10–15 °C and > 15 °C). (2) Soil properties included soil texture (coarse, medium, and fine soils). Soil texture was determined according to the United States Department of Agriculture (https://www.usda.gov/) guidelines (USDA, 1999). Soil organic carbon (SOC) (< 10 g kg^−1^, 10~15 g kg^−1^ and > 15 g kg^−1^), soil bulk density (< 1.3 g cm^−3^, 1.3~1.45 g cm^−3^ and > 1.45 g cm^−3^) and soil pH (< 6.5, 6.5~7.5, 7.5~8.5 and > 8.5). (3) Agricultural field management practices included water supply (< 200 mm, 200–400 mm, 400–600 mm, and > 600 mm), irrigation amount (< 100 mm, 100–200 mm, and > 200 mm), and nitrogen application rate (NAR) (< 100 kg ha^−1^, 100–200 kg ha^−1^, 200–300 kg ha^−1,^and > 300 kg ha^−1^); straw return amount (< 5 t ha^−1^, 5–10 t ha^−1,^and > 10 t ha^−1^ for SR; < 5 t ha^−1^, 5–10 t ha^−1^, 10–20 t ha^−1^, 20–30 t ha^−1,^and > 30 t ha^−1^ for BA); straw C/N ratio (< 50, 50–75, and > 75 for SR; < 50, 50–100, and > 100 for BA); and straw return and biochar application duration (1 year, 2 years, and ≥ 3 years).

### Meta-analysis

The formulas for calculating effect size, variance, weighting factor, weighted mean response ratio, 95% confidence interval, percentage change, WP, and PFPN are presented in **Equations 1**–**9**. If yield, WP, and PFPN statistics were not reported with a standard deviation (SD), we estimated SD using the following approach (Rusinamhodzi et al., 2011):

(1) $$\begin{array}{c} SD=X\times CV \end{array}$$

where X is the mean of the treatment (straw return or biochar application) or control group (no straw or no biochar), and CV is the variation coefficient. The responses of wheat yield, WP, and PFPN under SR and BA were quantified using the effect size (lnR) (Hedges et al., 1999):

(2) $$\begin{array}{c} lnR=\text{l}\text{n}(\frac{X_{e}}{X_{C}})=\text{l}\text{n}(X_{e})-\text{l}\text{n}(X_{c}) \end{array}$$

where $X_{e}$ and $X_{C}$ are the mean values of wheat yield, WP, and PFPN in the treatment group (straw return or biochar application) and the control group (no straw or no biochar).

The variance of lnRR (v) was calculated by the following formula (Hedges et al., 1999):

(3) $$\begin{array}{c} v=\frac{SD_{t}^{2}}{n_{t}X_{t}^{2}}+\frac{SD_{c}^{2}}{n_{c}X_{c}^{2}} \end{array}$$

where SDt, SDc, nt, and nc are the standard deviations and sample sizes of the treatment (SR or BA) and control (no straw or no biochar) groups, respectively.

Overall effects were estimated using intercept-only multilevel random-effects models. Subgroup analyses were performed using multilevel mixed-effects models, in which subgroup variables were included as fixed-effect moderators, and publication and comparison identity were included as random effects. Accordingly, the overall model was specified as follows:

(4) $$y_{ij}=\beta_{0}+u_{j}+r_{ij}+\epsilon_{ij}$$

The sampling variance of each effect size was calculated as follows (Parsons, 2003):

(5) $$v_{ij}=\frac{SD_{E,ij}^{2}}{n_{E,ij}X_{E,ij}^{2}}+\frac{SD_{C,ij}^{2}}{n_{C,ij}X_{C,ij}^{2}}$$

For the subgroup analyses, subgroup variables were entered as fixed-effect moderators in a multilevel mixed-effects model:

(6) $$y_{ij}=\beta_{0}+\sum_{m=1}^{p}\beta_{m}Z_{m,ij}+u_{j}+r_{ij}+\epsilon_{ij}$$

The effect size $\ln\bar{R}$ was converted into the percentage change as follows:

(7) $$E=(e^{ln\bar{R}}-1)\times 100\%$$

WP refers to the ratio of wheat yield to evapotranspiration (Thompson, 2023):

(8) $$WP=\frac{Y}{Total\;Winput}$$

where WP (kg ha^−1^) is water productivity, W_input_ is total water input (mm), and Y (t ha^−1^) is wheat yield.

(9) $$PFPN=\frac{Y}{TN}$$

where PFPN (kg ha^−1^) is partial factor productivity of nitrogen, TN is the total nitrogen fertilizer applied (kg ha^−1^), and Y (t ha^−1^) is wheat yield (Fernandez, 2023).

### Random forest model

The formulas for the random forest model are presented in **Equations 10**–**13**. The random forest model is an integrated machine learning method based on the bagging algorithm. It has high precision and good data-fitting performance. Random forest regression was separately performed for yield, WP, and PFPN as response variables, with environmental and management factors included as predictor variables. Subsequently, the model captures non-linear relationships between the response and predictor variables and quantifies the relative importance of environmental and management factors under SR and BA, thereby identifying the major factors associated with variations in yield, WP, and PFPN. The relative importance of the variables was assessed in RStudio (Version 4.4.1) using the R language “RandomForest” package. The number of decision trees was set to 1,000, and the number of variables randomly sampled as candidates for splitting at each node was set to three. In addition, five-fold cross-validation was performed to evaluate the predictive performance and robustness of the random forest model.

(10) $$\hat{Y}=\frac{1}{n}\sum_{i=1}^{n}T_{i}(X)$$

where $\hat{Y}$ represents the predicted value, $T_{i}(X)$ denotes the result of the prediction of the i-th decision tree, and n is the total number of decision trees (set to 1,000 in the study).

(11) $$OOB_{\text{error\_reg}}=\frac{1}{n}\sum_{i=1}^{n}{\left(\hat{Y_{i}}-Y_{i}\right)}^{2}$$

(12) $$OOB_{\text{error\_cla}}=\frac{1}{n}\sum_{i=1}^{n}1_{{\hat{Y}}_{i}\neq Y_{i}}$$

The OOB_error_ is out-of-bag (OOB), an internal estimator of prediction error, and the aim is to minimize this error.

(13) $$\begin{array}{c} I_{VIM_{i}}=\frac{\sum (E_{\text{errOOB}2}-E_{\text{errOOB}1})}{n_{\text{tree}}} \end{array}$$

where $I_{VIM_{i}}$ represents the importance of variable I; *E_e_*_rr_*_OOB_*_1_ represents the OOB error before random noise is added to all the variables in the OOB set; and *E_e_*_rr_*_OOB_*_2_ represents the OOB error after random noise is added (Breiman, 2001).

Between-comparison heterogeneity was evaluated using Cochran’s Q statistic, I², and the between-comparison variance τ². The Q statistic tests whether the dispersion of observed effect sizes exceeds that expected from sampling error alone, whereas I² describes the proportion of total variability attributable to heterogeneity rather than sampling error. The τ² parameter was estimated by restricted maximum likelihood (REML), and pooled lnRR values were calculated using a random-effects model. Substantial heterogeneity was detected in all six overall analyses (**Table 1**). Rosenthal’s fail-safe number was used to examine the possibility of publication bias. If the fail-safe number was > 5n + 10, this was considered to indicate that publication bias was unlikely to affect the credibility of the conclusion. We calculated the fail-safe numbers for SR and BA, respectively. The dataset comprised 5,469 observation pairs, and the fail-safe number was much larger than 5n + 10.

**Table 1:** Overall random-effects estimates and heterogeneity statistics for straw return and biochar application.

SR/BA	Y/wheat	N	Pooled change (95% CI)	Q (df)	P_Q_	I² (%)	τ²
Straw return	Yield	1388	8.31(6.93~9.72)	17,499.05 (1,387)	<0.001	92.07	0.01632
Straw return	WP	843	8.76 (6.59~10.99)	13,014.21 (842)	<0.001	93.53	0.02435
Straw return	PFPN	1225	8.28 (6.86~9.73)	9,405.75 (1,224)	<0.001	86.99	0.01683
Biochar application	Yield	854	10.34 (8.52~12.2)	10,121.49 (853)	<0.001	91.57	0.01719
Biochar application	WP	396	11.48 (8.27~14.78)	2,913.36 (395)	<0.001	86.44	0.01944
Biochar application	PFPN	763	9.96 (8.03~11.44)	3,019.18 (762)	<0.001	74.76	0.01707

Effect sizes were calculated as lnRR = ln(ME/MC), where ME and MC are the experimental- and control-group means, respectively. The pooled percentage change was back-transformed as [exp(pooled lnRR) − 1] × 100. Sampling variance was calculated as SDE²/(NE × ME²) + SDC²/(NC × MC²). Between-comparison variance (τ²) was estimated by restricted maximum likelihood (REML), and pooled effects were estimated using a random-effects model. WP, water productivity; PFPN, partial factor productivity of nitrogen; n, number of treatment–control comparisons.

Overall, the publication-bias diagnostics did not indicate consistent asymmetry across all outcomes. Egger’s test indicated possible small-study effects for straw return yield (*p* = 0.007), straw return PFPN (*p* < 0.001), and biochar application PFPN (*p* = 0.033), whereas Begg’s test was significant only for biochar application PFPN (*p* = 0.040) (**Tables 2**, **3**). Thus, only biochar application PFPN was supported by both tests, while the evidence for the other outcomes was inconsistent. Substantial heterogeneity remained across the publication-level analyses (*I*² = 53.83%–87.52%), indicating that funnel-plot asymmetry may partly reflect differences in climate, soil properties, and management practices rather than publication bias alone (**Supplementary Figure S2**). Moreover, trim-and-fill produced only modest changes in the pooled estimates and imputed studies on the right side of the funnel plots. Therefore, the overall positive effects were robust. Leave-one-publication-out sensitivity analyses showed that the pooled effects were not driven by any single title-defined publication (**Supplementary Figure S3**). Differences among subgroup levels were evaluated using omnibus Wald tests from multilevel mixed-effects meta-regressions. The resulting Q_b_ statistic was evaluated against a chi-square distribution with degrees of freedom equal to the number of subgroup levels minus one. Detailed results are presented in **Supplementary Tables S3** and **S4**. Between-subgroup heterogeneity tests were conducted to examine whether effect sizes differed among subgroups. The Qb statistic and its P value were calculated for each explanatory variable. The results for SR and BA are shown in **Tables 4** and **5**, respectively.

**Table 2:** Egger's regression and Begg's rank-correlation tests for small-study effects.

Amendment	Response	Egger's regression			Begg's test		
		Intercept	z	p	Kendall's tau	z	p
Straw return	Yield	-0.445	-2.676	0.007	0.018	0.449	0.653
Straw return	WP	0.032	0.080	0.936	-0.044	-0.846	0.397
Straw return	PFPN	-1.238	-6.653	<0.001	0.031	0.730	0.466
Biochar application	Yield	-0.248	-0.939	0.348	0.002	0.028	0.977
Biochar application	WP	0.306	0.638	0.523	-0.110	-1.269	0.204
Biochar application	PFPN	-3.224	-2.129	0.033	-0.119	-2.054	0.040

Egger's test evaluates whether the regression intercept differs from zero. Begg's test is based on Kendall's rank correlation. Bold p values indicate p < 0.05. Statistical asymmetry indicates possible small-study effects and does not, by itself, prove publication bias. Egger’s test indicated funnel-plot asymmetry for straw return yield (p = 0.007), straw return PFPN (p < 0.001), and biochar application PFPN (p = 0.033). Begg’s test was significant only for biochar application PFPN (p = 0.040), whereas neither test indicated asymmetry for the other three outcomes. The trim-and-fill procedure imputed 17, 19, and 10 studies for straw return yield, straw return PFPN, and biochar application PFPN, respectively. Their pooled RRs changed only moderately from 1.083 to 1.097, 1.084 to 1.101, and 1.097 to 1.115. Because the imputed studies were located on the right side of the funnel plots, the adjustment increased rather than reduced the effects and did not show the conventional pattern of missing small negative studies.

**Table 3:** Effect sizes before and after the trim-and-fill sensitivity analysis.

Amendment	Response	Original dataset				Filled studies	Trim-and-fill adjusted dataset			
		p	RR	95% CI	I² (%)		p	RR	95% CI	I² (%)
Straw return	Yield	<0.001	1.083	[1.069, 1.097]	78.50	17	<0.001	1.097	[1.082, 1.112]	81.27
Straw return	WP	<0.001	1.083	[1.061, 1.105]	87.52	0	<0.001	1.083	[1.061, 1.105]	87.52
Straw return	PFPN	<0.001	1.084	[1.069, 1.098]	65.81	19	<0.001	1.101	[1.085, 1.117]	70.80
Biochar application	Yield	<0.001	1.101	[1.083, 1.118]	83.40	0	<0.001	1.101	[1.083, 1.118]	83.40
Biochar application	WP	<0.001	1.112	[1.080, 1.144]	65.13	0	<0.001	1.112	[1.080, 1.144]	65.13
Biochar application	PFPN	<0.001	1.097	[1.077, 1.117]	53.83	10	<0.001	1.115	[1.093, 1.137]	62.94

*Notes:* RR, response ratio (exp[lnRR]); CI, confidence interval; I², percentage of total variability attributable to heterogeneity. Filled studies are the number of studies imputed by the Duval-Tweedie trim-and-fill procedure. Trim-and-fill was applied only when Egger's or Begg's test indicated asymmetry (p < 0.05); when no asymmetry was detected, no studies were filled and the adjusted estimate equals the original estimate. Analyses were conducted at the publication level after aggregating correlated comparisons within publications using an assumed within-publication correlation of rho = 0.8.

The trim-and-fill analysis imputed 17, 19, and 10 potentially missing studies for straw return yield, straw return PFPN, and biochar application PFPN, respectively. The corresponding pooled RRs increased moderately from 1.083 to 1.097, from 1.084 to 1.101, and from 1.097 to 1.115. No missing studies were imputed for the other three outcomes, and their pooled estimates remained unchanged. All adjusted confidence intervals remained above 1.0 (p < 0.001), indicating that the directions and statistical significance of the overall effects were robust to the trim-and-fill sensitivity analysis. However, heterogeneity remained substantial and the imputed studies occurred on the right side of the funnel plots; therefore, the adjusted estimates should be regarded as sensitivity results rather than definitive bias-corrected effects.

**Table 4:** Between-subgroup heterogeneity tests for straw return/SR.

Factor class	Explanatory variable	Yield		WP		PFPN	
		Qb	P value	Qb	P value	Qb	P value
Climate factors	Growing-season precipitation	4.33	0.228	5.09	0.165	2.14	0.543
	Growing-season temperature	1.79	0.408	0.86	0.652	1.91	0.385
Soil physicochemical factors	Soil texture	4.37	0.112	2.41	0.299	3.06	0.217
	Soil organic carbon	2.14	0.344	3.88	0.144	2.42	0.298
	Soil bulk density	5.16	0.076	5.62	0.060	4.99	0.082
	Soil pH	2.05	0.561	2.60	0.457	1.79	0.618
Field managements	Water supply	2.63	0.452	1.53	0.675	8.27	0.041
	Irrigation amount	0.01	0.996	0.83	0.659	0.19	0.908
	Nitrogen application rate	10.12	0.018	4.21	0.239	9.27	0.026
	Straw application rate	0.77	0.679	0.40	0.820	2.42	0.298
	Straw C/N ratio	2.94	0.229	1.31	0.518	1.94	0.379
	Experimental duration	0.43	0.806	1.18	0.554	0.04	0.980

**Table 5:** Between-subgroup heterogeneity tests for biochar application/BA.

Factor class	Explanatory variable	Yield		WP		PFPN	
		Qb	P value	Qb	P value	Qb	P value
Climate factors	Growing-season precipitation	2.74	0.433	3.21	0.361	2.98	0.394
Climate factors	Growing-season temperature	3.78	0.151	5.22	0.074	4.81	0.090
Soil physicochemical factors	Soil texture	5.74	0.057	1.55	0.460	4.91	0.086
Soil physicochemical factors	Soil organic carbon	0.49	0.784	0.23	0.892	0.23	0.893
Soil physicochemical factors	Soil bulk density	0.64	0.726	0.70	0.706	0.25	0.881
Soil physicochemical factors	Soil pH	1.23	0.746	1.96	0.581	2.14	0.544
Water and nitrogen management	Water supply	0.77	0.858	3.22	0.359	0.46	0.927
Water and nitrogen management	Irrigation amount	1.24	0.538	1.65	0.439	2.80	0.246
Water and nitrogen management	Nitrogen application rate	1.26	0.738	1.83	0.609	1.62	0.655
Amendment management	Biochar application rate	17.77	0.001	8.22	0.084	17.92	0.001
Amendment management	Biochar C/N ratio	0.30	0.862	0.38	0.827	0.77	0.682
Amendment management	Experimental duration	0.11	0.947	3.14	0.208	0.01	0.997

In this study, Excel 2021 was used for data collection, and MetaWin 2.1 was used for the integrated analysis. Origin 2024b and GraphPad Prism 9.5.0 were used to create graphs; the “LinkET” package was used for the correlation analysis, and the relative importance of the indicators for wheat yield, WP, and PFPN was examined using the random forest model in R version 4.4.1.

## Results

### Overall analysis

A total of 385 published articles on the effects of straw return and biochar application on wheat yield, WP, and PFPN were collected for statistical analysis, including 247 studies on SR for wheat (1,388 pairs of yield, 843 pairs of WP, and 1,225 pairs of PFPN) and 138 studies on BA for wheat (854 pairs of yield, 396 pairs of WP, and 763 pairs of PFPN). The global meta-analysis showed that SR and BA significantly increased wheat yield by 8.31% and 10.34%, WP by 8.76% and 11.48%, and PFPN by 8.28% and 9.96%, respectively (**Table 6**). Additionally, the frequency distributions of effect sizes for wheat yield, WP, and PFPN under SR and BA followed Gaussian normal distributions (**Figure 2**). For SR and BA, the line regression revealed significant positive correlations between wheat yield and WP and between wheat yield and PFPN (**Figures 3a, b**). The straw return WP had a positive correlation with PFPN; however, the biochar application WP had no relationship with PFPN (**Figure 3c**).

**Table 6:** Effect size of SR and BA on wheat yield, WP and PFPN.

Index	Treatments	Effect size	Numbers	Fail-safe number
Wheat Yield	SR BA	8.31(6.93~9.72) 10.34 (8.52~12.2)	1388 854	1928432.9 1512891
Wheat WP	SR BA	8.76 (6.59~10.99) 11.48 (8.27~14.78)	843 396	608373.3 204071.4
Wheat PFPN	SR BA	8.28 (6.86~9.73) 9.96 (8.03~11.44)	1225 763	788394.8 326580.1

SR, straw return; BA, biochar application; WP, water productivity; PFPN, partial factor productivity of nitrogen.

**Figure 2:**
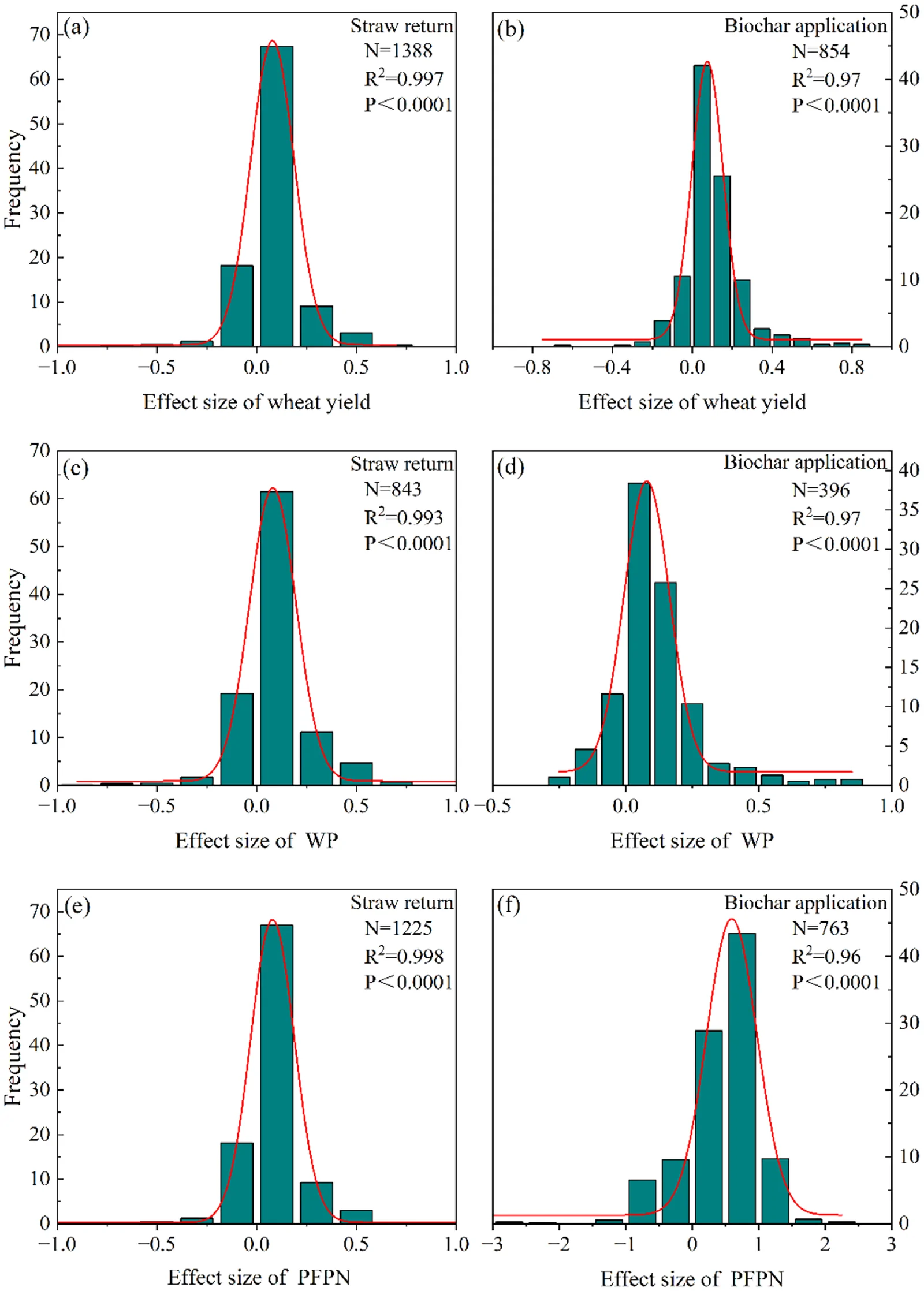
Frequency distributions of the effect sizes of straw return and biochar application on wheat yield **(a, b)**, WP **(c, d)**, and PFPN **(e, f)**. The red line represents the Gaussian normal distribution; n denotes the sample size, and *p* denotes the significance level.

**Figure 3:**
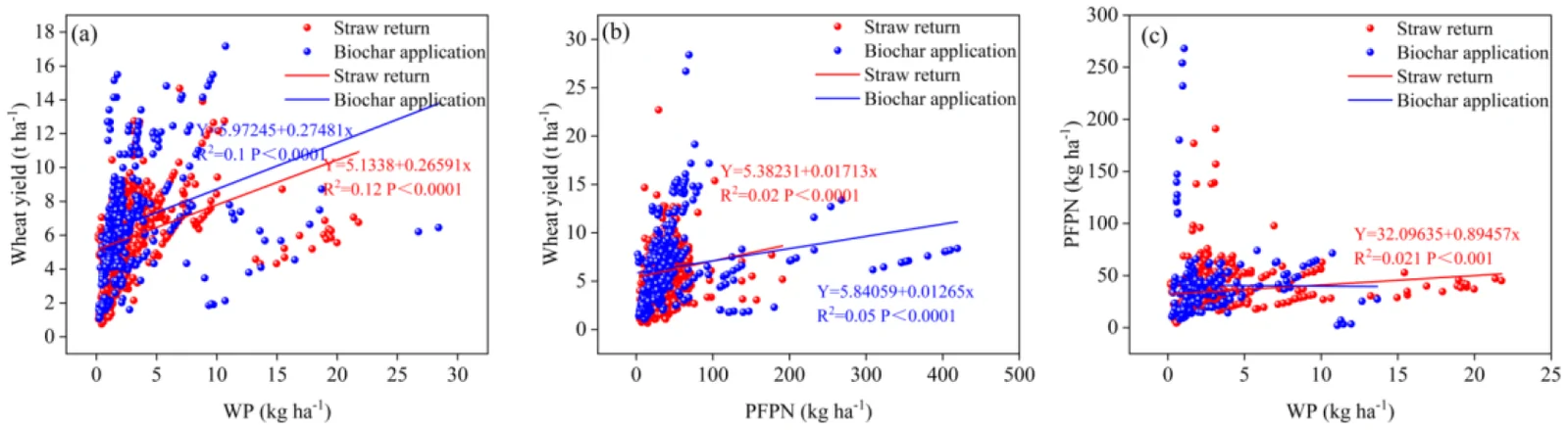
Relationships between **(a)** wheat yield and water productivity (WP), **(b)** wheat yield and partial factor productivity of nitrogen (PFPN), and **(c)** PFPN and WP. Red dots represent straw return (SR), and blue dots represent biochar application (BA). The significant R² and p-values for the models are shown.

### Effects of straw return and biochar application under various climatic conditions on wheat yield, WP, and PFPN

The effects of SR and BA on wheat yield, WP, and PFPN varied with changing climatic factors (**Figure 4**). Compared with no straw and no biochar application, the SR and BA experiments notably increased average yield, WP, and PFPN by 8.61% and 11.25%, 9.86% and 12.87%, 9.12% and 41.56% in GSP subgroups (**Table 6**), respectively. As GSP increased, the impact of SR and BA on wheat yield, WP, and PFPN originally increased and then decreased. In particular, when GSP was < 450 mm (**Figures 4a–f**), SR and BA significantly improved wheat yield, WP, and PFPN. The yield of SR increased with increasing growing season precipitation (**Figure 5a**), but this pattern was not observed for BA (**Figure 5b**). WP under SR and BA was negatively correlated with increasing growing season precipitation (**Figures 5c, d**). In addition, PFPN showed no clear relationship with growing season precipitation under SR or BA (**Figures 5e, f**).

**Figure 4:**
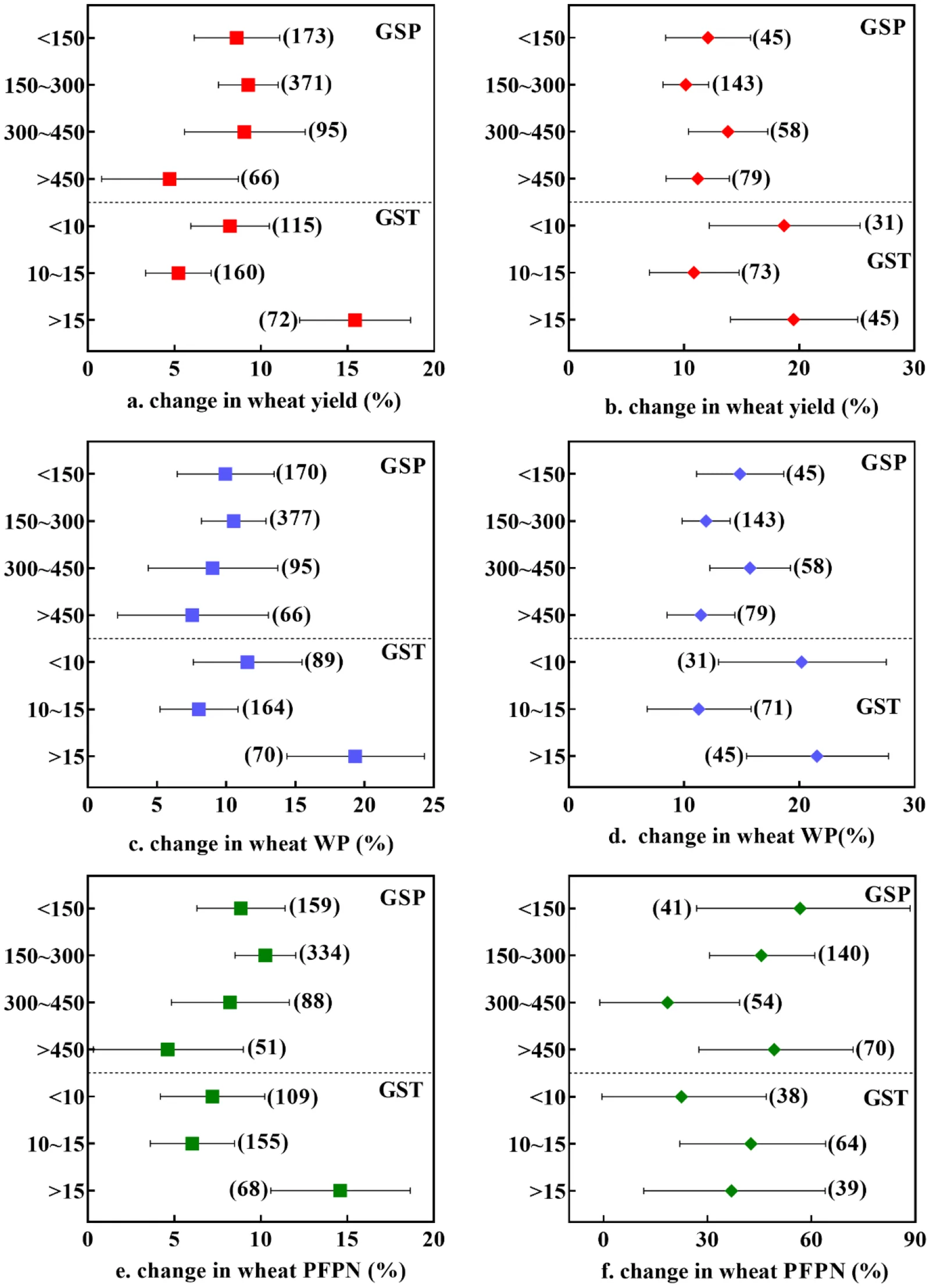
Effects of straw return and biochar application on **(a, b)** wheat yield, **(c, d)** water productivity, and **(e, f)** partial factor productivity of nitrogen under various growing season precipitation categories (< 150 mm, 150–300 mm, 300–450 mm, and > 450 mm) and growing season temperature categories (< 10 °C, 10 °C–15 °C and > 15 °C). The mean effect value and 95% confidence intervals are presented. Sample sizes (n) are indicated adjacent to each bar.

**Figure 5:**
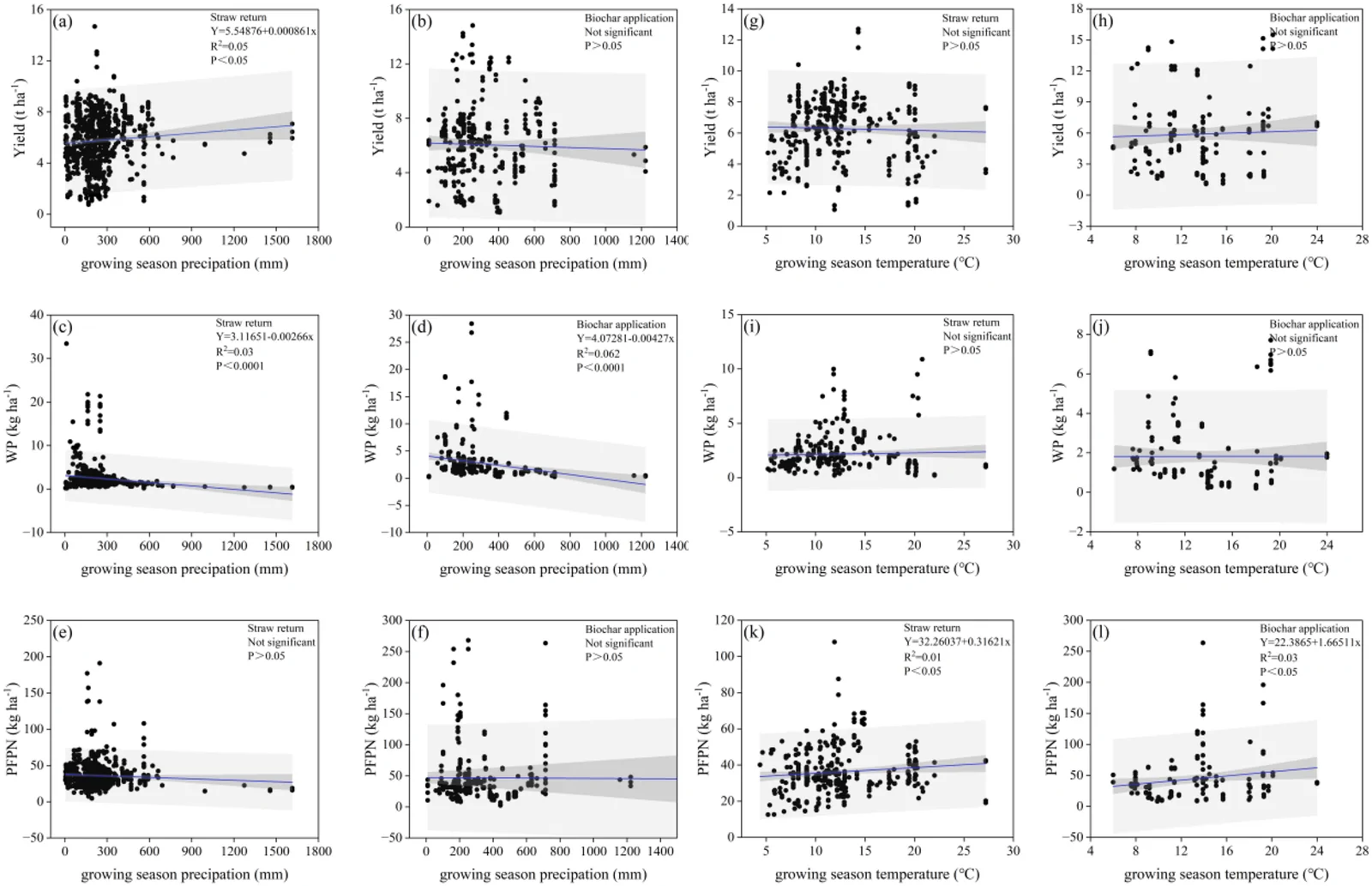
Relationships between growing season precipitation (mm) and **(a, b)** wheat yield, **(c, d)** water productivity, and **(e, f)** partial factor productivity of nitrogen under straw return (SR). Relationships between growing season temperature (°C) and **(g, h)** wheat yield, **(i, j)** water productivity, and **(k, l)** partial factor productivity of nitrogen with biochar application (BA).

Both SR and BA notably enhanced wheat yield, WP, and PFPN across all growing season temperature categories (**Figures 4a–f**). Compared with no straw and biochar application, the yield, WP, and PFPN of SR and BA grew as the GST increased, especially when growing season temperature was > 15 °C, SR and BA increased yield, WP, and PFPN were higher than the effect size for GST < 10 °C and 10 °C–15 °C, except PFPN under BA at GST 10 °C–15°C (**Figure 4**). PFPN increased linearly with increasing growing season temperature under SR and BA (**Figures 5k, l**), but no such linear relationship was observed for yield or WP under SR and BA treatments (**Figures 5a–d**). BA had significantly higher effect sizes for yield, WP, and PFPN than SR across the field experiments, regardless of GSP or GST (**Figure 4**).

### Effects of straw return and biochar application under various soil properties on wheat yield, WP, and PFPN

**Figure 6** shows the effects of SR and BA on wheat yield, WP, and PFPN varied with initial soil properties. Compared with no straw and biochar application, both SR and BA significantly increased wheat yield, WP, and PFPN in coarse and fine soils under most conditions. As SOC, BD, soil pH increased, the effects of SR and BA on wheat yield, WP, and PFPN initially increased and subsequently decreased. SR and BA had higher positive effects on wheat yield, WP, and PFPN (except for WP under SR at SOC > 15 g kg^−1^) (**Figure 6c**). Compared with high BD levels (BD > 1.45 g cm^−3^), SR and BA had greater effects on wheat yield, WP, and PFPN under lower BD levels (BD < 1.45 g cm^−3^). By contrast, the effect of BA on PFPN was most pronounced at BD > 1.45 g cm^−3^. The positive effect of SR on wheat yield and PFPN was highest at pH < 6.5; an opposite trend was observed for WP, which was notably elevated at pH 7.5–8.5 compared with no straw. The improvements in wheat yield and WP under BA were greater at pH < 6.5; in contrast, the effect of BA on PFPN was greater at pH > 8.5. The calculated fail-safe numbers for wheat yield, WP, and PFPN under BA were 1,512,891, 204,071.4, 6,434,565.3, respectively (**Table 6**), suggesting that the results were relatively robust to publication bias.

**Figure 6:**
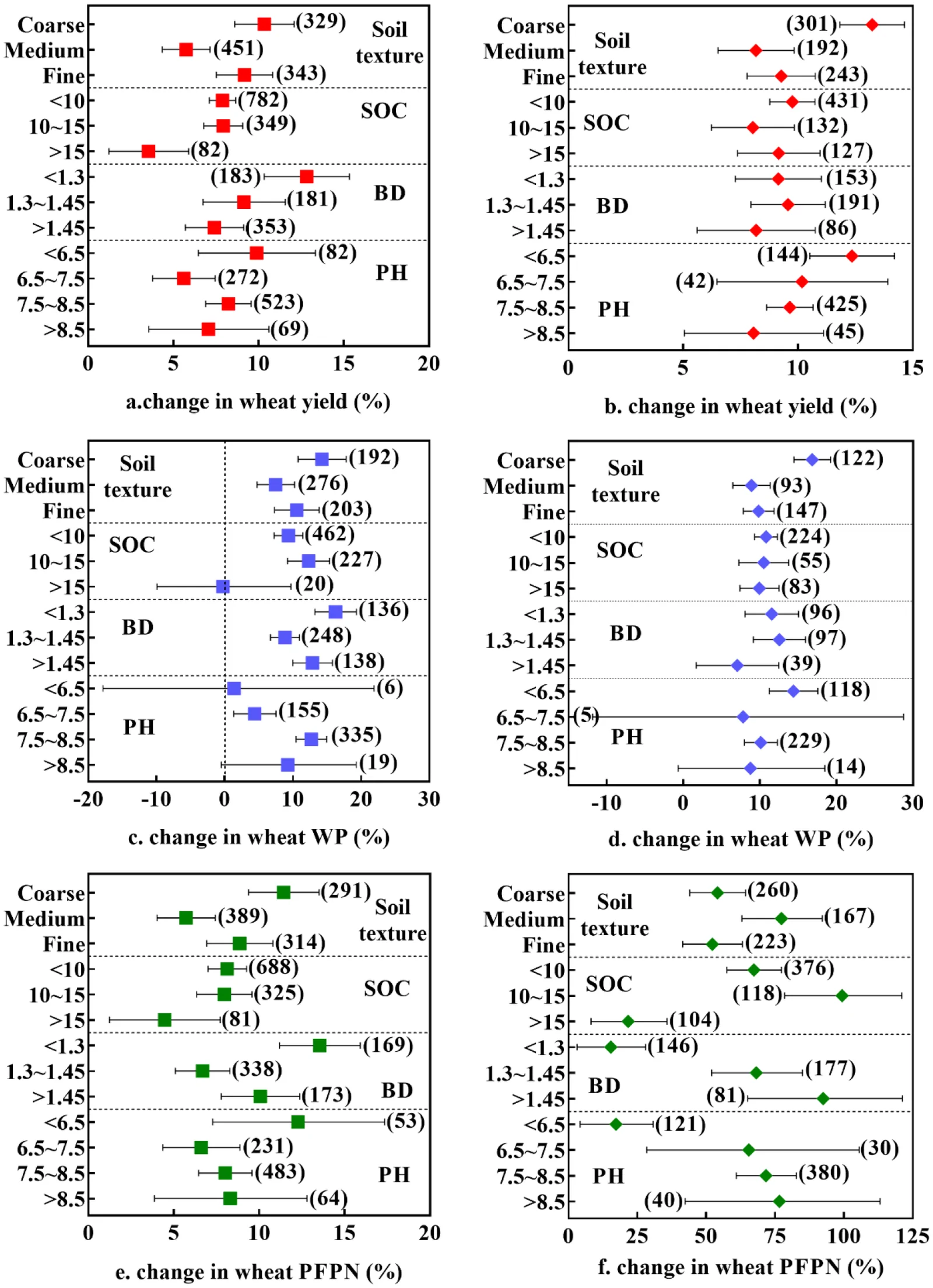
Effects of straw return and biochar application on **(a, b)** wheat yield, **(c, d)** water productivity (WP), and **(e, f)** partial factor productivity of nitrogen (PFPN) under various soil textures (coarse, medium, and fine), soil organic carbon (< 10 g kg^−1^, 10–15 g kg^−1,^and > 15 g kg^−1^), bulk density (< 1.3 g cm^−3^, 1.3–1.45 g cm^−3,^ and > 1.45 g cm^−3^), and pH (< 6.5, 6.5–7.5, 7.5–8.5, and > 8.5). The mean effect value and 95% confidence intervals are presented. Sample sizes (n) are indicated adjacent to each bar.

### Effects of straw return and biochar application under various agricultural field-management practices on wheat yield, WP, and PFPN

**Figure 7** shows the remarkably positive effects of various agricultural practices on wheat yield, WP, and PFPN under SR and BA in field experiments. The effects of SR on wheat yield, WP, and PFPN were greater at water supply levels of < 200 mm and > 600 mm. Conversely, the effects of BA on wheat yield, WP, and PFPN were greater at water supply levels < 600 mm. Both SR and BA significantly enhanced yield, WP, and PFPN at each irrigation level. Under SR, an irrigation amount < 100 mm had the greatest positive effects on yield (8.84%), WP (11.85%), and PFPN (7.61%), with larger effect sizes than those at irrigation amounts of 100–200 and > 200 mm. In contrast, as irrigation amount increased, the effects of BA on yield, WP, and PFPN showed an increasing trend (**Figures 7b, d, f**). SR and BA significantly improved yield, WP, and PFPN at most nitrogen application rate (NAR) levels, except for WP under SR at NAR < 100 kg ha^−1^ and WP under BA at NAR > 300 kg ha^−1^; however, no significant differences were detected among NAR levels. Regression analysis of the two amendment methods showed that the effect size for yield increased with increasing water supply under SR (P < 0.05, **Figure 8a**), but not under BA (**Figure 8b**). WP under both SR and BA decreased with the increasing water supply (**Figures 8c, d**). Regression analysis revealed that the PFPN under SR and BA had no obvious linear correlation with water supply (**Figures 8e, f**). More importantly, the SR and BA yields increased remarkably with increasing NAR (P < 0.05, **Figures 8g, h**); however, PFPN decreased under SR and BA (**Figures 8k, l**). SR improved WP with increasing NAR, but this pattern was not observed under BA (**Figures 8i, j**).

**Figure 7:**
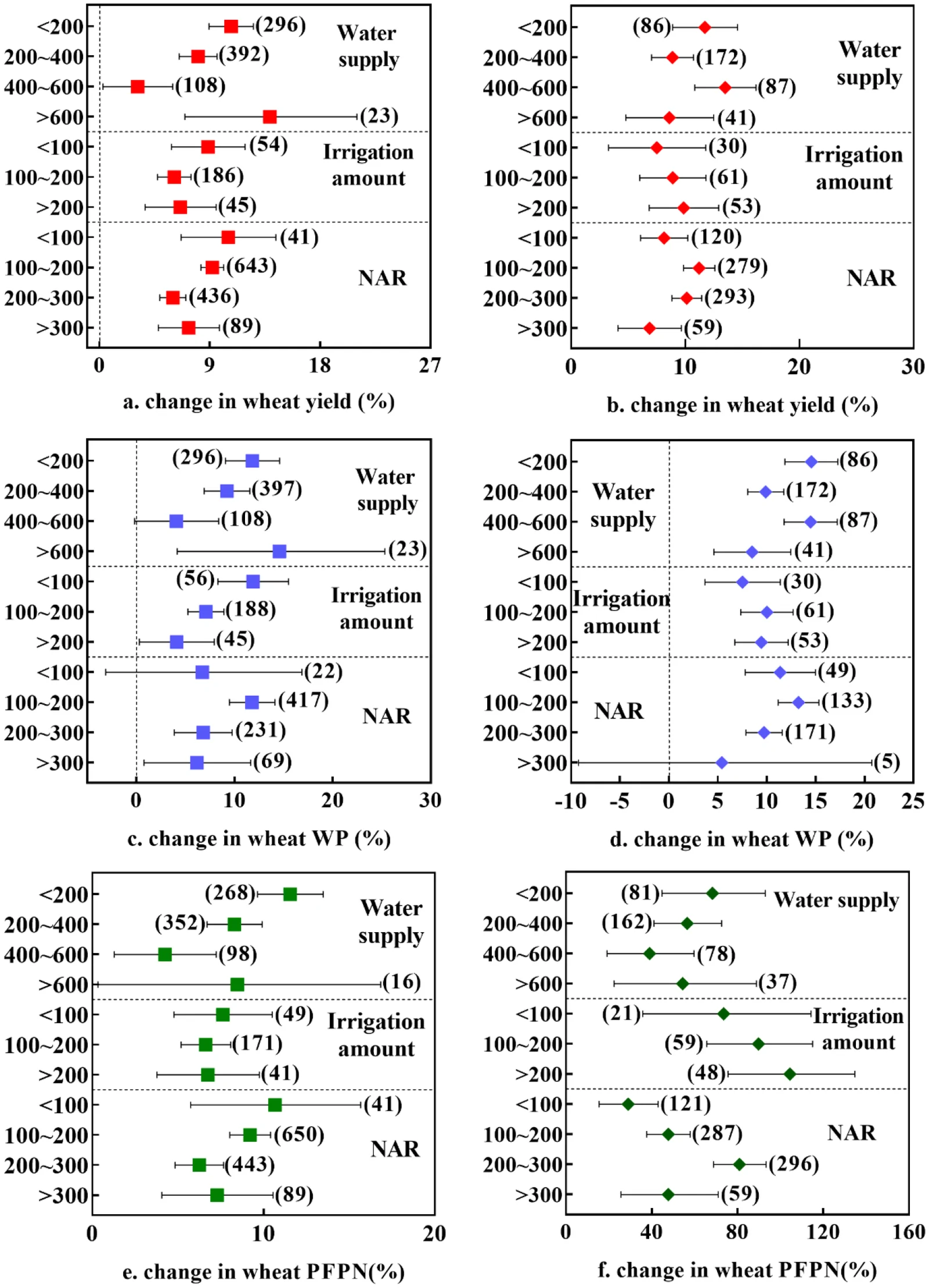
Effects of straw return and biochar application on **(a, b)** wheat yield, **(c, d)** water productivity (WP), and **(e, f)** partial factor productivity of nitrogen (PFPN) under various water supply levels (< 200 mm, 200–400 mm, 400–600 mm, and > 600 mm), irrigation amounts (< 100 mm, 100–200 mm, and > 200 mm), and nitrogen application rates (< 100 kg ha^−1^, 100–200 kg ha^−1^, 200–300 kg ha^−1,^and > 300 kg ha^−1^). The mean effect value and 95% confidence intervals are presented. Sample sizes (n) are indicated adjacent to each bar.

**Figure 8:**
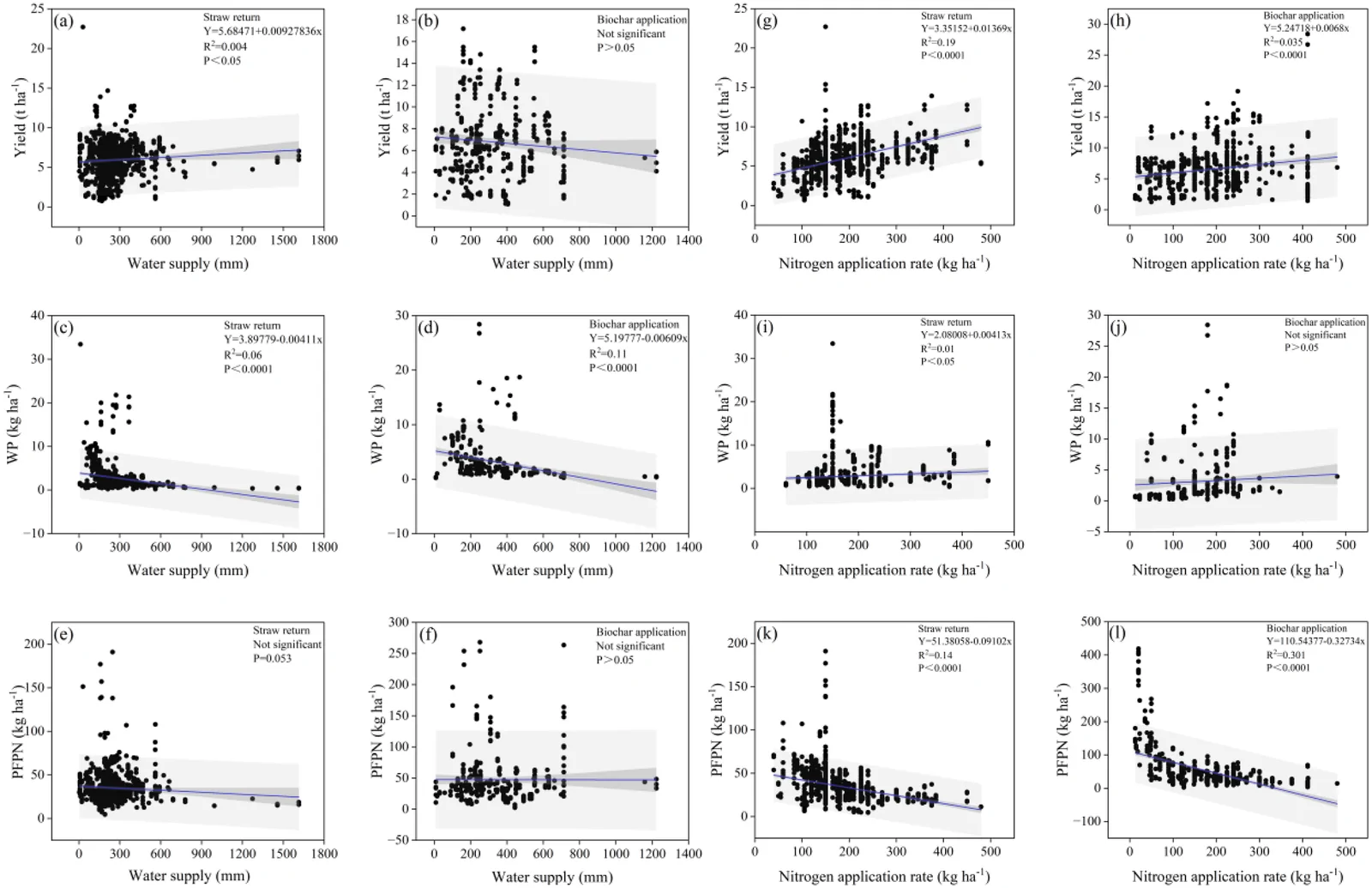
Relationships between **(a, b)** wheat yield, **(c, d)** water productivity (WP), and **(e, f)** partial factor productivity of nitrogen (PFPN) and water supply (mm) under straw return (SR) and biochar application (BA). Relationships between **(g, h)** wheat yield, **(i, j)** water productivity, and **(k, l)** partial factor productivity of nitrogen and nitrogen application rate (kg ha^-1^) (NAR) under straw return (SR) and biochar application (kg ha^-1^) (BA).

As shown in **Figure 9**, the properties and application duration of straw and biochar also influenced the responses of wheat yield, WP, and PFPN to SR and BA. SR and BA increased wheat yield, WP, and PFPN at all SRA and BAR levels. With increasing SRA, the effects of SR on wheat yield, WP, and PFPN and the effect of BA on PFPN initially increased and subsequently decreased (**Figures 9a, c, e, f**). In contrast, the effects of BA on yield and WP increased continuously (**Figures 9b, d**). The effects of SR on wheat yield and WP were greater at straw C/N ratios < 75, whereas the opposite pattern was observed for PFPN in **Figure 10c**. In addition, the effects of BA on wheat yield, WP, and PFPN were greatest when the biochar C/N ratio was ≥ 100. Both SR and BA enhanced wheat yield, WP, and PFPN across the three application-duration categories, with greater effects at shorter experimental durations of ≤ 2 years. Linear regression analysis indicated that only the effects of SR on wheat yield and WP increased with increasing SRA (**Figures 11a, c**); this pattern was not observed for the other outcomes (**Figures 11b, d, e, f**).

**Figure 9:**
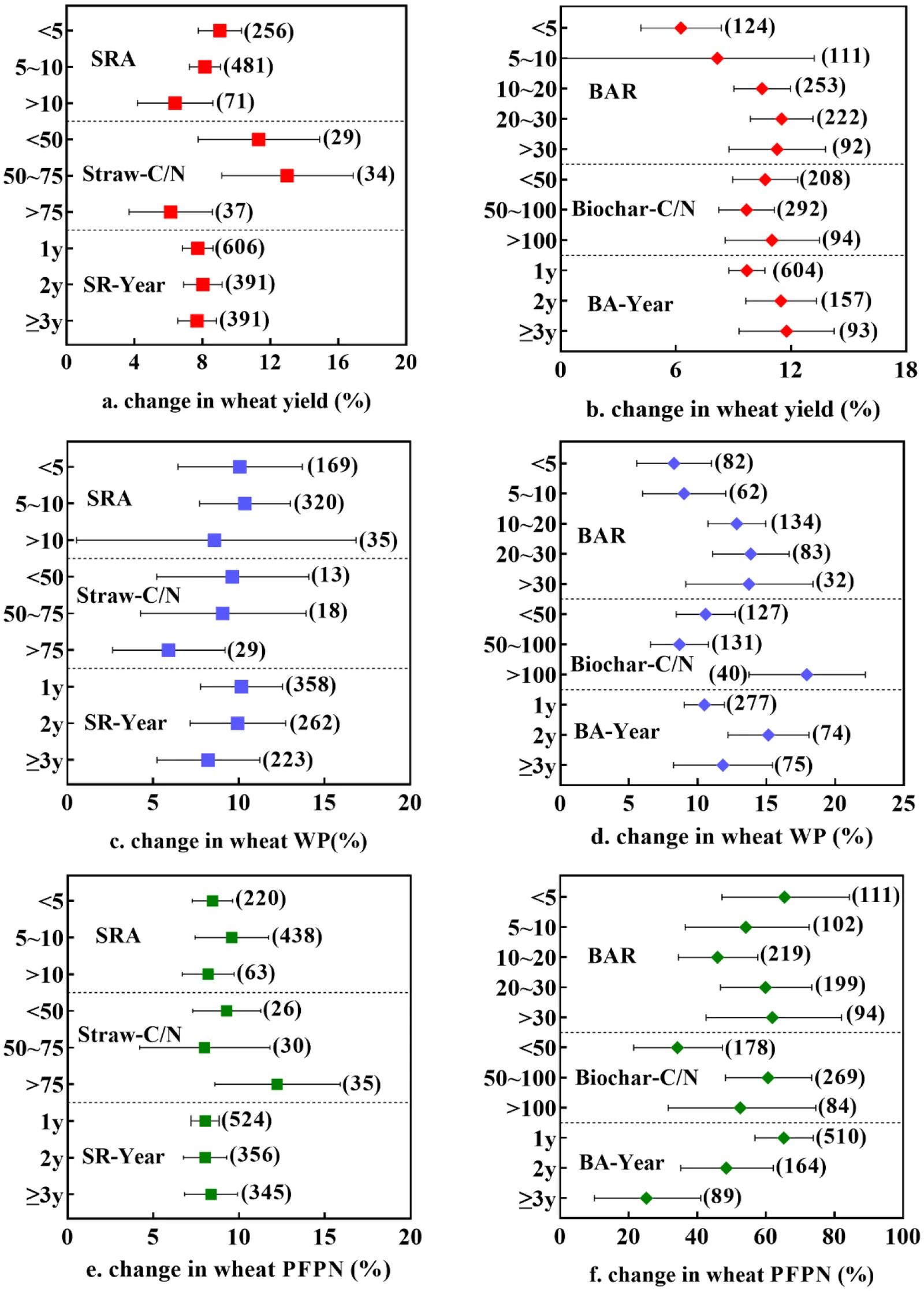
Effects of straw return on **(a)** wheat yield, **(c)** water productivity (WP), and **(e)** partial factor productivity of nitrogen (PFPN) under various straw return amounts (< 5 t ha^−1^, 5–10 t ha^−1,^ and > 10 t ha^−1^ for SR; < 5 t ha^−1^, 5–10 t ha^−1^, 10–20 t ha^−1^, 20–30 t ha^−1,^ and > 30 t ha^−1^ for BA), straw-C/N ratios (< 50, 50–75, and > 75 for SR; < 50, 50–100, and > 100 for BA), and straw return year (1 year, 2 years and ≥ 3 years). The mean effect value and 95% confidence intervals are presented. Sample sizes (n) are indicated adjacent to each bar.

**Figure 10:**
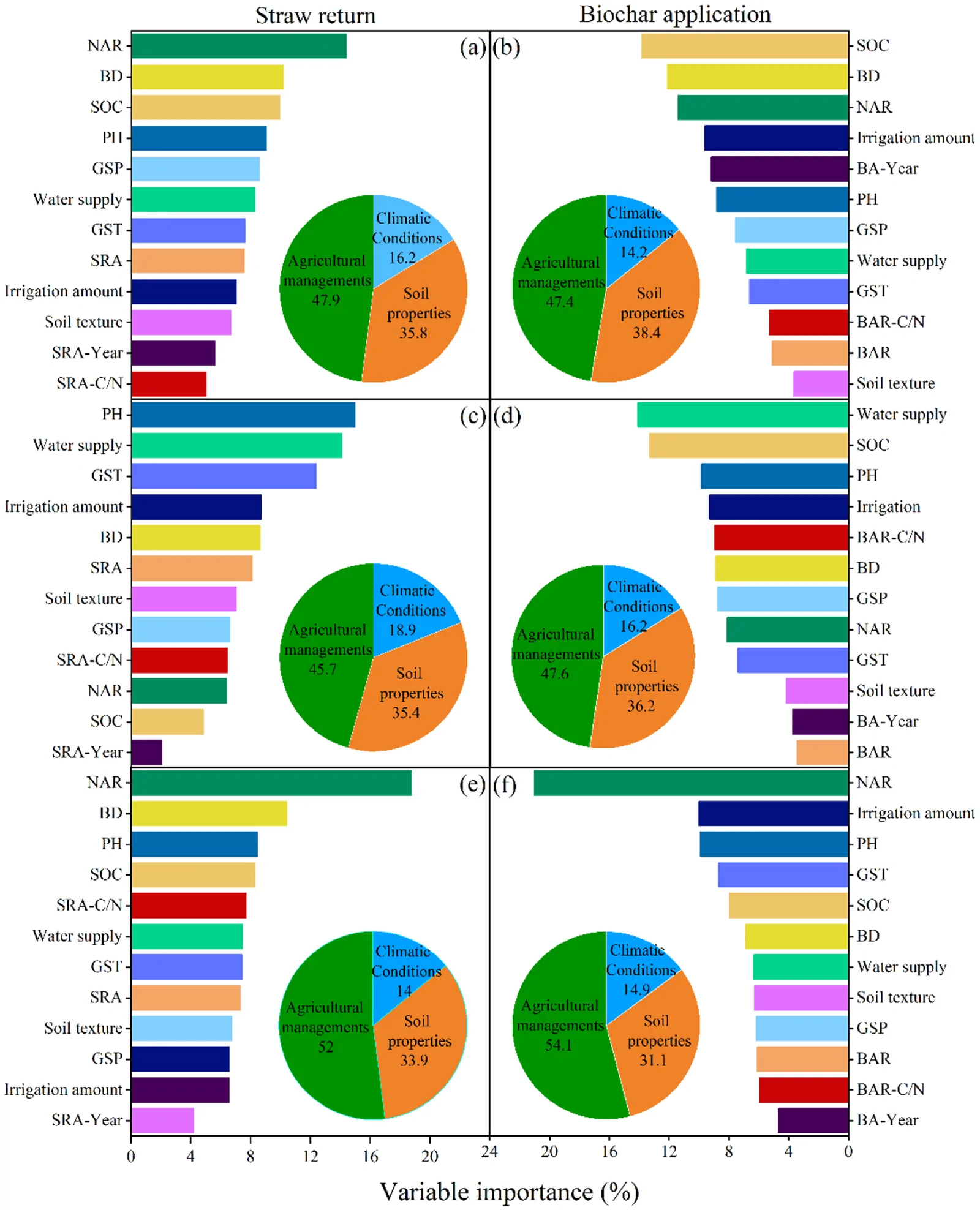
Variable importance for **(a, b)** yield, **(c, d)** WP, and **(e, f)** PFPN under straw return and biochar application. In the pie charts, green represents the cumulative importance of agricultural management-practice variables, brown denotes soil-property variables, and blue signifies climate variables.

**Figure 11:**
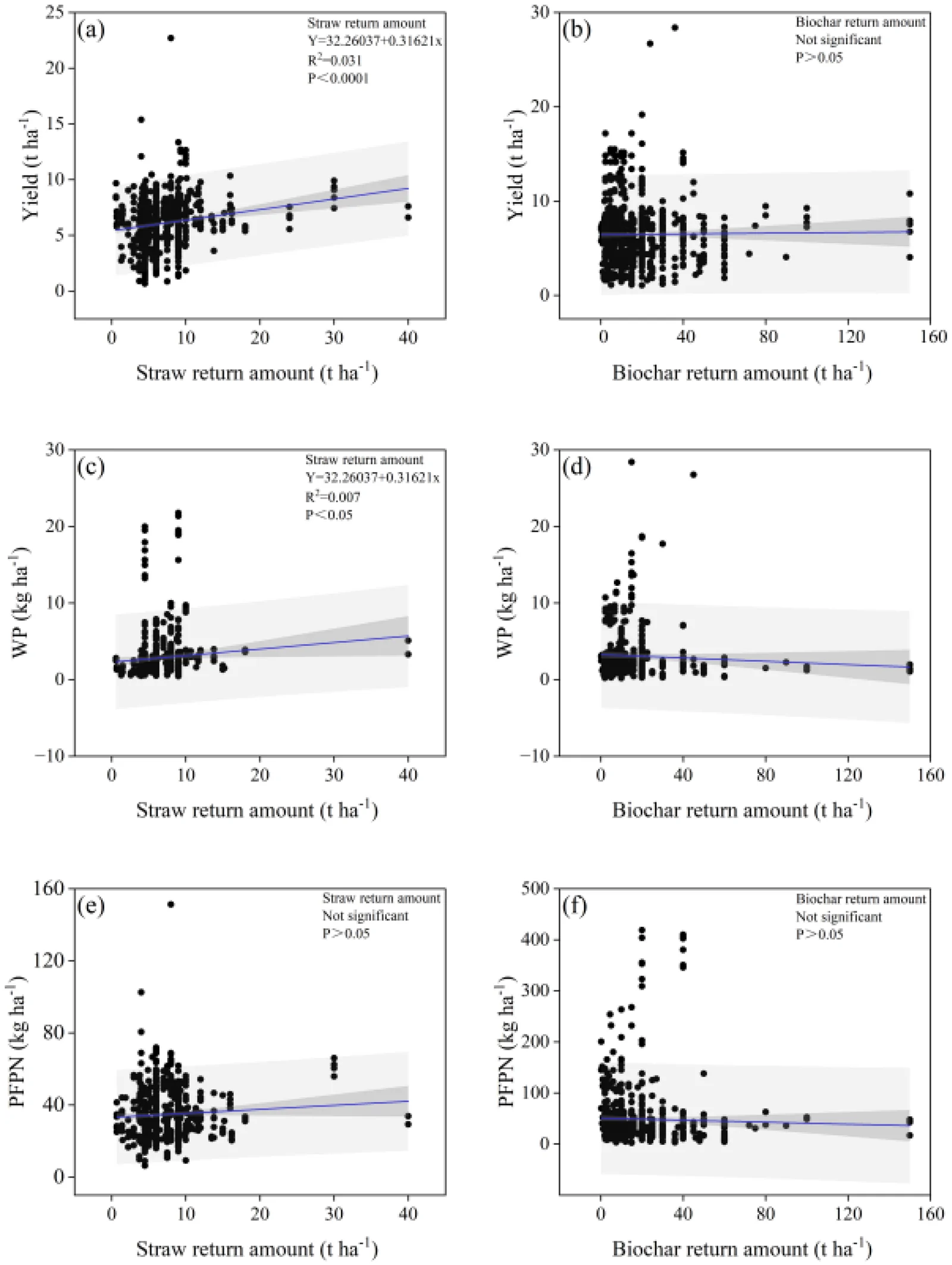
Relationships between straw return amount (t ha^-1^) and **(a)** wheat yield, **(c)** water productivity, and **(e)** partial factor productivity of nitrogen under straw return (SR). Relationships between biochar return amount (t ha^-1^) and **(b)** wheat yield, **(d)** water productivity, and **(f)** partial factor productivity of nitrogen under biochar application (BA). P < 0.001 and P < 0.05 indicate significant differences; P > 0.05 indicates no significant differences.

### Relative importance ranking of factors and correlation analysis influencing the effects on wheat yield, WP, and PFPN

Random forest models were used to identify the key factors affecting wheat yield, WP, and PFPN under SR and BA (**Figure 10**). The percentage of different variables for wheat yield, WP, and PFPN varied across indices. The relative importance of the primary three variables with the greatest impact on yield of SR were NAR (14.4%), BD (10.17%), and SOC (9.97%) respectively; the primary three variables influencing WP under SR were pH (14.95%), water supply (14.09%), and GST (12.36%), respectively; and the three most important variables influencing PFPN under SR were NAR (18.77%), BD (10.41%), and pH (8.48%), respectively. By contrast, the three most important variables influencing wheat yield under BA were SOC (13.81%), BD (12.13%), and pH (11.42%), respectively; the three most important variables influencing WP under BA were water supply (14.1%), SOC (13.31%), and pH (9.85%), respectively; and the three most important variables influencing PFPN under BA were NAR (21%), irrigation amount (10%), and pH (9.93%), respectively. In brief, agricultural management practices played a significant role in increasing wheat yield, improving WP, and enhancing PFPN under SR and BA (**Figures 10a–f**).

The correlation analysis results indicated that SR had a significant positive correlation with wheat yield, NAR, and irrigation amount, while being significantly negatively correlated with soil pH. The effect of SR on wheat WP was significantly positively correlated with soil texture and SRA but significantly negatively correlated with GSP and water supply. Wheat PFPN under SR was significantly correlated with the straw C/N ratio and GST (**Figures 12a, c, e**). The effect of BA on wheat yield was positively correlated with irrigation amount, BD, and NAR but significantly negatively correlated with BA application duration. The effect of BA on wheat WP was significantly positively correlated with soil pH and BD, while being significantly negatively correlated with water supply and GSP. The effect of BA on wheat PFPN was positively correlated with soil pH and BD but negatively correlated with GSP and water-nitrogen inputs (**Figures 12b, d, f**).

**Figure 12:**
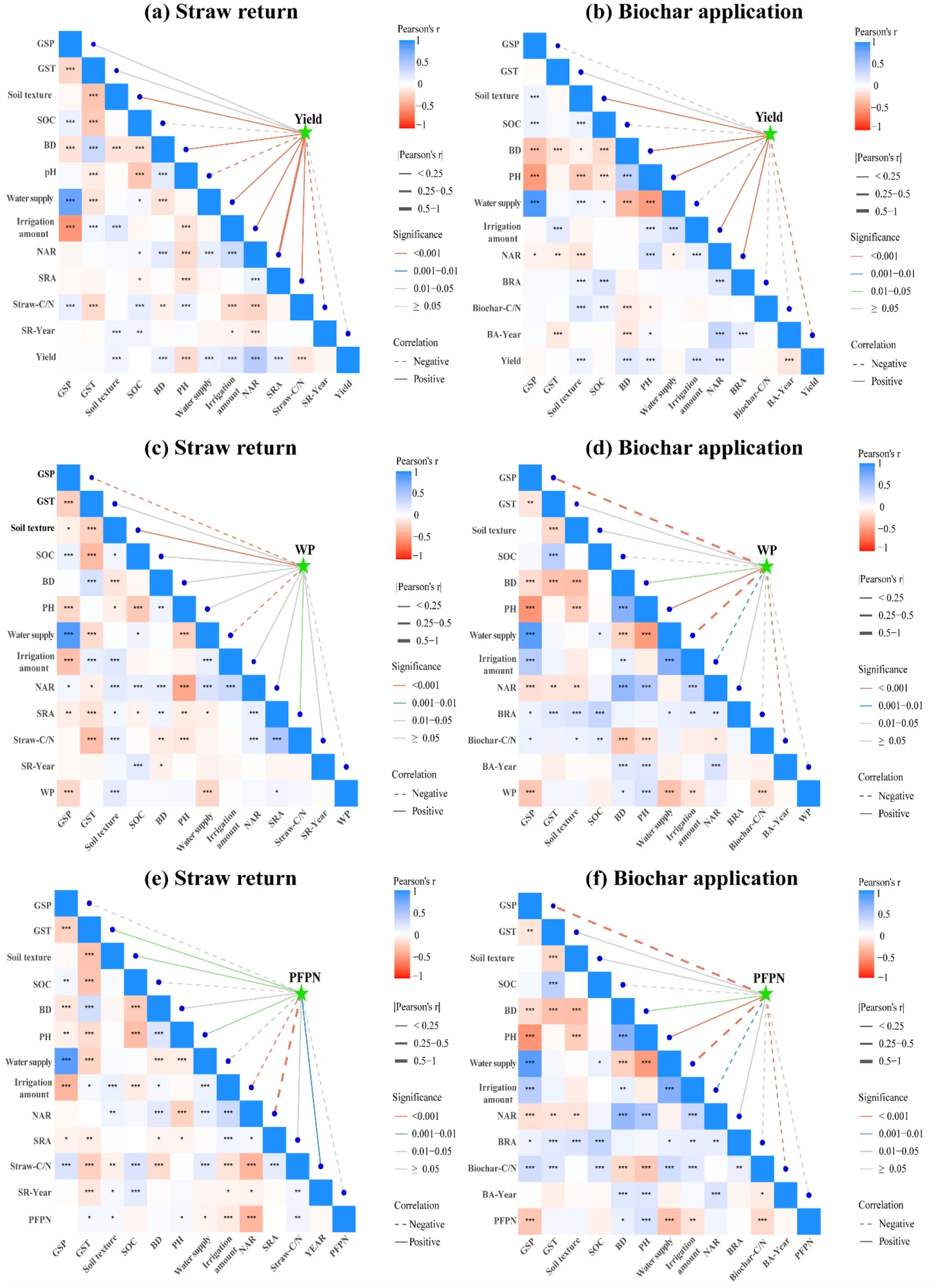
Correlation analysis of the effects of SR and BA on wheat yield, WP, and PFPN under different climatic conditions, soil properties, and agricultural field-management practices. Correlation analysis of the effects of SR and BA on wheat yield, WP, and PFPN under different climatic conditions, soil properties, and agricultural field-management practices. Panels **(a)**, **(c)**, and **(e)** present the results for wheat yield, WP, and PFPN, respectively, under SR, whereas panels **(b)**, **(d)**, and **(f)** present the corresponding results under BA. The lower-left portion of each panel shows the correlation analysis. The colors in the cells indicate significant correlations (*p < 0.05, **p < 0.01, and ***p < 0.001), and the color intensity indicates the Pearson rank correlation coefficient. The upper right panel shows the feature importance rankings derived from the random forest regression. Line thickness represents feature importance, with thicker lines indicating more important features. Different line types represent positive and negative correlations.

## Discussion

The multilevel meta-analysis showed positive pooled responses for both amendments. SR increased wheat yield, WP, and PFPN by 8.31% (95% CI, 6.93%–9.72%), 8.76% (95% CI, 6.59%–10.99%), and 8.28% (95% CI, 6.86%–9.73%), respectively, whereas BA increased the corresponding outcomes by 10.34% (95% CI, 8.52%–12.20%), 11.48% (95% CI, 8.27%–14.78%), and 9.96% (95% CI, 8.03%–11.44%) (**Table 6**). These point estimates suggest somewhat larger average responses to biochar application, but they should not be interpreted as evidence that biochar was statistically superior because no direct between-amendment contrast was fitted. The consistently positive pooled estimates support the general agronomic value of both practices, whereas the substantial residual heterogeneity (I² = 74.76%–93.53%) shows that the magnitude of benefit is context-dependent. Straw return supplies a readily decomposable carbon and nutrient source and modifies the near-surface hydrothermal environment, whereas biochar is more persistent and may additionally alter porosity, sorption, and nutrient retention (Chen et al., 2022; Han et al., 2023). These mechanistic differences provide plausible explanations for the pooled patterns but do not remove the need to interpret moderator results formally. For both amendment methods, regression analysis indicated a significant positive correlation between yield and WP; furthermore, yield was also positively correlated with PFPN. In addition, the straw return PFPN under SR was positively correlated with WP, but this relationship was not observed under BA (**Figure 3**). The effects of specific variables within these subgroups on yield, WP, and PFPN need to be studied further to optimize the implementation of SR and BA.

### Effects of straw return and biochar application under various climatic conditions on wheat yield, WP, and PFPN

The meta-analysis indicated that climatic conditions significantly affected wheat yield, WP, and PFPN under SR and BA, but the improvements varied with GSP and GST (**Figure 4**). Compared with no straw return, SR resulted in greater increases in wheat yield, WP, and PFPN under GSP < 450 mm than under GSP > 450 mm (**Figures 4a, c, e**), which is consistent with the results of Yu et al. (2018). Several mechanisms may explain this pattern. First, SR improves soil hydrothermal conditions (Zhang et al., 2022a). Second, SR promotes dry matter accumulation and nitrogen translocation to wheat grains (Chen et al., 2017). Third, under relatively dry conditions, surface residues can conserve soil water by reducing non-productive evaporation and increasing soil water availability (Liu et al., 2020). In contrast, under high-rainfall conditions, the marginal water-conservation benefit of SR may diminish, while greater water availability may promote nutrient losses. The random forest results further refine this interpretation. GST ranked third in importance for the response of WP to SR (12.36%), behind soil pH (14.95%) and water supply (14.09%) (**Figure 10**). The increases in wheat yield, WP, and PFPN under SR were generally greater at GST > 15 °C than at GST < 15 °C, probably because warmer conditions accelerate straw decomposition and nutrient mineralization, thereby improving nutrient cycling and crop growth (Guan et al., 2020). Conversely, low temperatures may delay residue decomposition and temporarily reduce nutrient availability; SR can also lower soil temperature during the early growing stage, potentially limiting subsequent yield formation (Wang and Shangguan, 2015). However, excessively high temperatures may inhibit crop respiration and grain filling and thereby constrain the positive effects of SR (Liu et al., 2019; Li et al., 2019). These opposing processes suggest that the climatic response to SR is non-linear and response-specific, operating in conjunction with soil and management conditions rather than indicating a universal climatic threshold.

The increasing effect of BA on wheat yield, WP, and PFPN was observed both in GSP < 450 mm and GSP > 450 mm (**Figures 4b, d, f**). Under relatively dry conditions, the porous structure and high water-holding capacity of biochar can improve soil water retention and reduce non-productive water loss, while its contribution to SOC accumulation may further improve soil conditions for crop growth (Lal, 2009). Under wetter conditions, sufficient water availability may facilitate the beneficial effects of biochar on soil physicochemical properties and nutrient availability, thereby contributing to greater improvements in crop yield and WP (Jeffery et al., 2017). The effects of BA on wheat yield, WP, and PFPN were generally greater at GST > 15 °C (**Figures 4b, d, f**), consistent with previous studies suggesting that warmer conditions may enhance soil biological activity and nutrient cycling, thereby facilitating crop responses to biochar. In regions with mean annual temperatures of 10–20 °C, biochar improved wheat yield and WP by improving soil structure, reducing evaporation, and increasing transpiration, thereby alleviating water stress (Xiao et al., 2024). Moreover, the strong nutrient-retention capacity of biochar, particularly for nitrogen, can reduce nutrient losses and improve nutrient availability, thereby contributing to improvements in both crop productivity and PFPN (Faria et al., 2017; Namatsheve et al., 2024).

### Effects of straw return and biochar application under various soil properties on wheat yield, WP, and PFPN

The responses of wheat yield, WP, and PFPN to SR and BA are also influenced by various soil parameters, such as initial soil texture, SOC, soil BD, and soil pH. Land-use capacity and soil-management practices largely depend on soil texture (Han et al., 2023). Greater yield, WP, and PFPN were observed in coarse soils than in medium and fine soils under SR (**Figures 6a, c, e**), without differences among them, mainly because fine soils with high clay content exhibit enhanced water retention but poor aeration, inhibiting root respiration and ultimately reducing crop yields (Lu et al., 2015). Meanwhile, von Lützow (2006) demonstrated that clay soils inhibited straw decomposition via strong adsorption, reducing nitrogen availability and crop yield, whereas coarse soils maintained better aeration. SR likely enhances crop yield and WP in sandy loam soils by improving soil chemical properties and reducing evaporation (Yu et al., 2018; Moura Chagas et al., 2022). SOC accumulation is a long-term beneficial process for soil health and agricultural sustainability (Carvalhais et al., 2014). The effects on wheat yield, WP, and PFPN were greater at lower SOC and BD levels (**Figures 6a, c, e**). First, carbon-rich straw residues can significantly increase SOC concentration and storage (Malhi et al., 2011). Additionally, SR improved soil aggregate stability and reduced soil BD while simultaneously enhancing PFPN (Zibilske et al., 2002; Karami et al., 2012); moreover, it increased microbial biomass and enhanced the accumulation and activity of soil enzymes (Jiao et al., 2011). We also observed that SR had positive effects on wheat yield, WP, and PFPN in acidic soils at pH < 6.5 (**Figures 6b, d, f**), which was consistent with Moura Chagas et al. (2022). This may be because SR alters soil pH and regulates microbial community composition and activity (Rousk et al., 2010). Moreover, SR with alkaline properties reduced soil acidity and enhanced nutrient retention capacity (Liu et al., 2019). Thus, the effect of appropriate soil acidity and alkalinity under SR on wheat yield, WP, and PFPN requires further study.

Meanwhile, the random forest rankings nevertheless show that initial soil properties have substantial predictive relevance. Soil pH ranked among the three leading variables for five of the six amendment-response combinations; moreover, SOC, BD, and pH were the three leading predictors of the BA yield response (13.81%, 12.13%, and 11.42%, respectively), while pH was the leading predictor of the SR effect on WP (14.95%) (**Figure 10**). This does not conflict with the non-significant Qb tests: subgroup analysis evaluates differences among predefined categories one moderator at a time, whereas Random Forest ranks multivariable predictive contributions and can reflect non-linearities and interactions. Greater effects of BA on yield and WP were observed in coarse soils (**Figures 6b, d**). According to previous studies, coarse soils exhibited greater responsiveness to BA than fine soils (Blanco-Canqui, 2017). In addition, BA reduced water infiltration in coarse soils and enhanced WP. Furthermore, BA enhanced water retention capacity in coarse soils under alternating wetting–drying conditions, thus reducing evaporation rates (Chen et al., 2018). Conversely, Liu et al. (2013) showed that biochar with smaller particle sizes enhanced soil water retention and hydraulic conductivity, leading to short-term yield improvements in fine soils. The effect of BA on PFPN was greater in medium soils (**Figure 6f**), probably because BA reduced soil erosion (Roncák et al., 2023). BA can increase SOC by up to 40% in low-fertility soils and have particularly pronounced effects on yield enhancement (Crane-Droesch et al., 2013; Liu et al., 2016), indicating that BA may have a compensatory effect in nutrient-deficient soils (El-Naggar et al., 2019). This is consistent with our finding that the effects of BA on yield, WP, and PFPN were greater at SOC < 15 g kg^−1^ (**Figures 6b, d, f**). The effects of BA on wheat yield and WP were greater at BD < 1.45 g cm^−3^, whereas the effect on PFPN was greater at BD > 1.45 g cm^−3^. This may be because coarser biochar particles reduced soil BD by compacting soil pores (Chen et al., 2018). Moreover, BA increased soil porosity and ultimately enhanced plant-available water capacity (Blanco-Canqui, 2017). Soil pH directly regulated crop growth by altering microbial populations and nutrient cycling (Dai et al., 2017; Xu et al., 2021). Biochar can increase soil fertility and reduce soil acidity, promoting plant growth (Shetty and Prakash, 2020). BA may also be beneficial for agriculture in low-nutrient and acidic soils (Jeffery et al., 2017). This may explain why BA was more effective at increasing yield and WP at pH < 6.5, whereas its effect on PFPN was greater at soil pH > 6.5 (**Figures 6b, d, f**). Meanwhile, our results demonstrated that BA enhanced PFPN under highly alkaline conditions, which may primarily result from biochar-induced macroaggregate formation, organic matter accumulation, and reduced apparent N loss during cropping (Tang et al., 2023).

### Effects of straw return and biochar application under various agricultural field-management practices on wheat yield, WP, and PFPN

Optimal soil moisture and temperature during SR are critical for maximizing crop yields (Peng et al., 2004; Moura Chagas et al., 2022). In arid and semiarid regions, SR helped meet wheat water demand and improved soil moisture supply stability, while soil moisture availability directly regulated straw decomposition and SOC accumulation (Han et al., 2021; Zhang et al., 2022; Yin et al., 2023). Seasonal drought stress under rainfed systems led to insufficient soil moisture, while SR and irrigation increased water supply and soil organic matter accumulation, promoting sustainable crop production (Gao et al., 2009; Moura Chagas et al., 2022). When water supply exceeded the optimal threshold, no significant differences were observed in wheat yield, WP, and PFPN (Li et al., 2019). This may explain why SR consistently improved yield, WP, and PFPN under lower water supply conditions (**Figures 7a, c, e**). SR combined with N fertilizer is an effective method for optimizing nutrient management in farmland and enhancing grain production capacity (Song et al., 2022). The random forest rankings support this response-specific interpretation: nitrogen application rate was the leading predictor for SR yield (14.40%) and PFPN (18.77%), whereas water supply ranked second for SR WP (14.09%) (**Figure 10**). Our results showed that the effects of SR on yield, WP, and PFPN were greater at NAR < 200 kg ha^−1^ (**Figures 7a, c, e**), which was consistent with Gao et al. (2022), who showed that SR combined with 150 kg ha^−1^ N application was a more effective combination for enhancing PFPN, mainly because SR enriched SOC and total N and enhanced soil carbon and nitrogen sequestration and soil microbial activity (Wu et al., 2024). Additionally, SR reduced nitrate-nitrogen concentrations in soil leachate, effectively reducing nitrogen loss (Guo et al., 2024). Conversely, excessive application of nitrogen fertilizer may reduce yield and hinder sustainable agriculture (Bhatta et al., 2017; Qin et al., 2021). Adjusting SRA according to local conditions may help increase crop yield, WP, and PFPN. For instance, Wang et al. (2017) and Li et al. (2017) showed that an SRA of 2–3 t ha^−1^ increased winter wheat yield in northern regions. Our meta-analysis showed greater effects on yield, WP, and PFPN at SRA < 10 t ha^−1^ (**Figures 9a, c, e**). This may be because low SRA can increase soil nutrients, yield, and PFPN (Qin et al., 2021). Moreover, high SRA led to a decrease in the decomposition rate (Moura Chagas et al., 2022), and microbial decomposition released harmful gases that inhibited root respiration, hindering crop growth (Stagnari et al., 2014). SR promoted wheat yield and WP at straw C/N ratios < 75, whereas PFPN showed a greater response at straw C/N ratios > 75 (**Figures 9a, c, e**), possibly because lower C/N ratios increased net nitrogen mineralization and more strongly stimulated decomposition activity and organic carbon turnover (Liu et al., 2011; Li et al., 2024). Furthermore, SR with a high C/N ratio induced nitrogen fixation and reduced carbon mineralization (Lu et al., 2015). However, high C/N ratios may trigger microbial-crop competition for nitrogen, limiting crop N uptake (Chen et al., 2014). SR is a vital means of soil nutrient protection. A study found three consecutive years of SR increased inorganic N and SOC and reduced resource consumption and the nitrogen application threshold (Chen et al., 2017), indicating that SR may be a low-cost method for increasing wheat yield and PFPN (Guo et al., 2024). This was consistent with our findings (**Figures 9a, c, e**). Moreover, the effect of SR on crop yield decreased over time (**Figure 9a**). The impact of SR duration on crop yields varied with climatic conditions, soil properties, and field management practices (Zhao et al., 2015). Furthermore, long-term SR may lead to saturation of SOC accumulation, which may create favorable environments for pathogens and pests, ultimately resulting in reduced crop yields and soil degradation (Su et al., 2020; Moura Chagas et al., 2022).

BA may improve crop yield, fertilizer use efficiency, and rainwater storage in low-precipitation areas and alleviate soil moisture stress through its porous structure and surface properties under low-precipitation conditions. In areas with limited water resources or in semiarid regions, BA can increase water availability for crops and reduce irrigation frequency of farmland (Blanco-Canqui, 2017; Liu et al., 2025). Another study found that crop yield, WP, and PFPN increased with increasing irrigation amount. This may be attributable to the uncertainty and uneven distribution of rainfall, which may reduce WP; however, excessive rainfall may cause waterlogging, soil erosion, and runoff, thereby reducing soil nutrients and affecting crop production (Liu et al., 2009; Han et al., 2023). Our results showed greater effects of BA on wheat yield, WP, and PFPN at irrigation amounts > 200 mm and GSP < 600 mm (**Figure 7**). N fertilizer plays a key role in modern agricultural production. Kimani et al. (2021) showed that the combination of 150 kg ha^−1^ N fertilizer and 20 t ha^−1^ BAR significantly increased grain nitrogen uptake by 23.9% and PFPN by 34.3%–246.9%. However, excessive nitrogen can inhibit nitrogen and phosphorus uptake by crops, restrict photosynthesis, and diminish partial factor productivity of nitrogen (Yan et al., 2020). This may explain why the effects of BA on yield, WP, and PFPN were greater at NAR < 300 kg ha^−1^ than at NAR > 300 kg ha^−1^ (**Figures 7b, d, e**). Previous research has indicated that rice yield improves when BAR exceeds 20 t ha^−1^ (Liu et al., 2022). Meanwhile, low BAR did not affect available soil water content (Glab et al., 2016); notably, higher BAR significantly increased available soil water (Xiao et al., 2016). Our results indicated that BAR > 20 t ha^−1^ had greater effects on wheat yield, WP, and PFPN (**Figures 9b, d, e**). An appropriate dose of BA was the key to success (Dong et al., 2019; Sun et al., 2019). The biochar C/N ratio is a key parameter that affects biochar’s soil-improvement effects and nitrogen dynamics. Our analysis showed that BA enhanced wheat yield and WP in biochar C/N < 75, while PFPN showed better results at biochar C/N > 75 (**Figures 9b, d, f**), probably because low C/N ratio biochar was commonly employed in microbial-mediated processes and suppressed the SOC mineralization (Singh and Cowie, 2014). Furthermore, high C/N biochar may reduce N availability and consequently restrict crop growth (Jeffery et al., 2017). However, the application of high-C/N-ratio biochar can trigger microbial N immobilization and facilitate SOC sequestration, thereby improving PFPN through optimized N cycling dynamics (Cayuela et al., 2010; Dai et al., 2020). Short-term biochar application under identical conditions significantly outperformed medium- to long-term application in enhancing maize yield, water productivity, and PFPN, probably because the positive effect of BA on crop yield enhancement was relatively short-lived, likely because of the progressive loss of biochar-derived carbon over time (Wang et al., 2016; Xiao et al., 2024; Wang et al., 2024). BA benefits declined over time after application (Blanco-Canqui et al., 2024). Thus, short-term application of biochar at a moderate rate represented a favorable application strategy (Xiao et al., 2024). More importantly, a systematic understanding of biochar characteristics is essential for developing guidelines for its optimal application in sustainable agriculture. Higher application rates may provide greater capacity to improve soil porosity, water retention, and nutrient retention, but these benefits remain context-dependent (Jeffery et al., 2017; Xiao et al., 2016; Dong et al., 2019). This context dependence is also evident in the random forest results: water supply was the leading predictor of BA-induced WP (14.10%), while nitrogen application rate (21.00%) and irrigation amount (10.00%) were the two leading predictors of BA-induced PFPN; in contrast, SOC, BD, and pH were more influential for BA yield (**Figure 10**). Collectively, the subgroup and random forest analyses show that field management affects different outcomes through distinct pathways and should be optimized jointly with soil conditions rather than interpreted through isolated subgroup maxima.

## Conclusion

This global synthesis demonstrates that SR and BA can simultaneously improve wheat productivity and resource-use efficiency. SR increased yield, WP, and PFPN by 8.31%, 8.76%, and 8.28%, respectively, while BA increased them by 10.34%, 11.48%, and 9.96%. The magnitude of these benefits was closely associated with soil properties and field-management practices. SR produced stronger yield and PFPN responses under lower nitrogen inputs, whereas the yield and PFPN responses to BA increased progressively with biochar application rate. Water supply was also an important predictor of WP under both amendments. Accordingly, nitrogen management with BA should be determined according to local soil fertility and water availability rather than using a single general rate. The Random Forest rankings further highlighted the importance of coordinating amendment application with soil, water, and nutrient management. Locally adapted SR and BA strategies can therefore support sustainable wheat production while improving the efficiency of water and nitrogen resources.

## Data Availability

The original contributions presented in the study are included in the article/**Supplementary Material**. Further inquiries can be directed to the corresponding authors.
