# Supplementary material for: The response of wheat yield, water productivity, and partial factor productivity of nitrogen to straw return and biochar application: a global meta-analysis
Source: Front Plant Sci. 2026 Sep 30;17:1923733. doi: 10.3389/fpls.2026.1923733 (PMC13693579; doi:10.3389/fpls.2026.1923733)
Supplement: Supplementary file 1 [file DataSheet1.pdf]

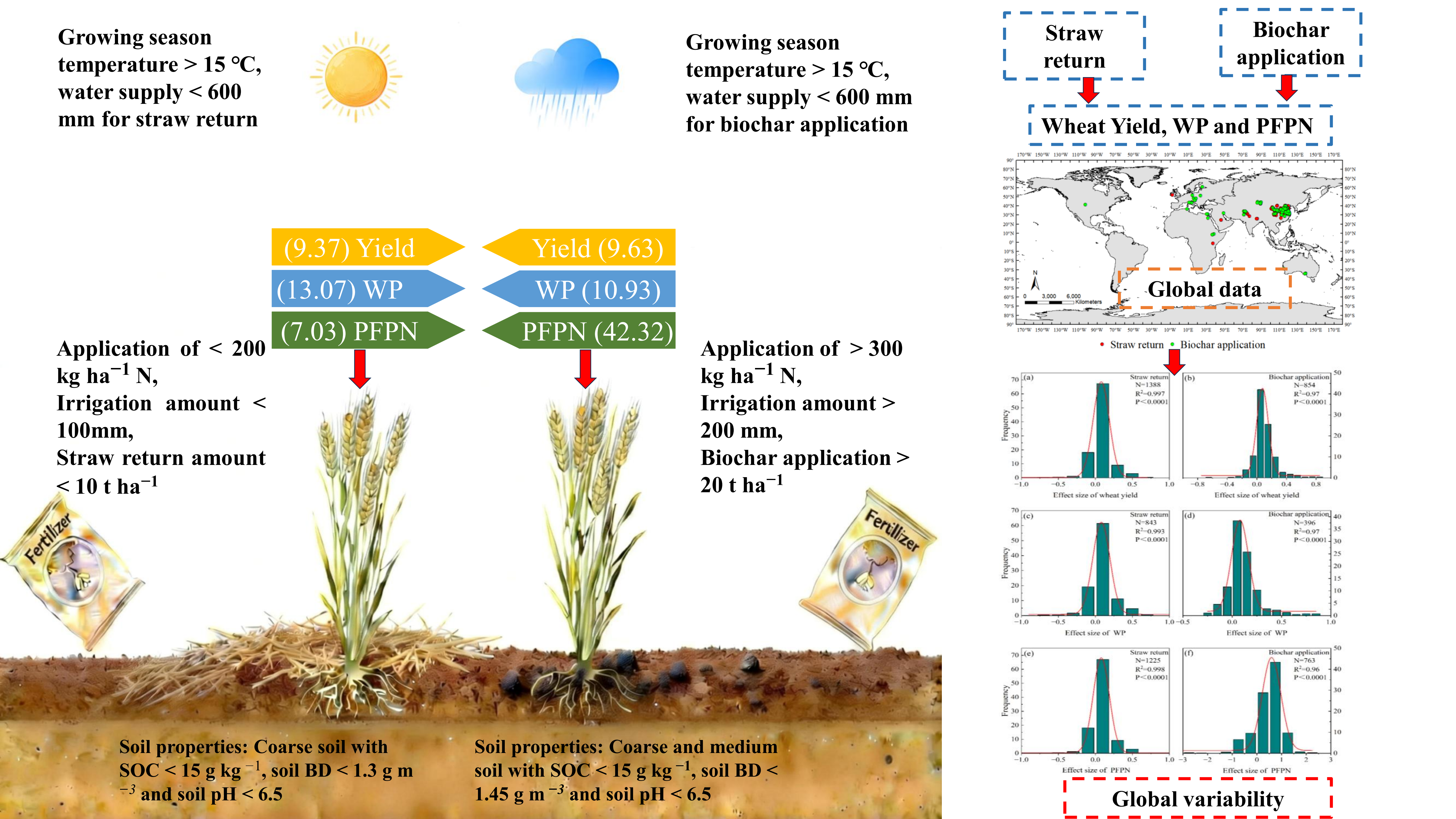

Growing season temperature > 15 °C, water supply < 600 mm for straw return
Growing season temperature > 15 °C, water supply < 600 mm for biochar application
Straw return
Biochar application
Wheat Yield, WP and PFPN
Global data
Straw return
Biochar application
(9.37) Yield
(13.07) WP
(7.03) PFPN
Yield (9.63)
WP (10.93)
PFPN (42.32)
Application of < 200 kg ha^{-1} N,
Irrigation amount < 100mm,
Straw return amount < 10 t ha^{-1}
Application of > 300 kg ha^{-1} N,
Irrigation amount > 200 mm,
Biochar application > 20 t ha^{-1}
Fertilizer
Fertilizer
Soil properties: Coarse soil with SOC < 15 g kg^{-1}, soil BD < 1.3 g m^{-3} and soil pH < 6.5
Soil properties: Coarse and medium soil with SOC < 15 g kg^{-1}, soil BD < 1.45 g m^{-3} and soil pH < 6.5
(a) Straw return N=1388 R^2=0.997 P<0.0001
(b) Biochar application N=854 R^2=0.97 P<0.0001
Effect size of wheat yield
(c) Straw return N=843 R^2=0.993 P<0.0001
(d) Biochar application N=396 R^2=0.97 P<0.0001
Effect size of WP
(e) Straw return N=1225 R^2=0.998 P<0.0001
(f) Biochar application N=763 R^2=0.96 P<0.0001
Effect size of PFPN
Frequency
Global variability
