# Supplementary material for: The response of wheat yield, water productivity, and partial factor productivity of nitrogen to straw return and biochar application: a global meta-analysis
Source: Front Plant Sci. 2026 Sep 30;17:1923733. doi: 10.3389/fpls.2026.1923733 (PMC13693579; doi:10.3389/fpls.2026.1923733)
Supplement: Supplementary file 2 [file DataSheet2.docx]

**Table 1.** Effect size of SR and BA on wheat yield, WP and PFPN.

| Index | Treatments | Effect size | Numbers | Fail-Safe Number |
| --- | --- | --- | --- | --- |
| Wheat Yield | SR  BA | 8.31(6.93~9.72)  10.34 (8.52~12.2) | 1388  854 | 1928432.9  1512891 |
| Wheat WP | SR  BA | 8.76 (6.59~10.99)  11.48 (8.27~14.78) | 843  396 | 608373.3  204071.4 |
| Wheat PFPN | SR  BA | 8.28 (6.86~9.73)  9.96 (8.03~11.44) | 1225  763 | 788394.8  326580.1 |

*Note:* SR, straw return; BA, biochar application; WP, water productivity; PFPN, partial factor productivity of nitrogen.

**Table 2.** Overall random-effects estimates and heterogeneity statistics for straw return and biochar application.

| SR/BA | Y/wheat | N | | Pooled change  (95% CI) | Q (df) | P_Q_ | I² (%) | τ² |
| --- | --- | --- | --- | --- | --- | --- | --- | --- |
| Straw return | Yield | | 1388 | 8.31(6.93~9.72) | 17,499.05 (1,387) | <0.001 | 92.07 | 0.01632 |
| Straw return | WP | | 843 | 8.76 (6.59~10.99) | 13,014.21 (842) | <0.001 | 93.53 | 0.02435 |
| Straw return | PFPN | | 1225 | 8.28 (6.86~9.73) | 9,405.75 (1,224) | <0.001 | 86.99 | 0.01683 |
| Biochar application | Yield | | 854 | 10.34 (8.52~12.2) | 10,121.49 (853) | <0.001 | 91.57 | 0.01719 |
| Biochar application | WP | | 396 | 11.48 (8.27~14.78) | 2,913.36 (395) | <0.001 | 86.44 | 0.01944 |
| Biochar application | PFPN | | 763 | 9.96 (8.03~11.44) | 3,019.18 (762) | <0.001 | 74.76 | 0.01707 |

*Note:* Effect sizes were calculated as lnRR = ln(ME/MC), where ME and MC are the experimental- and control-group means, respectively. The pooled percentage change was back-transformed as [exp(pooled lnRR) − 1] × 100. Sampling variance was calculated as SDE²/(NE × ME²) + SDC²/(NC × MC²). Between-comparison variance (τ²) was estimated by restricted maximum likelihood (REML), and pooled effects were estimated using a random-effects model. WP, water productivity; PFPN, partial factor productivity of nitrogen; n, number of treatment–control comparisons.

**Table 3.** Egger's regression and Begg's rank-correlation tests for small-study effects

| Amendment | Response | Egger's regression | | | Begg's test | | |
| --- | --- | --- | --- | --- | --- | --- | --- |
|  |  | Intercept | z | p | Kendall's tau | z | p |
| Straw return | Yield | -0.445 | -2.676 | 0.007 | 0.018 | 0.449 | 0.653 |
| Straw return | WP | 0.032 | 0.080 | 0.936 | -0.044 | -0.846 | 0.397 |
| Straw return | PFPN | -1.238 | -6.653 | <0.001 | 0.031 | 0.730 | 0.466 |
| Biochar application | Yield | -0.248 | -0.939 | 0.348 | 0.002 | 0.028 | 0.977 |
| Biochar application | WP | 0.306 | 0.638 | 0.523 | -0.110 | -1.269 | 0.204 |
| Biochar application | PFPN | -3.224 | -2.129 | 0.033 | -0.119 | -2.054 | 0.040 |

*Notes:* Egger's test evaluates whether the regression intercept differs from zero. Begg's test is based on Kendall's rank correlation. Bold p values indicate p < 0.05. Statistical asymmetry indicates possible small-study effects and does not, by itself, prove publication bias. Egger’s test indicated funnel-plot asymmetry for straw return yield (p = 0.007), straw return PFPN (p < 0.001), and biochar application PFPN (p = 0.033). Begg’s test was significant only for biochar application PFPN (p = 0.040), whereas neither test indicated asymmetry for the other three outcomes. The trim-and-fill procedure imputed 17, 19, and 10 studies for straw return yield, straw return PFPN, and biochar application PFPN, respectively. Their pooled RRs changed only moderately from 1.083 to 1.097, 1.084 to 1.101, and 1.097 to 1.115. Because the imputed studies were located on the right side of the funnel plots, the adjustment increased rather than reduced the effects and did not show the conventional pattern of missing small negative studies.

**Table 4.** Effect sizes before and after the trim-and-fill sensitivity analysis

| Amendment | Response | Original dataset | | | | Filled studies | Trim-and-fill adjusted dataset | | | |
| --- | --- | --- | --- | --- | --- | --- | --- | --- | --- | --- |
|  |  | p | RR | 95% CI | I² (%) |  | p | RR | 95% CI | I² (%) |
| Straw return | Yield | <0.001 | 1.083 | [1.069, 1.097] | 78.50 | 17 | <0.001 | 1.097 | [1.082, 1.112] | 81.27 |
| Straw return | WP | <0.001 | 1.083 | [1.061, 1.105] | 87.52 | 0 | <0.001 | 1.083 | [1.061, 1.105] | 87.52 |
| Straw return | PFPN | <0.001 | 1.084 | [1.069, 1.098] | 65.81 | 19 | <0.001 | 1.101 | [1.085, 1.117] | 70.80 |
| Biochar application | Yield | <0.001 | 1.101 | [1.083, 1.118] | 83.40 | 0 | <0.001 | 1.101 | [1.083, 1.118] | 83.40 |
| Biochar application | WP | <0.001 | 1.112 | [1.080, 1.144] | 65.13 | 0 | <0.001 | 1.112 | [1.080, 1.144] | 65.13 |
| Biochar application | PFPN | <0.001 | 1.097 | [1.077, 1.117] | 53.83 | 10 | <0.001 | 1.115 | [1.093, 1.137] | 62.94 |

*Notes:* RR, response ratio (exp[lnRR]); CI, confidence interval; I², percentage of total variability attributable to heterogeneity. Filled studies are the number of studies imputed by the Duval-Tweedie trim-and-fill procedure. Trim-and-fill was applied only when Egger's or Begg's test indicated asymmetry (p < 0.05); when no asymmetry was detected, no studies were filled and the adjusted estimate equals the original estimate. Analyses were conducted at the publication level after aggregating correlated comparisons within publications using an assumed within-publication correlation of rho = 0.8.

The trim-and-fill analysis imputed 17, 19, and 10 potentially missing studies for straw return yield, straw return PFPN, and biochar application PFPN, respectively. The corresponding pooled RRs increased moderately from 1.083 to 1.097, from 1.084 to 1.101, and from 1.097 to 1.115. No missing studies were imputed for the other three outcomes, and their pooled estimates remained unchanged. All adjusted confidence intervals remained above 1.0 (p < 0.001), indicating that the directions and statistical significance of the overall effects were robust to the trim-and-fill sensitivity analysis. However, heterogeneity remained substantial and the imputed studies occurred on the right side of the funnel plots; therefore, the adjusted estimates should be regarded as sensitivity results rather than definitive bias-corrected effects.

**Table 5. Between-subgroup heterogeneity tests for straw return/SR**

| **Table 5. Between-subgroup heterogeneity tests for straw return/SR** | | | | | | | |
| --- | --- | --- | --- | --- | --- | --- | --- |
| **Factor class** | **Explanatory variable** | **Yield** | | **WP** | | **PFPN** | |
|  |  | **Qb** | **P value** | **Qb** | **P value** | **Qb** | **P value** |
| **Climate factors** | Growing-season precipitation | 4.33 | 0.228 | 5.09 | 0.165 | 2.14 | 0.543 |
|  | Growing-season temperature | 1.79 | 0.408 | 0.86 | 0.652 | 1.91 | 0.385 |
| **Soil physicochemical factors** | Soil texture | 4.37 | 0.112 | 2.41 | 0.299 | 3.06 | 0.217 |
|  | Soil organic carbon | 2.14 | 0.344 | 3.88 | 0.144 | 2.42 | 0.298 |
|  | Soil bulk density | 5.16 | 0.076 | 5.62 | 0.060 | 4.99 | 0.082 |
|  | Soil pH | 2.05 | 0.561 | 2.60 | 0.457 | 1.79 | 0.618 |
| **Field managements** | Water supply | 2.63 | 0.452 | 1.53 | 0.675 | 8.27 | 0.041 |
|  | Irrigation amount | 0.01 | 0.996 | 0.83 | 0.659 | 0.19 | 0.908 |
|  | Nitrogen application rate | 10.12 | 0.018 | 4.21 | 0.239 | 9.27 | 0.026 |
|  | Straw application rate | 0.77 | 0.679 | 0.40 | 0.820 | 2.42 | 0.298 |
|  | Straw C/N ratio | 2.94 | 0.229 | 1.31 | 0.518 | 1.94 | 0.379 |
|  | Experimental duration | 0.43 | 0.806 | 1.18 | 0.554 | 0.04 | 0.980 |
|  | | | | | | | |

**Table 6** Between-subgroup heterogeneity tests for biochar application/BA

| **Table 6. Between-subgroup heterogeneity tests for biochar application/BA** | | | | | | | |
| --- | --- | --- | --- | --- | --- | --- | --- |
| **Factor class** | **Explanatory variable** | **Yield** | | **WP** | | **PFPN** | |
|  |  | **Qb** | **P value** | **Qb** | **P value** | **Qb** | **P value** |
| **Climate factors** | Growing-season precipitation | 2.74 | 0.433 | 3.21 | 0.361 | 2.98 | 0.394 |
| **Climate factors** | Growing-season temperature | 3.78 | 0.151 | 5.22 | 0.074 | 4.81 | 0.090 |
| **Soil physicochemical factors** | Soil texture | 5.74 | 0.057 | 1.55 | 0.460 | 4.91 | 0.086 |
| **Soil physicochemical factors** | Soil organic carbon | 0.49 | 0.784 | 0.23 | 0.892 | 0.23 | 0.893 |
| **Soil physicochemical factors** | Soil bulk density | 0.64 | 0.726 | 0.70 | 0.706 | 0.25 | 0.881 |
| **Soil physicochemical factors** | Soil pH | 1.23 | 0.746 | 1.96 | 0.581 | 2.14 | 0.544 |
| **Water and nitrogen management** | Water supply | 0.77 | 0.858 | 3.22 | 0.359 | 0.46 | 0.927 |
| **Water and nitrogen management** | Irrigation amount | 1.24 | 0.538 | 1.65 | 0.439 | 2.80 | 0.246 |
| **Water and nitrogen management** | Nitrogen application rate | 1.26 | 0.738 | 1.83 | 0.609 | 1.62 | 0.655 |
| **Amendment management** | Biochar application rate | 17.77 | 0.001 | 8.22 | 0.084 | 17.92 | 0.001 |
| **Amendment management** | Biochar C/N ratio | 0.30 | 0.862 | 0.38 | 0.827 | 0.77 | 0.682 |
| **Amendment management** | Experimental duration | 0.11 | 0.947 | 3.14 | 0.208 | 0.01 | 0.997 |

**Figure S1.** PRISMA flowchart illustrating paper identification, screening and selection (n = Number of publications)

**Figure S2.** Funnel plot of study size against the effect of Straw return and Biochar application on wheat yield, Water productivity and the partial factor productivity of nitrogen. Yield, WP and PFPN represent wheat yield, Water productivity and the partial factor productivity of nitrogen.

**Figure S3.** Leave-one-publication-out sensitivity analysis of the effects of straw return and biochar application on wheat yield, water productivity (WP) and partial factor productivity of nitrogen (PFPN). Publications were identified using normalized Title values. Each point shows the pooled percentage effect after omitting one publication and refitting the multilevel random-effects model; shaded regions show the corresponding 95% confidence intervals. Solid black lines indicate full-model estimates and dashed grey lines indicate their 95% confidence intervals. Open circles mark the minimum and maximum leave-one-out estimates.

Leave-one-publication-out sensitivity analyses showed that the pooled effects were not driven by any single Title-defined publication. Across all publication omissions, the pooled increases ranged from 8.17% to 8.54% for straw-return yield, 8.45% to 9.12% for straw-return WP, 8.12% to 8.44% for straw-return PFPN, 9.88% to 10.53% for biochar-application yield, 10.55% to 11.88% for biochar-application WP, and 9.51% to 10.19% for biochar-application PFPN. All corresponding 95% confidence intervals remained above zero, and all model refits converged. The largest change was 0.93 percentage points for biochar-application WP, indicating that the overall conclusions were stable to the omission of individual publications.
