# Supplementary material for: The response of wheat yield, water productivity, and partial factor productivity of nitrogen to straw return and biochar application: a global meta-analysis
Source: Front Plant Sci. 2026 Sep 30;17:1923733. doi: 10.3389/fpls.2026.1923733 (PMC13693579; doi:10.3389/fpls.2026.1923733)
Supplement: Supplementary file 3 [file SupplementaryFile1.docx]

**A.1 A list of 247 papers from which the data were extracted for wheat yield, WP and PFPN of straw return (SR).**

1 Li, C.X., Zhang, L.L., Ma, S.C., Shao, Y., Chen, H.T., Wang, J.R., 2017. Effects of Organic Materials Returning on Soil Carbon and Nitrogen Contents, Yield and Economic Benefit in Wheat. Crops. 2, 145–150. [https://doi.org/10.16035/j.issn.1001-7283.2017.02.026.](%20https:/doi.org/10.16035/j.issn.1001-7283.2017.02.026.) (in Chinese with English abstract).
2 Tan, D.S., Jin, J.Y., Huang, S.W., Gao, W., 2008. Effect of long-term application of potassium fertilizer and wheat straw to soil on yield of crops and soil potassium in fluvo-aquic soil and brown soil of north-central China. J. Plant Nutr. Fert. 1, 106–112. [https://doi.org/ 10.11674/zwyf.2008.0117](https://doi.org/%2010.11674/zwyf.2008.0117). (in Chinese with English abstract).
3 Yang, X.L., Lu, Y.L., Tong, Y.A., Lin, W., Liang, T., 2013. Effects of long-term N application and straw returning on N budget under wheat-maize rotation system. J. Plant Nutr. Fert. 19(1), 65–73. https://doi.org/ 10.11674／zwyf.2013.0108. (in Chinese with English abstract).
4 Wang, L., Qian, X., Gao, Y.B., Zhang, H., Liu, K.C., Chen, G.Q., Li, Z.X., 2023. Optimizing straw management to enhance carbon and nitrogen efficiency and economic benefit of wheat-maize double cropping system. J. Environ. Sci. 44(1), 463–472. https://doi.org/10.13227/j.hjkx.202203005. (in Chinese with English abstract).
5 Gai, X.P., Liu, H.B., Zhai, L.M., Yang, B., Ren, T.Z., Wang, H.Y., Wu, S.X., Lei, Q.L., 2018. Effects of long-term additional application of organic manure or straw incorporation on soil nitrogen leaching risk. Sci. Agric. Sin. 51(12), 2336–2347. https://doi.org/10.3864/j.issn.0578-1752.2018.12.010. (in Chinese with English abstract).
6 Li, H.Y., Meng, Z.L., Pang, D.W., Chen, J., Hou, Y.K., Cui, H.X., Jin, M., Wang, Z.L., Li, Y., 2019. Effect of annual straw return model on soil carbon sequestration and crop yields in winter wheat-summer maize rotation farmland. Acta Agron. Sin. 45(6), 893–903. https://doi.org/10.3724/SP.J.1006.2019.81078. (in Chinese with English abstract).
7 Zhou, Z.J., Guo, S., Chen, K., Zeng, X.Z., Shangguan, Y.X., Yu, H., Qin, Y.S., Tu, S.H., He, M.J., Li, L.J., 2021. Long-term straw mulching affects rice and wheat yields, soil nitrogen fractions, and microbial community under a no-till system. Acta Pedologica Sinica. 59(4), 1148–1159. https://doi.org/10.11766/trxb202011190522. (in Chinese with English abstract).
8 Zhang, S.Q., Zhang, B., Yue, K., Lin, S., Guo, D.D., Guo, T.F., Huang, S.M., 2022. Effects of long-term fertilization on the nitrogen absorption and ammonia volatilization of wheat in the Huang-Huai-Hai plain of China. Soil Fert. Sci. China. (11), 40–47. https://doi.org/10.11838/sfsc.1673-6257.21560. (in Chinese with English abstract).
9 Liu, X.T., Cao, C.Y., Zheng, C.L., Dang, H.K., Ma, J.Y., Li, K.J., 2023. Effects of long-term straw return on carbon and nitrogen contents of aggregates and crop yield in fluvo-aquic soil. Soil Fert. Sci. China. 4, 25–33. https://doi.org/10.11838/sfsc.1673-6257.22139. (in Chinese with English abstract).
10 Xu, Y., Jiang, L.H., Sun, Z., Shi, J., Wei, J.L., Wang, M., Li, G.S., Liu, Z.H., 2015. Effects of corn straw returning and nitrogen fertilizer application methods on N2O emission from wheat growing season. J. Agric. Resour. Environ. 32(6), 552–558. https://doi.org/10.13254/j.jare.2015.0088. (in Chinese with English abstract).
11 Lu, J.J., Wang, Y.H., Li, J.H., Ding, Z.Q., Yao, Y.Q., Zhang, J., 2008. Effects of different mulching methods on winter wheat growth on West of Henan dry-land. Agricultural Research in the Arid Areas. 2, 94–97. https://doi.org/10.1145/1344411.1344416. (in Chinese with English abstract).
12 Luo, Z.Z., Cai, L.Q., Li, L.L., Niu, Y.N., Zhang, R.Z., Xie, J.H., 2015. Long-term effects of tillage system on soil nutrients and grain yields in rainfed area of Loess Plateau. Agricultural Research in the Arid Areas. 33(3), 171–176. https://doi.org/10.7606/j.issn.1000-7601.2015.03.28. (in Chinese with English abstract).
13 Ma, S.K., Huo, K., Zhang, D.X., Zhang, J., Zhang, J.H., Chai, X.R., Wang, H.Z., 2023. Effects of maize straw return combined with nitrogen on soil enzyme activity and nitrogen fertilizer use efficiency in western dryland wheat fields of Henan Province. Acta Prataculturae Sinica. 32(6), 120–133. https://doi.org/10.11686/cyxb2022269. (in Chinese with English abstract).
14 Jin, Y.Q., Du, B.J., Gao, H.J., Chang, J., Zhang, L.G., 2013. Effects of maize straw returning on water dynamics and water use efficiency of winter wheat in lime concretion black soil. J. Triticeae Crops. 33(1), 89–95. https://doi.org/[10.7606/j.issn.1009-1041.2013.01.016](https://doi.org/10.7606/j.issn.1009-1041.2013.01.016). (in Chinese with English abstract).
15 Xing. S.L., Han, B.W., Liu, M.C., Xu, M.G., 2010. The effect of NPK fertilizer combined with soil organic manure on soil nutrition and wheat yield increasing. J. Agro-Environ. Sci. 29(S1), 135–140. (in Chinese with English abstract).
16 Zhang, S.Y., Wang, H.Z., Yang, M.D., Wang, J.L., He, D.X., 2016. Influence of returning corn stalks to field under different soil moisture contents on root growth and water use efficiency of wheat (Triticum aestivum L.). Sci. Agric. Sin. 49(13), 2484–2496. https://doi.org/10.3864/j.issn.0578-1752.2016.13.004. (in Chinese with English abstract).
17 Luo, C.Y., Shen, Y.Y., Nan, Z.B., Gao, C.Y., Chan, Y., Fan, L.Q., 2005. Dynamics of crop yield and soil oxidizable organic carbon fraction within a maize-winter-soy rotation under different tillage treatment in Longdong Loess Plateau. J. Soil Water Conserv. 4, 84–88. https://doi.org/[10.3321/j.issn:1009-2242.2005.04.020](https://doi.org/10.3321/j.issn:1009-2242.2005.04.020). (in Chinese with English abstract).
18 Wan, Y.F., Li, Y.E., Gao, Q.Z., Qin, X.B., Lin, E.D., 2009. Field managements affect yield, soil carbon, and greenhouse gases emission of winter wheat in North China Plain. J. Agro-Environ. Sci. 28(12), 2495–2500. https://doi.org/[10.3321/j.issn:1672-2043.2009.12.009](https://doi.org/10.3321/j.issn:1672-2043.2009.12.009). (in Chinese with English abstract).
19 Zhao, Y., Zhou, F., Luo, J.L., Zhao, Y.H., Wang, N., Yu, J.G., Xue, L.H., Yang, L.Z., 2021. Effects of rice straw returning combined with fertilizer application on yield and nitrogen utilization of wheat. Soils. 53(5), 937–944. https://doi.org/10.13758/j.cnki.tr.2021.05.007. (in Chinese with English abstract).
20 Tan, D.S., Jin, J.Y., Huang, S.W., Chen, Z.Q., 2009. Study on effect of long-term application of K fertilizer and returning straw to soil on soil nutrient and wheat yield in the region of castanozem. Agricultural Research in the Arid Areas. 27(2), 194–198. https://doi.org/CNKI:SUN:GHDQ.0.2009-02-036. (in Chinese with English abstract).
21 Sun, X.X., Chang, Z.Z., Jin, H.M., Shen, M.X., Lu, C.Y., Wang, H.H., 2017. Influence of different ways of straw incorporation on crop yield and economic benefit in the Taihu Lake Basin. Jiangsu Agric Sci. 33(1), 94–99. https://doi.org/[10.3969/j.issn.1000-4440.2017.01.015](https://doi.org/10.3969/j.issn.1000-4440.2017.01.015). (in Chinese with English abstract).
22 Li, F.C., Zhao, H.B., Wang, Z.H., Xue, C., Dai, J., Meng, X.Y., Gao, Y.J., 2011. Effects of straw mulching and planting green manure on soil water, nutrient and winter wheat yield on Weibei Plateau, China. J. Agro-Environ. Sci. 30(9), 1861–1871. https://doi.org/CNKI:SUN:NHBH.0.2011-09-032. (in Chinese with English abstract).
23 Hong, A.M., Duan, Y.H., Zhang, H.Y., Han, M., Li, Y., Shen, X., Sun, G.J., Shen, J.H., Ran, W., Zhang, M., 2021. Impacts of composted pig manure and straw amendments on the growth of Beckmannia syzigachne in wheat field of a rice-wheat rotation system. Chin. J. Ecol. 40(12), 3944–3951. https://doi.org/10.13292/j.1000-4890.202112.033. (in Chinese with English abstract).
24 Wu, J.S., Xu, M.M., Wang, X.H., Yang, H.S., Liu, X.F., Dai, Y.J., Bian, X.M., 2015. Effects of ditch-buried rice straw on soil organic matter, enzyme activities and yield. Chin. J. Soil Sci. 46(1), 203–209. https://doi.org/10.19336/j.cnki.trtb.2015.01.034. (in Chinese with English abstract).
25 Hang, Y.H., Wang, Q.S., Xu, G.C., Liu, X., 2017. Effects of water regimes and straw incorporation on greenhouse gas emissions in a rice-wheat cropping system. J. Ecol. Environ. Sci. 26(11), 1844–1855. https://doi.org/10.16258/j.cnki.1674-5906.2017.11.004. (in Chinese with English abstract).
26 Guo, J.H., Li, M.Y., Zhang, J., Li, S., Peng, Y.M., Xiao, H.S., Tian, W.Z., Li, J.H., Zhang, J., Li, F., Huang, M., Li, Y.J., 2024. Effects of organic fertilizer application and straw return on crop yield, quality, and fertilizer efficiency in summer maize-winter wheat double cropping system. Agricultural Research in the Arid Areas. 42(3), 118–126. https://doi.org/10.7606/j.issn.1000-7601.2024.03.13. (in Chinese with English abstract).
27 Wu, J., Guo, X.S., Lu, J.W., Wang, Y.Q., Xu, Z.Y., Zhang, X.L., 2012. Effects of continuous straw mulching on soil physical and chemical properties and crop yields in paddy-upland rotation system. J. Plant Nutr. Fert. 18(3), 587–594. https://doi.org/10.11674/zwyf.2012.11304. (in Chinese with English abstract).
28 Li, H.X., Gao, F., Ren, B.Z., Zhao, B., Liu, P., Zhang, J.W., 2022. Effects of straw incorporation and nitrogen application rate on winter wheat yield and nitrogen utilization. J. Plant Nutr. Fert. 28(7), 1260–1270. https://doi.org/10.11674/zwyf.2021606. (in Chinese with English abstract).
29 Zhang, Y.L., Lü, J.L., Jin, J.Y., Li, S.T., Chen, Z.Q., Gao, X.S., 2012. Effects of chemical fertilizer and straw return on soil fertility and spring wheat quality. Plant Nutr. Fert. Sci. 18(2), 307–314. https://doi.org/10.11674/zwyf.2012.11214. (in Chinese with English abstract).
30 Han, B., Li, Z.J., Wang, Y., Ning, T.Y., Zheng, Y.H., 2007. Effects of soil tillage and returning straw to soil on wheat growth status and yield. Nongye Gongcheng Xuebao. 2, 48–53. https://doi.org/[10.3321/j.issn:1002-6819.2007.02.010](https://doi.org/10.3321/j.issn:1002-6819.2007.02.010). (in Chinese with English abstract).
31 Liu, Y., Liu, W.L., Zhu, B., 2016. Effect of fertilization regime on soil N2O emission from upland field under wheat-maize rotation system. Acta Pedologica Sinica. 53(3), 735–745. https://doi.org/10.11766/trxb201510080392. (in Chinese with English abstract).
32 Yang, L.R., Lü, N., Zhang, Z., Liu, A.D., Ma, X.F., Yang, G.Z., 2023. Decomposition characteristics of cotton stalk and its impact on yield of wheat. J. Huazhong Agric. Univ. 42(4), 132–139. https://doi.org/10.13300/j.cnki.hnlkxb.2023.04.015. (in Chinese with English abstract).
33 Wang, W.Y., Qiao, B., Akhtar, K., Yuan, S., Ren, G.X., Feng, Y.Z., 2016. Effects of straw returning to field on soil respiration and soil water heat in winter wheat-summer maize rotation system under no tillage. Sci. Agric. Sin. 49(11), 2136–2152. https://doi.org/10.3864/j.issn.0578-1752.2016.11.01. (in Chinese with English abstract).
34 Zhao. H.X., Chi. S.Y., Ning. T.Y., Tian. S.Z., Wang. B.W., Li. Z.J., 2013.Covering farming pattern to improve soil physical properties and crop yield in wheat-maize cropping system. Nongye Gongcheng Xuebao. 29(09),113–122. https://doi.org/10.3969/j.issn.1002-6819.2013.09.015. (in Chinese with English abstract).
35 Zhang, L., Wang, J., Pang, H.C., Zhang, J.T., Guo, J.J., Dong, G.H., Cong, P., 2017. Effect of granulated straw incorporation on soil nutrient and grain yield of winter wheat. Chin. J. Eco-Agric. 25(12), 1770–1778. https://doi.org/10.13930/j.cnki.cjea.170451. (in Chinese with English abstract).
36 Han, S., Wu, J., Li, M., Chen, F., Wang, Y.Q., Cheng, W.L., Tang, S., Wang, H., Guo, X.S., Lu, C.A., 2020. Deep tillage with straw returning increase crop yield and improve soil physicochemical properties under topsoil thinning treatment. J. Plant Nutr. Fert. 26(2), 276–284. https://doi.org/10.11674/zwyf.19154. (in Chinese with English abstract).
37 Lu, Q., Wang, J.C., Li, J., Wang, L., Zhang, L., Halihashi,·Y., Wang, Q.J., Zhang, J.C., Huang, Q.W., Shen, Q.R., 2014. Effect of straw returning and combined applications of organic fertilizer and inorganic fertilizer on grain yield and nitrogen utilization under rice-wheat rotation system. J. Nanjing Agric. Univ. 37(6), 66–74. https://doi.org/[10.7685/j.issn.1000-2030.2014.06.010](https://doi.org/10.7685/j.issn.1000-2030.2014.06.010). (in Chinese with English abstract).
38 Wu, X.L., Tang, Y.L., Li, C.S., Wu, C., Huang, G., 2015. Effects of autumn straw mulching on physiological characteristics and water use efficiency in winter wheat grown in hilly drought region. Acta Agron. Sin. 41(6), 929–937. https://doi.org/10.3724/sp.J.1006.2015.00929. (in Chinese with English abstract).
39 Guo, W., Li, D.D., Xu, J.S., Zhou, Y.P., Wang, Q.X., Zhou, T.T., Zhao, B.Z., 2024. Effects of application of straw and organic-inorganic fertilizers on soil quality and wheat yield in different texture fluvo-aquic soils. Acta Pedologica Sinica. 61(5), 1360–1373. https://doi.org/10.11766/trxb202306120228. (in Chinese with English abstract).
40 Zhang, S., Shi, Z.L., Yang, S.J., Gu, K.J., Dai, T.B., Wang, F., Li, X., Sun, R.H., 2015. Effects of nitrogen application rates and straw returning on nutrient balance and grain yield of late sowing wheat in rice-wheat rotation. Chin. J. Appl. Ecol. 26(9), 2714–2720. https://doi.org/10.13287/j.1001-9332.20150630.009. (in Chinese with English abstract).
41 Sun, H.N., Wang, S.W., Li, Y.L., Yang, W.J., Yin, X.S., Yin, L.N., Deng, X.P., 2018. Effects of biochar levels on winter wheat yield and water-use efficiency in Loess Plateau. Agricultural Research in the Arid Areas. 36(6), 159–167. https://doi.org/[10.7606/j.issn.l000-7601.2018.06.24](https://doi.org/10.7606/j.issn.l000-7601.2018.06.24). (in Chinese with English abstract).
42 Li, P., Chen, G.P., Gou, Z.W., Yin, W., Fan, Z.L., Hu, F.L., Fan, H., Chai, Q., 2023. Response on light energy utilization and water production benefit of spring wheat to straw retention in an oasis irrigated area. Acta Agron. Sin. 49(5), 1316–1326. https://doi.org/10.3724/SP.J.1006.2023.2103. (in Chinese with English abstract).
43 Guan, X.K., Wang, J.L., Liu, Y., Yang, M.D., Wang, H.Z., Wang, H.P., Wang, T.C., 2018. Rotational tillage with straw returning increased dry matter accumulation and utilization efficiency of water and nitrogen in winter wheat. J. Soil Water Conserv. 32(3), 280–288. https://doi.org/CNKI:SUN:TRQS.0.2018-03-042. (in Chinese with English abstract).
44 Cong, P., Li, Y.Y., Wang, J., Pang, H.C., Zhang, L., Liu, N., Gao, J.S., 2020. Effect of one-off bury of different amounts of straws at 40 cm deep on subsoil fertility. J. Plant Nutr. Fert. 26(1), 74–85. https://doi.org/10.11674/zwyf.19048. (in Chinese with English abstract).
45 Hu, F.L., Chai, Q., Gan, Y.T., Yin, W., Zhao, C., Feng, F.X., 2016. Characteristics of soil carbon emission and water utilization in wheat/maize intercropping with minimal/zero tillage and straw retention. Acta Agron. Sin. 49(1), 120–131. https://doi.org/[10.3864/j.issn.0578-1752.2016.01.011](https://doi.org/10.3864/j.issn.0578-1752.2016.01.011). (in Chinese with English abstract).
46 Liu, W.L., Li, X.P., Shen, X., Zhu, B., 2014. Effects of fertilizer application regimes on soil N2O emissions in the croplands of purple soil in the Sichuan Basin during wheat season. Chinese Chin. J. Eco-Agric. 22(9), 1029–1037. https://doi.org/10.13930/j.cnki.cjea.140067. (in Chinese with English abstract).
47 Kong, D.J., Zhu, J.X., Ren, C.J., Ren, G.X., Feng, Y.Z., Yang, G.H., Liu, N.N., 2022. Effects of straw return on soil carbon and nitrogen components and crop yield under long-term wheat-soybean rotation. Agricultural Research in the Arid Areas. 40(5), 190–200. https://doi.org/[10.7606/j.issn.1000-7601.2022.05.21](https://doi.org/10.7606/j.issn.1000-7601.2022.05.21). (in Chinese with English abstract).
48 Dai, Y.S., Cheng, X., Liu, B.Y., He, X., Hu, M.Y., Yang, W.K., Wang, L.C., Wu, H.Y., Li, Q., Wu, J.H., 2021. Impacts of synergistic mulching of straw and milk vetch on soil nutrients, enzyme activities and wheat yield in upland of southwest China. Chin. J. Soil Sci. 52(6), 1339–1347. https://doi.org/10.19336/j.cnki.trtb.2021041101. (in Chinese with English abstract).
49 Su, Y.Y., Wang, Y.Q., Mei, B.L., Yao, Z.S., 2021. Effects of direct straw return and burning ash amendment on soil NO fluxes in a winter wheat cropland. J. Qinghua Univ. 61(12), 1462–1471. https://doi.org/[10.16511/j.cnki.qhdxxb.2020.25.039](https://doi.org/10.16511/j.cnki.qhdxxb.2020.25.039). (in Chinese with English abstract).
50 Chen, J., Zhang, J.G., Zhao, Y., Zhang, A.F., Cheng, G., Feng, H., 2018. Effects of straw and biochar amendment on greenhouse gases emission in wheat-maize rotation cropland in Guanzhong area. Res. Soil Water Conserv. 25(5), 170–178. https://doi.org/10.13869/j.cnki.rswc.2018.05.024. (in Chinese with English abstract).
51 Wu, Y.H., Hao, X.S., Tian, X.H., Chen, H., Zhang, C.H., Cui, Y.Z., Qin, Y.H., 2020. Effect of straw returning combined with NPK fertilization on soil carbon sequestration and economic benefits under rice-wheat rotation in Hanzhong basin. Acta Agron. Sin. 46(2), 259–268. https://doi.org/10.3724/SP.J.1006.2020.92013. (in Chinese with English abstract).
52 Wang, Z.Y., Bai, Y.L., Gao, J.H., Wang, L., Lu, Y.L., Yang, L.P., 2013. Effect of straw returned to soil and application of potassium fertilizer on crops yield and soil potassium in the low fertility of fluvo-aquic soil in North China. Soil Fert. Sci. China. 2, 46–50. https://doi.org/10.11838/sfsc.20130210. (in Chinese with English abstract).
53 Zhang, S.Q., Tu, H.Z., Yue, K., Huang, S.M., Zhang, B., Song, X., Guo, D.D., Zhang, K.K., Yue, Y.J., 2024. Effects of different proportions of loss-control urea on properties of fluvo-aquic soil and wheat yield in north China. J. Agric. Resour. Environ. 41(1), 105–112. https://doi.org/10.13254/j.jare.2022.0798. (in Chinese with English abstract).
54 Hou, Y.Q., Huang, M., Fu, G.Z., Li, Y.J., Tian, W.Z., Li, J.H., Wu, J.Z., Zhao, K.N., Zhang, Z.W., Zhao, Z.M., Lü, J.J., Yao, Y.Q., 2022. Effects of straw return replacing the application of potassium fertilizer on crop productivity and soil nitrate accumulation in dryland summer maize-winter wheat rotation system. J. Soil Water Conserv. 36(5), 311–318. https://doi.org/10.13870/j.cnki.stbcxb.2022.05.038. (in Chinese with English abstract).
55 Zhao, H.L., Dong, J.J., Shi, J.L., Xu, M., Tian, X.H., 2021. Effect of straw returning mode on soil organic carbon sequestration. Acta Pedologica Sinica. 58(1), 213–224. https://doi.org/[10.11766/trxb201909020267](https://doi.org/10.11766/trxb201909020267). (in Chinese with English abstract).
56 Zhou, D.D., Zhang, J., Li, F.J., Li, B.Z., Zhang, Y.J., Liu, Z.H., Ge, M.J., Zhu, X.H., Li, C.Y., 2021. Effect of straw returning and seed dressing with chemicals on grain yield and quality of wheat following rice. J. Triticeae Crops. 41(11), 1392–1402. <https://doi.org/10.7606/j.issn.1009-1041.2021.11.09>. (in Chinese with English abstract).
57 Ma, C., Zhou, J., Zhu, Y.P., Chai, R.S., Wang, M.Z., Gao, H.J., 2018. Effects of returning maize straw into field on the growth and nitrogen use efficiency of winter wheat under promoting decay condition. J. Anhui Agric. Univ. 45(3), 532–537. https://doi.org/10.13610/j.cnki.1672-352x.20180620.018. (in Chinese with English abstract).
58 Liu, H.J., Ren, D.C., Ni, Y.J., Ge, J., Zhang, S.Y., Lü, G.H., Hu, X., 2023. Effects of straw returning and reducing nitrogen application on soil nutrients, enzyme activities and wheat yield. Crops. 4, 210–214. https://doi.org/10.16035/j.issn.1001-7283.2023.04.030. (in Chinese with English abstract).
59 Wu, Y.H., Hao, X.S., Tian, X.H., Chen, Y.L., Zhang, C.H., Cheng, H., Li, H.H., Qin, Y.H., Zhao, S.L., 2018. Effects of reduction of NPK fertilizer application rates plus rice straw return on soil nutrient, enzyme activities and wheat yield in rice-wheat rotation system. Southwest China Journal of Agricultural Sciences. 31(5), 998–1005. https://doi.org/[10.16213/j.cnki.scjas.2018.5.020](https://doi.org/10.16213/j.cnki.scjas.2018.5.020). (in Chinese with English abstract).
60 Yang, J., Wang, C.Q., Bai, G.C., You, L.Y., Yi, Y.L., Huang, F., Li, X.X., 2015. Life cycle energy consumption and greenhouse gas emissions of wheat-rice rotation system with straw returning. J. Agro-Environ. Sci. 34(1), 196–204. https://doi.org/10.11654/jaes.2015.01.028. (in Chinese with English abstract).
61 Wang, H.Z., Zhang, D.X., Zhang, J., Ma, S.K., Chai, X.R., Zhang, J.H., 2023. Effects of straw returning and phosphorus fertilizer application on grain filling characteristics and phosphorus absorption and utilization of dryland wheat. Soil Fert. Sci. China. 10, 50–159. https://doi.org/10.11838/sfsc.1673-6257.22460. (in Chinese with English abstract).
62 Zhang, J., 2023. Impacts of straw returning combined with nitrogen fertilizer on physiological and biochemical traits and yield of dryland wheat. Jiangsu Agric Sci. 51(23), 61–69. https://doi.org/10.15889/j.issn.1002-1302.2023.23.010. (in Chinese with English abstract).
63 You, L.Y., Li, B., Wang, C.Q., Yang, J., Bai, G.C., Huang, C., 2015. Effects of different amount of straw incorporation on grain yield, nitrogen uptake and use efficiency in wheat-rice rotation system. J. Nucl. Agric. Sci. 30(12), 2394–2401. https://doi.org/[10.11869/j.issn.100-8551.2015.12.2394](https://doi.org/10.11869/j.issn.100-8551.2015.12.2394). (in Chinese with English abstract).
64 Li, T., He, C.E., Ge, X.Y., Zhang, O.Y., 2016. Responses of soil mineral N contents, enzyme activities and crop yield to different C/N ratio mediated by straw retention and N fertilization. Chin. J. Eco-Agric. 24(12), 1633–1642. https://doi.org/10.13930/j.cnki.cjea.160357. (in Chinese with English abstract).
65 Chen, J., Tang, Y.H., Yin, Y.P., Pang, D.W., Cui, Z.Y., Zheng, M.J., Peng, D.L., Yang, W.B., Yang, D.Q., Li, Y.X., Wang, Z.L., Li, Y., 2015. Effects of straw returning plus nitrogen fertilizer on nitrogen utilization and grain yield in winter wheat. Acta Agron. Sin. 41(1), 160–167. https://doi.org/10.3724/sp.J.1006.2015.00160. (in Chinese with English abstract).
66 Hou, H.Z., Zhang, X.C., Yin, J.D., Fang, Y.J., Wang, H.L., Yu, X.F., Ma, Y.F., Zhang, G.P., Lei, K.N., 2020. Effects of straw returning amount on water use and yield of spring wheat under soil-plastic mulching and hole sowing in dryland. J. Triticeae Crops. 41(4), 457–464. https://doi.org/10.7606/j.issn.1009–1041.2021.04.09. (in Chinese with English abstract).
67 Song. J. J., Xu, X.Y., Bai, J.Z., Yu, Q., Cheng, B.H., Feng, Y.Z., Ren, G.X., 2022. Effects of straw returning and fertilizer application on soil nutrients and winter wheat yield. J. Environ. Sci. 43(9), 4839–4847. https://doi.org/10.13227/j.hjkx.202112043. (in Chinese with English abstract).
68 Chen, Y.Z., Chai, S.X., Cheng, H.B., Chai, Y.W., Yang, C.G., Tan, K.M., Chang, L., 2019. Effects of straw-incorporation combined with autumn plastic mulching on soil water consumption characteristics and winter wheat yield in arid farming areas. Acta Agron. Sin. 45(2), 256–266. https://doi.org/10.3724/sp.J.1006.2019.71081. (in Chinese with English abstract).
69 Zhen, L.S., Gu, J., Gao, H., Qin, Q.J., Chen, Q.L., 2012. Effect of straws, manure and chemical fertilizer on soil properties and crop yields. Acta Bot. Boreali-Occident. Sin. 32(9), 1811–1818. https://doi.org/10.3969/j.issn.1000-4025.2012.09.015. (in Chinese with English abstract).
70 Zhao, J.P., Quan, B.Q., Guo, P.Y., Ren, J.C., 2021. Effects of straw returning and nitrogen application on soil quality and wheat yield. Acta Agric Boreali-Sinica. 36(2), 176–181. https://doi.org/10.7668/hbnxb.20191555. (in Chinese with English abstract).
71 Hu, N.J., Han, X.Z., Yang, M.F., Zhang, Z.W., Bian, X.M., Zhu, L.Q., 2023. Short-term influence of straw return on the contents of soil organic carbon fractions, enzyme activities and crop yields in rice-wheat rotation farmland. J. Plant Nutr. Fert. 29(3), 1–7. https://doi.org/10.11674/zwyf.2015.0211. (in Chinese with English abstract).
72 Chang, L., Han, F.X., Chai, Y.W., Yang, D.L., Yang, C.G., Cheng, H.B., Chai, S.X., 2018. Effect of straw belt-mulching on soil temperature and yield of winter wheat in rain-fed semiarid region. Chin. J. Appl. Ecol. 29(9), 2949–2958. https://doi.org/10.13287/j.1001-9332.201809.023. (in Chinese with English abstract).
73 Liu, T., Jia, Z.K., Zhang, R., Zheng, J.C., Ren, S.C., Yang, B.P., Wang, H.X., 2010. Effect of straw mulching on soil moisture and water use efficiency of winter wheat in dryland. Acta Agric. Boreali-Occident. Sin. 38(7), 68–76. https://doi.org/10.3724/SP.J.1006.2025.41083. (in Chinese with English abstract).
74 Zhang, Y.F., 2007. Effects on the soil-moisture and seed yield of straw mulch. Gansu Agricultural Science and Technology. 6, 14–15. https://doi.org/10.3724/SP.J.1006.2014.01787. (in Chinese with English abstract).
75 Gao, C.C., Fu, Y.L., Wang, S.S., 2011. Effects of straw mulch on yield and water use efficiency in winter wheat. China Rural Water and Hydropower. 7, 12–15. https://doi.org/10.3969/j.issn.1001-1463.2007.06.005. (in Chinese with English abstract).
76 Tan, K.M., Yang, C.G., Chai, S.X., Chang, L., Cheng, H.B., Huang, C.X., Wang, Y.P., 2015. Effect of mulching film after straw returning on soil temperature and grain yield of dryland winter wheat. Agricultural Research in the Arid Areas. 33(1), 159–164. https://doi.org/ 10.16302/j.cnki.1000-7601.2015.01.026. (in Chinese with English abstract).
77 Huang, T.M., Zheng, X.F., Hou, Y.Y., Li, X., Wang, Z.H., 2015. Yield and N, P and K uptake and utilization of winter wheat affected by straw return to soil. J. Plant Nutr. Fert. 21(4), 853–863. https://doi.org/10.11674/zwyf.2015.0404. (in Chinese with English abstract).
78 Xiang, X.L., Chen, S.H., Yang, H.K., Yang, Y.H., Fan, G.Q., 2021. Effects of straw mulching and phosphorus application on wheat yield, phosphorus absorption and utilization in hilly dryland. Sci. Agric. Sin. 54(24), 5194–5205. https://doi.org/10.3864/j.issn.0578-1752.2021.24.003. (in Chinese with English abstract).
79 Tu, C., Wang, J., Guan, Q., Liu, W.Z., 2013. Effect of straw mulching on soil respiration, crop yield, economy-environment benefit in rainfed winter wheat fields. Chin. J. Eco-Agric. 21(7), 931–937. https://doi.org/10.3724/sp.J.1011.2013.00931. (in Chinese with English abstract).
80 Wang, P.B., Zhang, D.X., Qiao, C.C., Huang, M., Wang, H.Z., 2025. Effects of straw returning and phosphorus application on soil enzyme activity and yield formation of wheat in dry land of western Henan, China. Acta Agron. Sin. 51(2), 534–547. https://doi.org/10.3724/SP.J.1006.2025.41030. (in Chinese with English abstract).
81 Han, M.M., Li, W.Q., 2024. Effects of straw returning and basal/topdressing ratio of nitrogen fertilizer on grain yield and nitrogen efficiency of winter wheat. Chin. Agric. Sci. Bull. 40(29), 39–44. https://doi.org/10.3724/SP.J.1006.2015.00160. (in Chinese with English abstract).
82 Chen, S.H., Xiang, X.L., Lei, F., Zou, Q.S., Ai, D.L., Zheng, T., Huang, X.L., Fan, G.Q., 2022. Relationship between rhizosphere fungal community and wheat yield under straw mulching combined with nitrogen fertilizer. Acta Ecol. Sin. 42(21), 8751–8761. https://doi.org/10.5846/stxb202112143553. (in Chinese with English abstract).
83 Cheng, B.H., Bai, J.Z., Liu, Z.Y., Song, J.J., Xu, H.W., Wang, X., Ren, G.X., Feng, Y.Z., 2023. Short-term effects of straw return on N2O emissions from wheat and maize rotation fields in Guanzhong area. J. Plant Nutr. Fert. 29(6), 1002–1012. https://doi.org/10.11674/zwyf.2022562. (in Chinese with English abstract).
84 Zhao, H.B., He, J., Li, H.W., Wang, Q.J., Li, W.Y., Liu, W.Z., 2018. Effect of straw returning manners on seedbed soil physical properties and winter wheat growth. Trans. Chin. Soc. Agric. Mach. 49(S1), 60–67. https://doi.org/CNKI:SUN:NYJX.0.2018-S1-009. (in Chinese with English abstract).
85 Liu, P.P., Zhou, Y., Fu, G.X., Gao, X., Zhang, P., Zhang, L., Wang, J.F., 2023. Effects of fertilization on crop yield and soil phosphorus availability based on the returning straw. J. Nanjing Agric. Univ. 46(5), 1–7. https://doi.org/[10.7685/j.issn.1000-2030.2014.05.005](https://doi.org/10.7685/j.issn.1000-2030.2014.05.005). (in Chinese with English abstract).
86 Guan, Q., Wang, J., Song, S.Y., Liu, W.Z., 2011. Influences on different treatments on soil moisture and water use efficiency winter wheat field in arid-highland of the Loess Plateau. Ground Water. 33(1), 21–24. https://doi.org/10.3969/j.issn.1004-1184.2011.01.008. (in Chinese with English abstract).
87 Wang, J., Sun, S.Q., Li, X., Gao, F.J., Wu, Q.Q., Shi, G.M., Hu, K., Geng, L.Z., Ma, X.W., 2023. Effects of different straw returning methods on yield, soil nutrients and sucrase activity of wheat-maize rotation. Jiangsu Agric Sci. 51(4), 85–90. https://doi.org/10.15889/j.issn.1002-1302.2023.04.013. (in Chinese with English abstract).
88 Zhao, J.H., Zhang, C.Z., Zhang, J.B., 2016. Effect of straw returning via deep burial coupled with application of fertilizer as primer on soil nutrients and winter wheat yield. Acta Pedologica Sinica. 53(2), 438–449. https://doi.org/10.11766/trxb201506010162. (in Chinese with English abstract).
89 Ma, C., Zhou, J., Zheng, X.B., Liu, M.Q., Li, H.X., Jiang, Z.S., Wang, W.G., 2012. Effects of returning rice straw into field on soil nutrients and wheat yields under promoting decay condition. Soils. 44(1), 30–35. https://doi.org/10.13758/j.cnki.tr.2012.01.007. (in Chinese with English abstract).
90 Yang, B.W., Liang, X.R., Qin, M.G., Cao, Y.J., Xiong, H., Zhan, M., 2023. Sustainability analysis of different upland-paddy rotation systems in the middle reaches of the Yangtze River based on energy efficiency and carbon efficiency. Acta Agron. Sin. 49(8), 1–17. https://doi.org/10.3724/SP.J.1006.2024.32052. (in Chinese with English abstract).
91 Zhao, Y.L., Xue, Z.W., Guo, H.B., Mu, X.Y., Li, C.H., 2014. Effects of tillage and straw returning on water consumption characteristics and water use efficiency in the winter wheat and summer maize rotation system. Sci. Agric. Sin. 47(17), 3359–3371. https://doi.org/10.3864/j.issn.0578-1752.2014.17.004. (in Chinese with English abstract).
92 Zheng, Y.Z., Nie, C., Tian, X.F., Zhai, S., Sun, S.C., 2022. Response of winter wheat yield and water use efficiency to biochar and straw returning. Water Saving Irrigation. 3, 20–26. https://doi.org/1007-4929(2022)03-0020-07. (in Chinese with English abstract).
93 Ji, S.Y., Wang, X.D., Shi, S.B., Chen, W.B., Ye, Z.Q., Ren, Z.T., Liu, Z., Yu, Z.W., 2020. Effects of rice-straw returning to the field and fertilizer application on soil fertility and wheat yield in rice stubble wheat field. Jiangsu Agric Sci. 36(5), 1181–1188. https://doi.org/[10.3969/j.issn.1000-4440.2020.05.015](https://doi.org/10.3969/j.issn.1000-4440.2020.05.015). (in Chinese with English abstract).
94 Meng, Q.Y., Wang, Y.H., Jin, H.Y., Chao, Y.E., Duan, J.Z., Guo, T.C., 2016. Effect of tillage and straw returning on soil enzyme activity and yield of winter wheat in lime concretion black soil. J. Triticeae Crops. 36(3), 341–346. https://doi.org/10.7606/j.issn.1009-1041.2016.03.12. (in Chinese with English abstract).
95 Gu, K.J., Zhang, S.M., Gu, D.X., Zhang, C.H., Shi, Z.L., Xu, B., Yang, S.J., 2015. Effects of rice straw returning and compaction on grain yield and quality of wheat. Acta Agric. Nucl. Sin. 29(11), 2192–2197. https://doi.org/10.11869/j.issn.100-8551.2015.11.2192. (in Chinese with English abstract).
96 Fan, Y.D., Chai, S.X., Cheng, H.B., Chen, Y.Z., Yang, C.G., Huang, C.X., Chang, L., Pang, L., 2013. Effects of mulching on soil moisture in a dryland winter wheat field, Northwest China. Chinese Journal of Applied Ecology. 24(11), 3137–3144. https://doi.org/CNKI:SUN:YYSB.0.2013-11-017. (in Chinese with English abstract).
97 Zhang, X.Q., Zhao, X.L., Wang, Y.Q., Pu, C., Baoer, J., Chen, F., Zhang, H.L., 2017. Effects of tillage practices on photosynthetic performance diurnal variation during filling stage and grain yield of winter wheat. Chin. J. Appl. Ecol. 28(3), 885–893. https://doi.org/10.13287/j.1001-9332.201703.020. (in Chinese with English abstract).
98 Chen, H.L., Tian, X.H., Wang, X.F., Cao, Y.X., Wu, Y.H., Wang, Z.H., 2010. Effects of different cultivation models on soil water, soil temperature and yield during the winter wheat growth in the Weibei Dry Highland. Chin. J. Appl. Ecol. 30(9), 2424–2433. https://doi.org/CNKI:SUN:STXB.0.2010-09-023. (in Chinese with English abstract).
99 Yin, W., Chen, G.P., Guo, Y., Fan, Z.L., Hu, F.L., Fan, H., Yu, A.Z., Zhao, C., Chai, Q., 2020. Responses of dry matter accumulation and yield in a following maize crop to spring wheat straw returning. Chin. J. Eco-Agric. 28(8), 1210–1218. https://doi.org/10.13930/j.cnki.cjea.200164. (in Chinese with English abstract).
100 Yang, X.M., Yin, C.B., Li, G.C., Chien, H.P., Nagumo, F., 2016. Effects of reducing nitrogen application and replacing part of nitrogen fertilizer by crop residue on nitrogen leaching in winter wheat-summer corn system. Chin. J. Agric. Resour. Reg. Plan. 37(7), 116–122. https://doi.org/10.7621/cjarrp.1005-9121.20160717. (in Chinese with English abstract).
101 Tang, Y.L., Li, C.S., Wu, C., Ma, X.L., Huang, G., 2012. Long-lasting effect of year-round tillage patterns on yield and grain quality of wheat in Chengdu Plain of China. Sci. Agric. Sin. 45(18), 3721–3732. https://doi.org/10.3864/j.issn.0578-1752.2012.18.005. (in Chinese with English abstract).
102 Zhang, D.X., Ma, S.K., Zhang, J., Shen, S.H., Wang, H.Z., 2022. Influences of different tillage methods on soil nutrient evolution, grain nutrient and yield of dryland wheat after anthesis. Jiangsu Agric Sci. 50(24), 73–78. https://doi.org/[10.15889/j.issn.1002-1302.2022.24.010](https://doi.org/10.15889/j.issn.1002-1302.2022.24.010). (in Chinese with English abstract).
103 Zhou, Z.P., Tian, B.G., Chen, W.H., Wang, Z.Y., Yuan, W., Liu, S.P., 2021. Effects of different tillage methods and straw returning on soil nutrients and wheat yield and quality. Crops. 3, 78–83. <https://doi.org/>10.16035/j.issn.1001-7283.2021.03.012. (in Chinese with English abstract).
104 Hou, L.T., Jiang, X.D., Han, B., Jiao, N.Y., Zhao, C., Li, Z.J., 2006. Effects of different mulching treatments on the gas exchange parameters and water use efficiency of winter wheat. Trans. Chin. Soc. Agric. Mach. 9, 58–63. (in Chinese with English abstract).
105 Yang, J., Shen, Y.Y., Nan, Z.B., Gao, C.Y., Niu, Y.N., Wang, X.Z., Li, G.D., 2010. Effects of conservation tillage on crop yield and carbon pool management index on top soil within a maize-wheat-soy rotation system in the Loess Plateau. Acta Prataculturae Sinica. 19(1), 75–82. <https://doi.org/>10.11686/cyxb20100111. (in Chinese with English abstract).
106 Ni, Q.Q., Cai, H.M., Wu, Y., Jiang, G., Xu, B., Wei, F.Z., Chen, X., Li, J.C., 2023. Effects of straw returning in different seasons on dry matter accumulation, distribution and nitrogen use efficiency of winter wheat. J. Triticeae Crops. 43(1), 75–80. https://doi.org/10.7606/j.issn.1009-1041.2023.01.08. (in Chinese with English abstract).
107 Ding, S.J., Guo, D.D., Zhang, S.Q., Song, X., Zhang, K.K., Guo, T.F., Yue, K., Huang, S.M., Zhou, G.Q., 2024. Effects of 31-year long-term fertilization on the evolution of trace elements in fluvo-aquic soil and their uptake by wheat. J. Plant Nutr. Fert. 30(10), 1858–1871. https://doi.org/10.11674/zwyf.2024141. (in Chinese with English abstract).
108 Li, C.X., Chen, H.T., Ma, S.C., Zhang, L.L., Wang, J.R., Shao, Y., 2016. Effects of different tillage methods on carbon storage of soil and utilization efficiency of water and nitrogen in wheat. Acta Agric Boreali-Sinica. 31(4), 220–226. https://doi.org/10.7668/hbnxb.2016.04.034. (in Chinese with English abstract).
109 Yan, C.P., Zhang, Y.M., Hu, C.S., Dong, W.X., Wang, Y.Y., Li, X.X., Qin, S.P., 2016. Greenhouse gas exchange and comprehensive global warming potential under different wheat-maize rotation patterns. Chin. J. Eco-Agric. 24(6), 704–715. https://doi.org/10.13930/j.cnki.cjea.160261. (in Chinese with English abstract).
110 Zheng, X.B., 2012. Effects of different fertilization treatments on yields and nitrogen utilization of winter wheat-summer maize rotation system in region along Huai River. Soils. 44(3), 402–407. https://doi.org/10.13758/j.cnki.tr.2012.03.023. (in Chinese with English abstract).
111 Wu, C.F., Wen, Q., Han, Y.L., Wang, Q., Jiang, Y., Li, P.P., 2017. Effect of water retaining agent and straw applications on water and fertilizer status and wheat yield in sandy Chao soil. Chin. J. Soil Sci. 48(3), 692–700. https://doi.org/10.19336/j.cnki.trtb.2017.03.26. (in Chinese with English abstract).
112 Xu, Y., Liu, Z.H., Zhu, G.L., Li, X., Tan, D.S., Shi, J., Jiang, L.H., 2016. Effects of greenhouse gas emission under different agricultural management practices in wheat field in the North China Plain. Soil Fert. Sci. China. 2, 7–13. https://doi.org/10.11838/sfsc.20160202. (in Chinese with English abstract).
113 Chen, X., Tang, Z.W., Weng, Y., Cai, H.M., Huang, W.X., Wang, F.F., Wang, R., Zheng, B.Q., Li, J.C., 2024. Effects of straw returning modes on the superior/inferior grain filling of winter wheat. Trans. Chin. Soc. Agric. Mach. 40(12), 128–137. https://doi.org/10.11975/j.issn.1002-6819.202401012. (in Chinese with English abstract).
114 Zhang, D.X., Han, Z.Q., Liu, W., Gao, S.G., Hou, D.J., Li, G.F., Chang, L.S., 2005. Biological effect of maize stalk return to field directly under different accretion decay conditions. Plant Nutr. Fert. Sci. 6, 36–43. https://doi.org/10.11674/zwyf.2005.0606. (in Chinese with English abstract).
115 Ji, Q., Sun, H.Y., Ak, T., Wang, X.D., 2014. Impact of different tillage practices on soil organic carbon and water use efficiency under continuous wheat-maize binary cropping system. Chin. J. Ecol. 25(4), 1029–1035. https://doi.org/10.13287/j.1001-9332.2014.0074. (in Chinese with English abstract).
116 Yang, Y.H., Wu, J.C., Zhang, J.M., Pan, X.Y., Wang, Y., He, F., Han, W.F., 2017. Effects of different tillage measures maintaining soil moisture on water consumption characteristics and anniversary water utilization for wheat and maize. Acta Agric Boreali-Sinica. 32(3), 103–110. https://doi.org/10.7668/hbnxb.2017.03.016. (in Chinese with English abstract).
117 Kang, Y.X., Song, M.D., Han, M., Li, Z.P., 2024. Effects of different organic materials returning to field on plant growth and water use under model of multiple cropping of green fertilizer after wheat. Jiangsu Agric Sci. 52(9), 248–254. https://doi.org/10.15889/j.issn.1002－1302.2024.09.034. (in Chinese with English abstract).
118 Zheng, B.Z., 2014. Effect of different soil surface mulching patterns on yield of winter wheat and their mechanism. Guizhou Agric. Sci. 42(11), 114–118. https://doi.org/10.3969/j.issn.1001-3601.2014.11.029. (in Chinese with English abstract).
119 Zhang, G., Zhang, S.J., Wang, D.J., Yu, Y.C., 2020. Analysis of yield and economic benefit of straw incorporation under rice-wheat double cropping system. Crops. 6, 97–103. https://doi.org/10.16035/j.issn.1001-7283.2020.06.013. (in Chinese with English abstract).
120 Kong, F.L., Yuan, J.C., Zhang, H.L., Chen, F., 2013. Effect of tillage practice on growth and development and yield of winter wheat in double cropping area in North China. Acta Agron Sin. 39(9), 1612–1618. https://doi.org/10.3724/sp.J.1006.2013.01612. (in Chinese with English abstract).
121 Wang, Z.Y., Bai, Y.L., Yang, L.P., Lu, Y.L., Wang, L., Wang, H., 2012. Effects of application of potassium fertilizer and straw returning on crop yields and soil potassium balance in low-yielding fields. J. Plant Nutr. Fert. 18(4), 900–906. https://doi.org/CNKI:SUN:ZWYF.0.2012-04-017. (in Chinese with English abstract).
122 Sheng, H.J., Niu, D., Zhang, C.X., Sun, K.W., Zhu, X.K., 2018. Impacts of microbial inoculants for straw decomposing on greenhouse gas emission in wheat season of the wheat-rice rotation system. J. Yangzhou Univ. Agric. Life Sci. Ed. 39(2), 29–34. https://doi.org/[10.16872/j.cnki.1671-4652.2018.02.005](https://doi.org/10.16872/j.cnki.1671-4652.2018.02.005). (in Chinese with English abstract).
123 Hu, Z.Z., Yi, Z.W., Yang, D.L., Wang, A., Chen, L.G., Zhang, Y.F., Wang, X., 2022. Effects of nitrogen reduction and peanut straw returning on ammonia volatilization, nitrogen use efficiency and grain yield in wheat field. Jiangsu Agric Sci. 38(6), 1492–1499. https://doi.org/10.3969/j.issn.1000-4440.2022.06.006. (in Chinese with English abstract).
124 Wang, N., Liu, Y.G., Zhang, H.S., Li, L.Y., Lin, Q., 2012. Effects of coupling of precise straw-return and nitrogen fertilizer on photosynthetic characteristics after anthesis and yield of winter wheat. Acta Agric Boreali-Sinica. 27(6), 185–190. https://doi.org/[10.3969/j.issn.1000-7091.2012.06.036](https://doi.org/10.3969/j.issn.1000-7091.2012.06.036). (in Chinese with English abstract).
125 Zhang, L., Wang, J., Pang, H.C., Zhang, J.T., Guo, J.J., Dong, G.H., 2018. Effects of short-term granulated straw incorporation on grain yield and soil respiration in a winter wheat-summer maize cropping system. Chin. J. Appl. Ecol. 29(2), 565–572. https://doi.org/10.13287/j.1001-9332.201802.030. (in Chinese with English abstract).
126 Zhou, D.D., Zhang, J., Li, F.J., Ge, M.J., Li, B.Z., Liu, Z.H., Zhang, Y.J., Li, C.Y., Zhu, X.K., 2022. Effects of rice straw-returning and tillage modes on yield formation and grain quality of winter wheat. J. Triticeae Crops. 42(10), 1273–1282. https://doi.org/[10.7606/j.issn.1009-1041.2022.10.12](https://doi.org/10.7606/j.issn.1009-1041.2022.10.12). (in Chinese with English abstract).
127 Kong, F.L., Zhang, H.L., Zhai, Y.L., Yuan, J.C., Chen, F., 2014. Effects of tillage methods on crop yield and water use characteristics in winter-wheat/summer-maize rotation system in the North China Plain. Chin. J. Eco-Agric. 22(7), 749–756. https://doi.org/10.3724/SP.J.1011.2014.31232. (in Chinese with English abstract).
128 Chang, L., Han, F.X., Chai, Y.W., Bao, Z.Y., Cheng, H.B., Huang, C.X., Yang, D.L., Chai, S.X., 2019. Effects of bundled straw mulching on water consumption characteristics and grain yield of winter wheat in rain-fed semiarid region. Chin. J. Appl. Ecol. 30(12), 4150–4158. <https://doi.org/>[10.13287/j.1001-9332.201912.026](https://doi.org/10.13287/j.1001-9332.201912.026). (in Chinese with English abstract).
129 Zheng, W., Zhang, J., Liu, Y., Wen, X.X., Liao, Y.C., Gao, M.S., 2009. Physiological effects of ploughing corn straw under soil on flag-leaf resistance of winter wheat under lowly applying fertilizer condition. Acta Ecol. Sin. 29(9), 4967–4975. https://doi.org/[10.3321/j.issn:1000-0933.2009.09.045](https://doi.org/10.3321/j.issn:1000-0933.2009.09.045). (in Chinese with English abstract).
130 Gu, K.J., Zhang, C.H., Gu, D.X., Zhang, S.M., Shi, Z.L., Yang, S.J., 2017. Effects of different straw returning and tillage methods on annual yield and soil nutrients under rice-wheat rotation system in short-term. Southwest China Journal of Agricultural Sciences. 30(6), 1408–1413. https://doi.org/[10.16213/j.cnki.scjas.2017.6.029](https://doi.org/10.16213/j.cnki.scjas.2017.6.029). (in Chinese with English abstract).
131 Ahmad, S., Raza, M.A.S., Ali, M., Iqbal, R., Aslam, M.U., Toleikiene, M., Mustafa, A.E.-Z.M.A., Rizwana, H., Haider, I., Zain, M., Aghayeva, S., Simsek, O., Ahmed, T., 2024. Managing water deficit in wheat through mulches and irrigation techniques in semi-arid regions. Cereal Res. Commun. https://doi.org/10.1007/s42976-024-00607-2.
132 Akhtar, K., Ain, N.U., Wang, W., Ren, G., Feng, Y., Djalovic, I., Prasad, P.V.V., Yang, G., He, B., Wen, R., 2023. Straw mulch decreased nitrogen fertilizer requirements via regulating soil moisture and temperature to improve physiology, nitrogen, and water use efficiency of wheat. Agron J. 115, 3106–3118. https://doi.org/10.1002/agj2.21437.
133 Akhtar, K., Wang, W., Khan, A., Ren, G., Zaheer, S., Sial, T.A., Feng, Y., Yang, G., 2019. Straw mulching with fertilizer nitrogen: An approach for improving crop yield, soil nutrients and enzyme activities. Soil Use Manage. 35, 526–535. https://doi.org/10.1111/sum.12478.
134 Akhtar, K., Wang, W., Ren, G., Khan, A., Enguang, N., Khan, A., Feng, Y., Yang, G., Wang, H., 2020. Straw mulching with inorganic nitrogen fertilizer reduces soil CO2 and N2O emissions and improves wheat yield. Sci. Total Environ. 741. https://doi.org/10.1016/j.scitotenv.2020.140488.
135 Alsamin, B., El-Hendawy, S., Refay, Y., Tola, E., Mattar, M.A., Marey, S., 2022. Integrating Tillage and Mulching Practices as an Avenue to Promote Soil Water Storage, Growth, Production, and Water Productivity of Wheat under Deficit Irrigation in Arid Countries. Agronomy-Basel. 12. https://doi.org/10.3390/agronomy12092235.
136 Bakht, J., Shafi, M., Jan, M.T., Shah, Z., 2009. Influence of crop residue management, cropping system and N fertilizer on soil N and C dynamics and sustainable wheat (Triticum aestivum L.) production. Soil Tillage Res. 104, 233–240. https://doi.org/10.1016/j.still.2009.02.006.
137 Balwinder-Singh, Humphreys, E., Eberbach, P.L., Katupitiya, A., Yadvinder-Singh, Kukal, S.S., 2011. Growth, yield and water productivity of zero till wheat as affected by rice straw mulch and irrigation schedule. Field Crops Res. 121, 209–225. https://doi.org/10.1016/j.fcr.2010.12.005.
138 Brennan, J., Hackett, R., McCabe, T., Grant, J., Fortune, R.A., Forristal, P.D., 2014. The effect of tillage system and residue management on grain yield and nitrogen use efficiency in winter wheat in a cool Atlantic climate. Eur. J. Agron. 54, 61–69. https://doi.org/10.1016/j.eja.2013.11.009.
139 Chai, Y., Chai, Q., Li, R., Li, Y., Yang, C., Cheng, H., Chang, L., Chai, S., 2022. Straw strip mulching in a semiarid rainfed agroecosystem achieves winter wheat yields similar to those of full plastic mulching by optimizing the soil hydrothermal regime. Crop J. 10, 879–892. https://doi.org/10.1016/j.cj.2021.09.004.
140 Chakraborty, D., Garg, R.N., Tomar, R.K., Singh, R., Sharma, S.K., Singh, R.K., Trivedi, S.M., Mittal, R.B., Sharma, P.K., Kamble, K.H., 2010. Synthetic and organic mulching and nitrogen effect on winter wheat (Triticum aestivum L.) in a semi-arid environment. Agric. Water Manag. 97, 738–748. https://doi.org/10.1016/j.agwat.2010.01.006.
141 Chen, H., Liu, J., Zhang, A., Chen, J., Cheng, G., Sun, B., Pi, X., Dyck, M., Si, B., Zhao, Y., Feng, H., 2017. Effects of straw and plastic film mulching on greenhouse gas, emissions in Loess Plateau, China: A field study of 2 consecutive wheat-maize rotation cycles. Sci. Total Environ. 579, 814–824. https://doi.org/10.1016/j.scitotenv.2016.11.022.
142 Chen, S.Y., Zhang, X.Y., Pei, D., Sun, H.Y., Chen, S.L., 2007. Effects of straw mulching on soil temperature, evaporation and yield of winter wheat: field experiments on the North China Plain. Ann. Appl. Biol. 150, 261–268. https://doi.org/10.1111/j.1744-7348.2007.00144.x.
143 Chen, X., Zhao, H., Liu, J., Mao, A., 2020. Winter wheat nitrogen utilization under different mulching practices on the Loess Plateau. Agron J. 112, 1391–1405. https://doi.org/10.1002/agj2.20105.
144 Cheng, D., Wang, Z., Yang, L., Zhang, L., Zhang, Q., 2021. Combined effects of mulching and crop density on soil evaporation, temperature, and water use efficiency of winter wheat. Exp Agric. 57, 163–174. https://doi.org/10.1017/s0014479721000119.
145 Cui, H., Luo, Y., Chen, J., Jin, M., Li, Y., Wang, Z., 2022. Straw return strategies to improve soil properties and crop productivity in a winter wheat-summer maize cropping system. Eur. J. Agron. 133. https://doi.org/10.1016/j.eja.2021.126436.
146 Cui, S., Cao, G., Zhu, X., 2024. Effects of Straw Return Duration on Soil Carbon Fractions and Wheat Yield in Rice-Wheat Cropping System. Sustain. 16. https://doi.org/10.3390/su16020754.
147 Dawar, K., Khan, A., Sardar, K., Fahad, S., Saud, S., Datta, R., Danish, S., 2021. Effects of the nitrification inhibitor nitrapyrin and mulch on N2O emission and fertilizer use efficiency using 15N tracing techniques. Sci. Total Environ. 757. https://doi.org/10.1016/j.scitotenv.2020.143739.
148 Ding, J., Du, Y., Hu, W., Ding, D., Yang, Y., 2023. Effects of conservation tillage with straw mulching on annual yield and water utilization in a winter wheat-summer maize cropping system on the North China Plain. Agron J. 115, 3162–3172. https://doi.org/10.1002/agj2.21447.
149 Ding, J., Wu, J., Ding, D., Yang, Y., Gao, C., Hu, W., 2021. Effects of tillage and straw mulching on the crop productivity and hydrothermal resource utilization in a winter wheat-summer maize rotation system. Agric. Water Manag. 254. https://doi.org/10.1016/j.agwat.2021.106933.
150 Dong, Q.G., Yang, Y., Yu, K., Feng, H., 2018. Effects of straw mulching and plastic film mulching on improving soil organic carbon and nitrogen fractions, crop yield and water use efficiency in the Loess Plateau, China. Agric. Water Manag. 201, 133–143. https://doi.org/10.1016/j.agwat.2018.01.021.
151 Du, M., Yao, Y., Liu, S., Li, G., Yuan, J., 2024. Reducing Nitrogen Application Rates and Straw Mulching Can Alleviate Greenhouse Gas Emissions from Wheat Field Soil and Improve Soil Quality. Agronomy-Basel. 14. https://doi.org/10.3390/agronomy14092087.
152 El-Hendawy, S., Alsamin, B., Mohammed, N., Al-Suhaibani, N., Refay, Y., Alotaibi, M., Tola, E., Mattar, M.A., 2022. Combining Planting Patterns with Mulching Bolsters the Soil Water Content, Growth, Yield, and Water Use Efficiency of Spring Wheat under Limited Water Supply in Arid Regions. Agronomy-Basel. 12. https://doi.org/10.3390/agronomy12061298.
153 Fang, H., Li, Y., Gu, X., Li, Y., Chen, P., 2021. Can ridge-furrow with film and straw mulching improve wheat-maize system productivity and maintain soil fertility on the Loess Plateau of China? Agric. Water Manag. 246. https://doi.org/10.1016/j.agwat.2020.106686.
154 Gao, Y., Li, Y., Zhang, J., Liu, W., Dang, Z., Cao, W., Qiang, Q., 2009. Effects of mulch, N fertilizer, and plant density on wheat yield, wheat nitrogen uptake, and residual soil nitrate in a dryland area of China. Nutr. Cycling Agroecosyst. 85, 109–121. https://doi.org/10.1007/s10705-009-9252-0.
155 Gao, Y., Shao, Y., Wang, J., Hu, B., Feng, H., Qu, Z., Liu, Z., Zhang, M., Li, C., Liu, Y., 2024. Effects of straw returning combined with blended controlled-release urea fertilizer on crop yields, greenhouse gas emissions, and net ecosystem economic benefits: A nine-year field trial. J. Environ Manage. 356. https://doi.org/10.1016/j.jenvman.2024.120633.
156 Huang, G.B., Chai, Q., Feng, F.X., Yu, A. Z., 2012. Effects of Different Tillage Systems on Soil Properties, Root Growth, Grain Yield, and Water Use Efficiency of Winter Wheat (Triticum aestivum L.) in Arid Northwest China. J. Integr. Agric. 11, 1286–1296. https://doi.org/10.1016/S2095-3119(12)60125-7.
157 Guo, J., Jiang, P., Zhang, J., Dong, S., Tian, W., Li, J., Li, F., Lv, J., Yao, Y., Hou, Y., Wu, S., Shaaban, M., Huang, M., Li, Y., 2024. Straw Return Substituting Potassium Fertilizer Increases Crop Yield, Efficiency, and Quality in Maize-Wheat Rotation System. Agronomy-Basel. 14. https://doi.org/10.3390/agronomy14061266.
158 Guo, L., Zhang, L., Liu, L., Sheng, F., Cao, C., Li, C., 2021. Effects of long-term no tillage and straw return on greenhouse gas emissions and crop yields from a rice-wheat system in central China. Agric. Ecosyst. Environ. 322. https://doi.org/10.1016/j.agee.2021.107650.
159 Guo, Y., Fan, H., Li, P., Wei, J., Qiu, H., 2023. Photosynthetic Physiological Basis of No Tillage with Wheat Straw Returning to Improve Maize Yield with Plastic Film Mulching in Arid Irrigated Areas. Plants-Basel. 12. https://doi.org/10.1016/j.agee.2021.107650.
160 Guo, Z., Liu, H., Wan, S., Hua, K., Jiang, C., Wang, D., He, C., Guo, X., 2017. Enhanced yields and soil quality in a wheat-maize rotation using buried straw mulch. J. Sci. Food Agric. 97, 3333–3341. https://doi.org/10.1002/jsfa.8183.
161 Guo, Z., Liu, Y., Meng, X., Yang, X., Ma, C., Chai, H., Li, H., Ding, R., Nazarov, K., Zhang, X., Han, Q., 2024. The long-term nitrogen fertilizer management strategy based on straw return can improve the productivity of wheat-maize rotation system and reduce carbon emissions by increasing soil carbon and nitrogen sequestration. Field Crops Res. 317. https://doi.org/10.1016/j.fcr.2024.109561.
162 He, G., Wang, Z., Li, F., Dai, J., Ma, X., Li, Q., Xue, C., Cao, H., Wang, S., Liu, H., Luo, L., Huang, M., Malhi, S.S., 2016. Soil nitrate-N residue, loss and accumulation affected by soil surface management and precipitation in a winter wheat-summer fallow system on dryland. Nutr. Cycling Agroecosyst. 106, 31–46. https://doi.org/10.1016/j.agwat.2016.03.005.
163 He, G., Wang, Z., Li, F., Dai, P., Li, Q., Xue, C., Cao, H., Wang, S., Malhi, S.S., 2016. Soil water storage and winter wheat productivity affected by soil surface management and precipitation in dryland of the Loess Plateau, China. Agric. Water Manag. 171, 1–9. https://doi.org/10.1007/s10705-016-9787-9.
164 Hou, P., Gao, L., Jiang, P., Yu, J., Liu, X., Jiang, D., Cao, W., Dai, T., Tian, Z., 2025. Straw return amplifies the stimulated impact of night-warming on N2O emissions from wheat fields in a rice-wheat rotation system. Field Crops Res. 320. https://doi.org/10.1016/j.fcr.2024.109652.
165 Hu, N., Shi, H., Wang, B., Gu, Z., Zhu, L., 2019. Effects of different wheat straw returning modes on soil organic carbon sequestration in a rice-wheat rotation. Can J Soil Sci 99, 25–35. https://doi.org/10.1016/j.agee.2016.02.027.
166 Hu, Y., Ma, P., Zhang, B., Hill, R.L., Wu, S., Dong, Q.G., Chen, G., 2019. Exploring optimal soil mulching for the wheat-maize cropping system in sub-humid drought-prone regions in China. Agric. Water Manag. 219, 59–71. https://doi.org/10.1016/j.agwat.2019.04.004.
167 Hu, Y., Sun, B., Wu, S., Feng, H., Gao, M., Ma, P., Zhang, T., Pi, X., 2021. Soil carbon and nitrogen of wheat-maize rotation system under continuous straw and plastic mulch. Nutr. Cycling Agroecosyst. 119, 181–193. https://doi.org/10.1016/j.agwat.2019.04.004.
168 Huang, C., Wu, Y., Ye, Y., Li, Y., Ma, J., Ma, J., Yan, J., Chang, L., Wang, Z., Wang, Y., Zhang, H., 2022. Straw Strip Mulching Increases Winter Wheat Yield by Optimizing Water Consumption Characteristics in a Semi-Arid Environment. Water. 14. https://doi.org/10.3390/w14121894.
169 Jat, R.K., Sapkota, T.B., Singh, R.G., Jat, M.L., Kumar, M., Gupta, R.K., 2014. Seven years of conservation agriculture in a rice-wheat rotation of Eastern Gangetic Plains of South Asia: Yield trends and economic profitability. Field Crops Res. 164, 199–210. https://doi.org/10.1016/j.fcr.2014.04.015.
170 Jin, C., Meng. J.Z., Dang. W.P., Y. P.Y., Han. M.M., Li. Y.X., Li. Y.L., X, X., Li. Y., Wang. Z.L., 2017. Straw return and appropriate tillage method improve grain yield and nitrogen efficiency of winter wheat. J. Integr Agric. 16, 1708–1719. https://doi.org/10.1016/s2095-3119(16)61589-7.
171 Kong, F., Hu, S., Wang, R., Jiu, A., Kan, Z., Yang, H., Palta, J.A., Li, F.M., 2024. Straw return under deep tillage increases grain yield in the rice-rotated wheat cropping system. Field Crops Res. 317. https://doi.org/10.1016/j.fcr.2024.109559.
172 Legesse, N., Wu, S., Wang, Y., Gan, M.Q., Liu, P.S., Huang, Y., Xu, H.J., Hu, H.X., Ma, Y.H., 2023. Optimal fertilizer rates towards the improvement of nitrogen use efficiency and reduction of nitrogen export in paddy rice-wheat intensive farming. Front. Environ. Sci. 11. https://doi.org/10.3389/fenvs.2023.1239785.
173 Li, J., Guo, Z., Ye, S., 2024. Minor straw return enhances net income in a maize-wheat rotation system. Front. Environ. Sci. 12. https://doi.org/10.3389/fenvs.2024.1343751.
174 Li, J., Li, Y., Lin, N., Fang, Y., Dong, Q.g., Zhang, T., Siddique, K.H.M., Wang, N., Feng, H., 2024. Ammoniated straw returning: A win-win strategy for increasing crop production and soil carbon sequestration. Agric. Ecosyst. Environ. 363. https://doi.org/10.1016/j.agee.2023.108879.
175 Li, P., Yin, W., Fan, Z., Hu, F., Zhao, L., Fan, H., He, W., Chai, Q., 2024. Improving crop productivity by optimizing straw returning patterns to delay senescence of wheat leaves. Eur. J. Agron. 159. https://doi.org/10.1016/j.eja.2024.127274.
176 Li, R., Chai, S., Chai, Y., Li, Y., Lan, X., Ma, J., Cheng, H., Chang, L., 2021. Mulching optimizes water consumption characteristics and improves crop water productivity on the semi-arid Loess Plateau of China. Agric. Water Manag. 254. https://doi.org/10.1016/j.agwat.2021.106965.
177 Li, X., Shi, J., Chen, J., Tian, X., 2024. Beneficial Effects on Winter Wheat Production of the Application of Legume Green Manure during the Fallow Period. Agronomy-Basel. 14. https://doi.org/10.3390/agronomy14010203.
178 Li, Y., Abalos, D., Arthur, E., Feng, H., Siddique, K.H.M., Chen, J., 2024. Different straw return methods have divergent effects on winter wheat yield, yield stability, and soil structural properties. Soil Tillage Res. 238. https://doi.org/10.1016/j.still.2023.105992.
179 Li, Y., Chai, S., Chai, Y., Li, R., Lan, X., Ma, J., Cheng, H., Chang, L., 2021. Effects of mulching on soil temperature and yield of winter wheat in the semiarid rainfed area. Field Crops Res. 271. https://doi.org/10.1016/j.fcr.2021.108244.
180 Li, Y., Chai, Y., Ma, J., Li, R., Cheng, H., Chang, L., Chai, S., 2022. Straw strip mulching in a semiarid rainfed agroecosystem achieves carbon sequestration and emission reduction from winter wheat fields. Agric. Ecosyst. Environ. 334. https://doi.org/10.1016/j.agee.2022.107990.
181 Li, Z., Lai, X., Yang, Q., Yang, X., Cui, S., Shen, Y., 2018. In search of long-term sustainable tillage and straw mulching practices for a maize-winter wheat-soybean rotation system in the Loess Plateau of China. Field Crops Res. 217, 199–210. https://doi.org/10.1016/j.fcr.2017.08.021.
182 Lin, J., Xu, Z., Xue, Y., Sun, R., Yang, R., Cao, X., Li, H., Shao, Q., Lou, Y., Wang, H., Yang, Q., Pan, H., Zhuge, Y., 2023. N2O emissions from soils under short-term straw return in a wheat-corn rotation system are associated with changes in the abundance of functional microbes. Agric. Ecosyst. Environ. 341. https://doi.org/10.1016/j.agee.2022.108217.
183 Ling, J., Zhou, J., Wu, G., Zhao, D.Q., Wang, Z.T., Wen, Y., Zhou, S.L., 2024. Deep-injected straw incorporation enhances subsoil quality and wheat productivity. Plant Soil. 499, 207–220. https://doi.org/10.1007/s11104-022-05660-6.
184 Liu, B., Xia, H., Jiang, C., Jiang, C., Riaz, M., Yang, L., Chen, Y., Fan, X., Zhang, Z., Duan, X., Wu, M., Xia, X., 2024. Straw Addition Enhances Crop Yield, Soil Aggregation, and Soil Microorganisms in a 14-Year Wheat-Rice Rotation System in Central China. Plants-Basel. 13. https://doi.org/10.3390/plants13070985.
185 Liu, G., Zuo, Y., Zhang, Q., Yang, L., Zhao, E., Liang, L., Tong, Y.A., 2018. Ridge-furrow with plastic film and straw mulch increases water availability and wheat production on the Loess Plateau. Sci. Rep. 8. https://doi.org/10.1038/s41598-018-24864-4.
186 Liu, Q., Lu, L., Hou, J., Bai, J., Dong, Q.G., Feng, H., Zou, Y., Siddique, K.H.M., 2024. N Fertilizer in Combination with Straw Improves Soil Physicochemical Properties and Crop Productivity in Sub-Humid, Drought-Prone Areas. Agronomy-Basel. 14. https://doi.org/10.3390/agronomy14081721.
187 Liu, X., Ai, Y., Zhang, F., Lu, S., Zeng, X., Fan, M., 2005. Crop production, nitrogen recovery and water use efficiency in rice-wheat rotation as affected by non-flooded mulching cultivation (NFMC). Nutr. Cycling Agroecosyst. 71, 289–299. https://doi.org/10.1007/s10705-004-6801-4.
188 Liu, X., Ren, Y., Gao, C., Yan, Z., Li, Q., 2017. Compensation effect of winter wheat grain yield reduction under straw mulching in wide-precision planting in the North China Plain. Sci. Rep. 7. https://doi.org/10.1038/s41598-017-00391-6.
189 Liu, Z., Chen, Z., Ma, P., Meng, Y., Zhou, J., 2017. Effects of tillage, mulching and N management on yield, water productivity, N uptake and residual soil nitrate in a long-term wheat-summer maize cropping system. Field Crops Res. 213, 154–164. https://doi.org/10.1016/j.fcr.2017.08.006.
190 Liu, Z., Wang, N., Lu, J., Wang, L., Li, G., Ning, T., 2022. Climate-Smart Tillage Practices with Straw Return to Sustain Crop Productivity. Agronomy-Basel. 12. https://doi.org/10.3390/agronomy12102452.
191 Long, M., Li, M., Yu, C., Ding, Y., Li, W., Zhang, H., Zhang, T., Wen, X., 2023. Effects of Straw Returning Method on Soil Physical and Chemical Properties and Growth of Winter Wheat in Rainfed Area of the Loess Plateau. J. Soil Sci. Plant Nutr. https://doi.org/10.1007/s42729-023-01422-0.
192 Luo, C.L., Zhang, X.F., Duan, H.X., Mburu, D.M., Ren, H.X., Kavagi, L., Dai, R.Z., Xiong, Y.C., 2020. Dual plastic film and straw mulching boosts wheat productivity and soil quality under the El Nino in semiarid Kenya. Sci. Total Environ. 738. https://doi.org/10.1016/j.scitotenv.2020.139808.
193 Luo, X., Guo, Y., Wang, R., Wang, N., Li, C., Chu, X., Feng, H., Chen, H., 2021. Carbon footprint of a winter wheat-summer maize cropping system under straw and plastic film mulching in the Loess Plateau of China. Sci. Total Environ. 794. https://doi.org/10.1016/j.scitotenv.2021.148590.
194 Ma, E., Zhang, G., Ma, J., Xu, H., Cai, Z., Yagi, K., 2010. Effects of rice straw returning methods on N_2O_ emission during wheat-growing season. Nutr. Cycling Agroecosyst. 88, 463–469. https://doi.org/10.1007/s10705-010-9369-1.
195 Ma, Y., Liu, D.L., Schwenke, G., Yang, B., 2019. The global warming potential of straw-return can be reduced by application of straw-decomposing microbial inoculants and biochar in rice-wheat production systems. Environ Pollut. 252, 835–845. https://doi.org/10.1016/j.envpol.2019.06.006.
196 Mei, S., Wang, S., Yang, X., 2019. Straw Returning and Nitrogen Application on Winter Wheat Quality and Yield under Wheat-Maize Multiple Cropping System. Ekoloji. 28, 2183–2192. https://doi.org/WOS:000461678300247.
197 Yin, M. H., Li, Y.N., Xue, Y. B., Zhang, C. M., 2018. Effects of mulches on water use in a winter wheat/summer maize rotation system in Loess Plateau, J. Arid Land. 10, 277–291. https://doi.org/10.1007/s40333-018-0092-0.
198 Peng, Z., Ting, W., W, H. X., W, M., M, X. P., Siwei, M., Rui, Z., Zhikuan, J., Qingfang, H., 2015. Effects of straw mulch on soil water and winter wheat production in dryland farming. Sci. Rep. 5. https://doi.org/10.1038/srep10725.
199 Peng, Z., Wang, L., Xie, J., Li, L., Coulter, J.A., Zhang, R., Luo, Z., Cai, L., Carberry, P., Whitbread, A., 2020. Conservation tillage increases yield and precipitation use efficiency of wheat on the semi-arid Loess Plateau of China. Agric. Water Manag. 231. https://doi.org/10.1016/j.agwat.2020.106024.
200 Peng, Z., Wang, L., Xie, J., Li, L., Coulter, J.A., Zhang, R., Luo, Z., Kholova, J., Choudhary, S., 2019. Conservation Tillage Increases Water Use Efficiency of Spring Wheat by Optimizing Water Transfer in a Semi-Arid Environment. Agronomy-Basel. 9. https://doi.org/10.3390/agronomy9100583.
201 Rashid, M.A., Zhang, X., Andersen, M.N., Olesen, J.E., 2019. Can mulching of maize straw complement deficit irrigation to improve water use efficiency and productivity of winter wheat in North China Plain? Agric. Water Manag. 213, 1–11. https://doi.org/10.1016/j.agwat.2018.10.008.
202 Ren, Y.J., Gao, C., Yan, Z., Zong, R., Ma, Y., Li, Q., 2018. EFFECTS OF PLANTING SYSTEMS AND STRAW MULCHING ON CARBON EMISSIONS AND WINTER WHEAT GRAIN YIELD IN THE NORTH CHINA PLAIN. Exp Agric. 54, 520–530. https://doi.org/10.1017/S0014479717000217.
203 Rui, L., Chai, S.X., Chai, Y.W., Li. Y.W., Zhang, L., Cheng, H.B., 2022. Straw strip mulching: A sustainable technology for saving water and improving efficiency in dryland winter wheat production. J. Integr. Agric. 21, 3556–3568. https://doi.org/10.1017/s0014479717000217.
204 Sharma, P., Abrol, V., Sharma, R.K., 2011. Impact of tillage and mulch management on economics, energy requirement and crop performance in maize-wheat rotation in rainfed subhumid inceptisols, India. Eur. J. Agron. 34, 46–51. https://doi.org/10.1016/j.eja.2010.10.003.
205 Song, J., Bai, J., Zhang, Z., Yu, Q., Ren, G., Han, X., Wang, X., Ren, C., Feng, Y., Wang, X., 2024. How does straw returning combined with nitrogen fertilizer drive N2O emission in wheat-maize rotation system. Soil Use Manage. 40. https://doi.org/10.1111/sum.12988.
206 Tanveer, S.K., Zhang, J.L., Lu, X.L., Wen, X., Hai, J., Liu, Y., Hanjuan, Ali, M., Liao, Y., 2015. EFFECTS OF TILLAGE METHODS, CORN RESIDUE MULCH AND N FERTILIZER LEVELS ON THE WHEAT CROP PRODUCTIVITY UNDER THE RAIN FED CONDITION OF LOESS PLATEAU CHINA. Pak. J. Bot. 47, 1249–1258.
207 Wan, X., Zhao, L., Wang, Z., Che, L., Xu, Y., Zhou, Y., Shi, C., Li, L., Liu, Y., 2024. Effects of Straw Return and Nitrogen Application Rates on Soil Ammonia Volatilization and Yield of Winter Wheat. Agronomy-Basel. 14. https://doi.org/10.3390/agronomy14071469.
208 Wang, J., Fu, X., Sainju, U.M., Zhao, F., 2018. Soil carbon fractions in response to straw mulching in the Loess Plateau of China. Biol. Fertility. Soils. 54, 423–436. https://doi.org/10.1007/s00374-018-1271-z.
209 Wang, J., Ghimire, R., Fu, X., Sainju, U.M., Liu, W., 2018. Straw mulching increases precipitation storage rather than water use efficiency and dryland winter wheat yield. Agric. Water Manag. 206, 95–101. https://doi.org/10.1016/j.agwat.2018.05.004.
210 Wang, L., Li, L., Xie, J., Luo, Z., Zhang, R., Cai, L., Coulter, J.A., Palta, J.A., 2021. Managing the trade-offs among yield, economic benefits and carbon and nitrogen footprints of wheat cropping in a semi-arid region of China. Sci. Total Environ. 768. https://doi.org/10.1016/j.scitotenv.2021.145280.
211 Wang, L., Ma, L., Li, Y., Geilfus, C.M., Wei, J., Zheng, F., Liu, Z., Tan, D., 2023. Managing nitrogen for sustainable crop production with reduced hydrological nitrogen losses under a winter wheat-summer maize rotation system: an eight-season field study. Front. Plant Sci. 14. https://doi.org/10.3389/fpls.2023.1274943.
212 Wang, L., Yuan, X., Liu, C., Li, Z., Chen, F., Li, S., Wu, L., Liu, Y., 2019. Soil C and N dynamics and hydrological processes in a maize-wheat rotation field subjected to different tillage and straw management practices. Agric. Ecosyst. Environ. 285. https://doi.org/10.1016/j.agee.2019.106616.
213 Wang, W., Akhtar, K., Ren, G., Yang, G., Feng, Y., Yuan, L., 2019. Impact of straw management on seasonal soil carbon dioxide emissions, soil water content, and temperature in a semi-arid region of China. Sci. Total Environ. 652, 471–482. https://doi.org/10.1016/j.scitotenv.2018.10.207.
214 Wu, G., Ling, J., Zhao, D.Q., Xu, Y.P., Liu, Z.X., Wen, Y., Zhou, S.L., 2022. Deep-injected straw incorporation improves subsoil fertility and crop productivity in a wheat-maize rotation system in the North China Plain. Field Crops Res. 286. https://doi.org/10.1016/j.fcr.2022.108612.
215 Wu, L.F., Li, B.B., Qin, Y., Gregorich, E., 2017. Soil CO_2_ emission and carbon budget of a wheat/maize annual double-cropped system in response to tillage and residue management in the North China Plain. Int. J. Agric. Sustain. 15, 253–263. https://doi.org/10.1080/14735903.2017.1288518.
216 Wu, X., Tang, Y., Li, C., McHugh, A.D., Liu, M., Li, M., Xiong, T., 2019. In Situ Maize Residue Mulch Improves the Water Use Efficiency and Yield of the Subsequent Wheat under a Strip Inter-Cropping System. Agron J. 111, 924–934. https://doi.org/10.2134/agronj2018.06.0374.
217 Xia, L., Wang, S., Yan, X., 2014. Effects of long-term straw incorporation on the net global warming potential and the net economic benefit in a rice-wheat cropping system in China. Agric. Ecosyst. Environ. 197, 118–127. https://doi.org/10.1016/j.agee.2014.08.001.
218 Xie, W., Wu, L., Zhang, Y., Wu, T., Li, X., Ouyang, Z., 2017. Effects of straw application on coastal saline topsoil salinity and wheat yield trend. Soil Tillage Res. 169, 1–6. https://doi.org/10.1016/j.still.2017.01.007.
219 Xu, C., Han, X., Ru, S., Cardenas, L., Rees, R.M., Wu, D., Wu, W., Meng, F., 2019. Crop straw incorporation interacts with N fertilizer on N_2O_ emissions in an intensively cropped farmland. Geoderma. 341, 129–137. https://doi.org/10.1016/j.geoderma.2019.01.014.
220 Xu, J., Han, H., Ning, T., Li, Z., Lal, R., 2019. Long-term effects of tillage and straw management on soil organic carbon, crop yield, and yield stability in a wheat-maize system. Field Crops Res. 233, 33–40. https://doi.org/10.1016/j.fcr.2018.12.016.
221 Yan, Q., Dong, F., Li, J., Duan, Z., Yang, F., Li, X., Lu, J., Li, F., 2019. Effects of maize straw-derived biochar application on soil temperature, water conditions and growth of winter wheat. Eur J. Soil Sci. 70, 1280–1289. https://doi.org/10.1111/ejss.12863.
222 Yan, Q., Yang, F., Dong, F., Lu, J., Li, F., Duan, Z., Zhang, J., Lou, G., 2018. Yield loss compensation effect and water use efficiency of winter wheat under double-blank row mulching and limited irrigation in northern China. Field Crops Res. 216, 63–74. https://doi.org/10.1016/j.fcr.2017.11.009.
223 Yang, C., Wang, X., Li, J., Zhang, G., Shu, H., Hu, W., Han, H., Liu, R., Guo, Z., 2024. Straw return increases crop production by improving soil organic carbon sequestration and soil aggregation in a long-term wheat- cotton cropping system. J. Integr. Agric. 23, 669–679. https://doi.org/10.1016/j.jia.2023.06.009.
224 Yang, H., Li, J., Wu, G., Huang, X., Fan, G., 2023. Maize straw mulching with no-tillage increases fertile spike and grain yield of dryland wheat by regulating root-soil interaction and nitrogen nutrition. Soil Tillage Res. 228. https://doi.org/10.1016/j.still.2023.105652.
225 Yang, H., Wu, G., Mo, P., Chen, S., Wang, S., Xiao, Y., Ma, H.A., Wen, T., Guo, X., Fan, G., 2020. The combined effects of maize straw mulch and no-tillage on grain yield and water and nitrogen use efficiency of dry-land winter wheat (Triticum aestivum L.). Soil Tillage Res. 197. https://doi.org/10.1016/j.still.2019.104485.
226 Yang, Y., Ding, J., Zhang, Y., Wu, J., Zhang, J., Pan, X., Gao, C., Wang, Y., He, F., 2018. Effects of tillage and mulching measures on soil moisture and temperature, photosynthetic characteristics and yield of winter wheat. Agric. Water Manag. 201, 299–308. https://doi.org/10.1016/j.agwat.2017.11.003.
227 Yang, Y., Liu, H., Wu, J., Zhang, S., Gao, C., Zhang, S., Tang, D.W.S., 2023. Soil enzyme activities, soil physical properties, photosynthetic physical characteristics and water use of winter wheat after long-term straw mulch and organic fertilizer application. Front. Plant Sci. 14. https://doi.org/10.3389/fpls.2023.1186376.
228 Yang, Y.M., Liu, X.J., Li, W.Q., Li, C.Z., 2006. Effect of different mulch materials on winter wheat production in desalinized soil in Heilongjiang region of North China. J. Zhejiang Univ., Sci. 7, 858–867. https://doi.org/10.1631/jzus.2006.B0858.
229 Yao, Z., Wang, Y., Wang, R., Wang, X., Wang, Y., Zheng, X., Liu, C., Zhu, B., Zhou, M., Liu, Y., Butterbach-Bahl, K., 2024. Long-term straw return to a wheat-maize system results in topsoil organic C saturation and increased yields while no stimulating or reducing yield-scaled N_2_O and NO emissions. Agric. For. Meteorol. 349. https://doi.org/10.1016/j.agrformet.2024.109937.
230 Yao, Z., Zheng, X., Wang, R., Xie, B., Butterbach-Bahl, K., Zhu, J., 2013. Nitrous oxide and methane fluxes from a rice-wheat crop rotation under wheat residue incorporation and no-tillage practices. Atmos Environ. 79, 641–649. https://doi.org/10.1016/j.atmosenv.2013.07.006.
231 Yeboah, S., Zhang, R., Cai, L., Song, M., Li, L., Xie, J., Luo, Z., Wu, J., Zhang, J., 2016. Greenhouse gas emissions in a spring wheat-field pea sequence under different tillage practices in semi-arid Northwest China. Nutr. Cycling Agroecosyst. 106, 77–91. https://doi.org/10.1007/s10705-016-9790-1.
232 Yin, W., Chai, Q., Fan, Z., Hu, F., Fan, H., Guo, Y., Zhao, C., Yu, A., 2022. Energy budgeting, carbon budgeting, and carbon footprints of straw and plastic film management for environmentally clean of wheat-maize intercropping system in northwestern China. Sci. Total Environ. 826. https://doi.org/10.1016/j.scitotenv.2022.154220.
233 Yin, W., Fan, Z.L., Hu, F.L., Fan, H., He, W., Zhao, C., Yu, A.Z., Chai, Q., 2023. No-tillage with straw mulching promotes wheat production via regulating soil drying-wetting status and reducing soil-air temperature variation at arid regions. Eur. J. Agron. 145. https://doi.org/10.1016/j.eja.2023.126778.
234 Yin, W., Fan, Z.L., Hu, F.L., Fan, H., He, W., Sun, Y.L., Wang, F., Zhao, C., Yu, A.Z., Chai, Q., 2023. No-tillage with straw mulching boosts wheat grain yield by improving the eco-physiological characteristics in arid regions. J. Integr. Agric. 22, 3416–3429. https://doi.org/10.1016/j.jia.2023.02.041.
235 Yin, W., Guo, Y., Hu, F., Fan, Z., Feng, F., Zhao, C., Yu, A., Chai, Q., 2018. Wheat-Maize lntercropping With Reduced Tillage and Straw Retention: A Step Towards Enhancing Economic and Environmental Benefits in Arid Areas. Front. Plant Sci. 9. https://doi.org/10.3389/fpls.2018.01328.
236 Bai, Y.L., W, L., L. Y.L., Y, L.P., Y., Zhou. L.P., Ni, P., Cheng, M.F., 2015. Effects of long-term full straw return on yield and potassium response in wheat-maize rotation. J. Integr. Agric. 14, 2467–2476. https://doi.org/10.1016/s2095-3119(15)61216-3.
237 Yu, J., Hou, P., Gao, Q., Tan, Q., Jiang, D., Dai, T., Tian, Z., 2024. Optimizing nitrogen fertilizer and straw management promote root extension and nitrogen uptake to improve grain yield and nitrogen use efficiency of winter wheat (Triticum aestivum L.). Arch. Agron. Soil Sci. 70, 1–17. https://doi.org/10.1080/03650340.2024.2316232.
238 Zhang, L., Zheng, J.C., Chen, L.G., Shen, M.X., Zhang, X., Zhang, M.Q., Bian, X.M., Zhang, J., Zhang, W.J., 2015. Integrative effects of soil tillage and straw management on crop yields and greenhouse gas emissions in a rice-wheat cropping system. Eur. J. Agron. 63, 47–54. https://doi.org/10.1016/j.eja.2014.11.005.
239 Zhang, X., Wang, J., Feng, X., Yang, H., Li, Y., Yakov, K., Liu, S., Li, F.M., 2023. Effects of tillage on soil organic carbon and crop yield under straw return. Agric. Ecosyst. Environ. 354. https://doi.org/10.1016/j.agee.2023.108543.
240 Zhang, Y., Wang, J., Gong, S., Xu, D., Mo, Y., Zhang, B., 2021. Straw mulching improves soil water content, increases flag leaf photosynthetic parameters and maintaines the yield of winter wheat with different irrigation amounts. Agric. Water Manag. 249. https://doi.org/10.1016/j.agwat.2021.106809.
241 Zhang, Z., Zhang, Z., Feng, G., Lu, P., Huang, M., Zhao, X., 2022. Biochar Amendment Combined with Straw Mulching Increases Winter Wheat Yield by Optimizing Soil Water-Salt Condition under Saline Irrigation. Agriculture-Basel. 12. https://doi.org/10.3390/agriculture12101681.
242 Zhang, Z.S., Guo, L.J., Liu, T.Q., Li, C.F., Cao, C.G., 2015. Effects of tillage practices and straw returning methods on greenhouse gas emissions and net ecosystem economic budget in rice wheat cropping systems in central China. Atmos Environ. 122, 636–644. https://doi.org/10.3390/agriculture12101681.
243 Zhao, G., Wu, P., Liu, F., Li, S., Zhang, J., Dang, Y., Wang, L., Wang, S., Cheng, W., Cai, T., Fan, T., 2023. Plow layer management during the fallow season can enhance the wheat productivity and resource utilization in a semi-arid region. Soil Tillage Res. 228. https://doi.org/10.1016/j.still.2022.105633.
244 Zhao, K., Huang, M., Li, Y., Wu, J., Tian, W., Li, J., Hou, Y., Wu, S., Zhang, J., Zhang, Z., Zhao, Z., Ahammed, G.J., Li, G., 2023. Combined Organic Fertilizer and Straw Return Enhanced Summer Maize Productivity and Optimized Soil Nitrate-N distribution in Rainfed Summer Maize-Winter Wheat Rotation on the Southeast Loess Plateau. J. Soil Sci. Plant Nutr. 23, 938–952. https://doi.org/10.1007/s42729-022-01094-2.
245 Zhao, N., Bai, L., Han, D., Yao, Z., Liu, X., Hao, Y., Chen, Z., Zhang, X., Zhang, D., Jin, X., Wang, Z., 2024. Combined Application of Leguminous Green Manure and Straw Determined Grain Yield and Nutrient Use Efficiency in Wheat-Maize-Sunflower Rotations System in Northwest China. Plants-Basel. 13. https://doi.org/10.3390/plants13101358.
246 Zhao, Y., Wang, P., Li, J., Chen, Y., Ying, X., Liu, S., 2009. The effects of two organic manures on soil properties and crop yields on a temperate calcareous soil under a wheat-maize cropping system. J. Environ. Sci. 31, 36–42. https://doi.org/10.1016/j.eja.2009.03.001.
247 Zhao, Z., Li, M., Wu, Q., Zhang, Y., 2022. Effects of Different Soil Moisture-Holding Strategies on Growth Characteristics, Yield and Quality of Winter-Seeded Spring Wheat. Agronomy-Basel. 12. https://doi.org/10.3390/agronomy12112746.

**A.2 A list of 138 papers from which the data were extracted for wheat yield, WP and PFPN of biochar application (BA).**

1. Chen, Z., Jin, P.H., Hu, T.L., 2023. Effects of long-term successive biochar amendment on soil fertility, wheat production and microbial community structure. Soils. 55(6), 1230–1236. https://doi.org/10.13758/j.cnki.tr.2023.06.009. (in Chinese with English abstract).
2. He. J.M., Li, X.M., Wei, Y.P., Guo, H.Z., Yang, K.N., Sun, L.K., Li, X.Y., Jia, X.C., Li, Y.X., Li, R.F., Wang, Q., 2024. Effects of long-term deep tillage and straw return with biochar addition to lime concretion black soil on the aggregates and wheat-maize yield. Trans. Chin. Soc. Agric. Mach. 40(7), 1–11. https://doi.org/[10.11975/j.issn.1002-6819.202402001](https://doi.org/10.11975/j.issn.1002-6819.202402001). (in Chinese with English abstract).
3. Pan, Z.D., Cai, X.M., Cai, L.Q., et al., 2023. Effects of field-aged biochar on soil organic nitrogen fractions in dry farmland of Loess Plateau. J. Plant Nutr. Fert. 29(12), 2219–2231. https://doi.org/10.11674/zwyf.2023195. (in Chinese with English abstract).
4. Liang, M., Li, P., Zhao, L.H., Fan, Z.L., Hu, F.L., Fan, H., He, W., Chai, Q., Yin, W., 2025. Effect of soil conditioner and slow-release nitrogen fertilizer on dry matter accumulation and yield of wheat. Acta Agron. Sin. 51(2), 470–484. https://doi.org/10.3724/SP.J.1006.2025.41046. (in Chinese with English abstract).
5. Feng, Z.Z., Ye, Z.J., Zhai, W.L., et al., 2025. Effects of long-term application of biochar on physicochemical properties and wheat yield in fluvo-aquic soil. J. Henan Agric. Univ. 59(1), 49–56. https://doi.org/10.16445/j. (in Chinese with English abstract).
6. Dong, Y.J., Huyan, Y.J., Wang, J.P., Han, J., 2022. Effect of optimized fertilization on yield and nitrogen utilization of winter wheat in Guanzhong irrigation area. Acta Agric Boreali-Sinica. 31(3), 270–278. https://doi.org/10.12357/cjea.20220606. (in Chinese with English abstract).
7. Gao, S.J., Liu, X.R., Li, Y.C., Liu, X.W., 2024. Effects of biochar and straw return on greenhouse gas emissions and global warming potential in the farmland. Sci. Agric. Sin. 57(5), 935–949. https://doi.org/10.3864/j.issn.0578-1752.2024.05.009. (in Chinese with English abstract).
8. Tang, Y.J., Wu, Y., Qiao, Y.F., Ma, Y.Z., Miao, S.J., 2024. Evaluation of greenhouse gas emissions and economic benefits of different measures of rice-wheat rotation under warming. J. Agro-Environ. Sci. 43(2), 1–15. https://doi.org/10.11654/jaes.2024-1088. (in Chinese with English abstract).
9. Lu, H.Y., Du, W.T., Zhang, H.T., Ayaz, M., Xu, J.X., Wang, R.J., Li, R.N., Yang, X.Y., Zhang, S.L., 2021. Responses of crop yield, nitrogen use efficiency and nitrogen leaching to water nutrient management and biochar application. Acta Agric. Boreali-Occident. Sin. 49(3), 75–85. https://doi.org/0.13207/j.cnki.jnwafu.2021.03.009. (in Chinese with English abstract).
10. Liao, X., Liu, D.Y., Chen, Z.M., et al., 2021. Effects of field-aged biochar on NH3 emissions under wheat-maize rotation. J. Agro-Environ. Sci. 40(6), 1326–1336. https://doi.org/10.11654/jaes.2020-1414.(in Chinese with English abstract).
11. Wu, Y., Qiao, Y.F., Jin, L.H., Miao, S.J., Wu, X.Y., Ma, Y.Z., Yu, J., 2024. Effects of warming and its countermeasures on wheat photosynthetic characteristics and enzyme activities. Agricultural Research in the Arid Areas. 42(4), 42–51. https://doi.org/[10.7606/j.issn.1000-7601.2024.04.05](https://doi.org/10.7606/j.issn.1000-7601.2024.04.05). (in Chinese with English abstract).
12. Zhou, J.S., Zheng, J.W., Chi, Z.Z., Liu, X.Y., Pan, G.X., Zheng, J.F., Li, L.Q., Zhang, X.H., Cheng, K., 2016. Effects of biochar amendment on crop yield and the uptake of nitrogen, phosphorus and potassium. J. Nanjing Agric. Univ. 39(5), 791–799. https://doi.org/10.7685/jnau.201602022. (in Chinese with English abstract).
13. Dong, Y.B., Wu, Z., Li, B., Xu, X., Xiong, Z.Q., 2017. Effects of biochar reapplication on ammonia volatilization and nitrogen use efficiency during wheat season in a rice-wheat annual rotation system. J. Plant Nutr. Fert. 23(5), 1258–1267. https://doi.org/10.11674/zwyf.17060. (in Chinese with English abstract).
14. Zhang, X., Zheng, C.Y., Li, S.M., Xie, F.J., Deng, A.X., Zhang, J., Song, Z.W., Zhang, W.J., 2020. Long-term combined application of chemical and organic fertilizers decrease net greenhouse gas emission in wheat-soybean system in Huang-Huai-Hai region. J. Plant Nutr. Fert. 26(12), 2204–2215. https://doi.org/10.11674/zwyf.20392. (in Chinese with English abstract).
15. Liu, M.L., Liu, Y., Chen, T.T., Zou, J., Shi, W.Q., Zeng, F.S., Gong, S.J., Huang, Z.Y., Liu, W., Yang, L.J., Yu, D.Z., 2021. Rice straw biochar: Effects on nutrient absorption and yield of wheat in Jianghan Plain. Chin. Agric. Sci. Bull. 37(15), 7–13. https://doi.org/10.11924/j.issn.1000-6850.casb2020-0396. (in Chinese with English abstract).
16. Chen, C.X., Zhang, Y.Q., Nie, S.H., Kong, D.P., Sailihan, S., Xu, Q.J., Lei, J.J., 2023. Effects of biomass charcoal application rate on the growth, development, and yield of winter wheat under drip irrigation. Xinjiang Agric. Sci. 60(9), 2146–2151. https://doi.org/10.6048/j.issn.1001-4330.2023.09.008. (in Chinese with English abstract).
17. Dang, H.Y., Hu, B., Feng, H.L., Zhu, S.M., Li, M.H., Du, Z.N., Zhou, J.Y., 2025. Effect of biochar application on winter yield and soil nutrient. Crop Res. 1, 16–22. (in Chinese with English abstract).
18. Qiu, L.L., Li, Z.Q., Xu, J.S., Li, H., Zhao, B.Z., Zhang, J.B., 2021. Effects of biochar and straw application on soil biochemical properties and wheat yield in yellow-cinnamon soil. Soils. 53(3), 475–482. https://doi.org/[10.13758/j.cnki.tr.2021.03.005](https://doi.org/10.13758/j.cnki.tr.2021.03.005). (in Chinese with English abstract).
19. Kan, Z.R., Ma, S.T., Qi, J.Y., Pu, C., Wang, X., Zhao, X., Zhang, H.L., 2019. Effect of biochar addition on photosynthetic potential and grain yield of winter wheat. J. Triticeae Crops. 39(6), 719–727. https://doi.org/[10.7606/j.issn.1009-1041.2019.06.13](https://doi.org/10.7606/j.issn.1009-1041.2019.06.13). (in Chinese with English abstract).
20. Hui, C., Yang, W.J., Song, S.L., Chen, Y.X., Zhang, J.S., Jia, Y.H., Wei, H.P., Xu, W.L., 2022. Effects of biochar application on mechanical stability of soil aggregates and yield of spring wheat. Chin. J. Soil Sci. 53(2), 349–355. https://doi.org/10.19336/j.cnki.trtb.2021061201. (in Chinese with English abstract).
21. Sun, H.N., Wang, S.W., Li, Y.L., Yang, W.J., Yin, X.S., Yin, L.N., Deng, X.P., 2018. Effects of biochar levels on winter wheat yield and water-use efficiency in Loess Plateau. Agricultural Research in the Arid Areas. 36(6), 159–167. https://doi.org/10.7606/j.issn.1000-7601.2018.06.24. (in Chinese with English abstract).
22. Guo, S., Yang, W.J., Li, T.Y., Yang, M., Chen, Y.X., Song, S.L., Hui, C., Geng, H.W., 2024. Comprehensive evaluation of biochar amount and irrigation amount on grain yield and quality of spring wheat in irrigation district of northern Xinjiang. J. Triticeae Crops. 44(3), 1–9. https://doi.org/10.7606/j.issn.1009-1041.2024.05.12. (in Chinese with English abstract).
23. Yang, M., Yang, W.J., Gao, W.C., Jia, Y.H., Zhang, J.S., 2023. Effects of combined application of biochar and nitrogen fertilizer on dry matter transport, agronomic characteristics and yield of winter wheat in irrigation area. Crops. 5, 138–144. https://doi.org/10.16035/j.issn.1001-7283.2023.05.020. (in Chinese with English abstract).
24. Li, Z.Y., Qi, X.B., Fan, X.Y., Wu, H.Q., Du, Z.J., Li, P., Lü, M.C., 2015. Influences of biochars on growth, yield, water use efficiency and root morphology of winter wheat. Trans. Chin. Soc. Agric. Mach. 31(12), 119–124. https://doi.org/10.11975/j.issn.1002-6819.2015.12.016. (in Chinese with English abstract).
25. Yao, Q., Yu, R.H., Zhang, H.Y., Qin, K., Zhang, P., Peng, Z.P., Wang, Y., Wang, D.W., 2022. Effects of biochar application on soil nutrients and crop yield in winter wheat farmland. Soil Fert. Sci. China. 3, 68–74. https://doi.org/10.11838/sfsc.1673-6257.20684. (in Chinese with English abstract).
26. Kan, Z.R., Pu, C., Qi, J.Y., Ma, S.T., Liu, P., Zhao, X., Zhang, H.L., 2019. Effects of biochar on soil water and grain yield of winter wheat in the North China Plain. J. China Agric. Univ. 24(4), 1–10. https://doi.org/10.11841/j.issn.1007-4333.2019.04.01. (in Chinese with English abstract).
27. Fu, W., Fan, J., Hu, Y.T., Zhao, J., Hao, M.D., 2023. Effects of fertilization and film mulching on soil physical and chemical properties and winter wheat yield on the Loess Plateau. J. Plant Nutr. Fert. 29(5), 1–10. https://doi.org/10.11674/zwyf.16486. (in Chinese with English abstract).
28. Zhang, S.Q., Zhang, B., Yue, K., Huang, S.M., 2023. Effect of biochar on ammonia volatilization and nitrogen uptake in wheat cultivated fluvo-aquic soil. Acta Agric. Nucl. Sin. 37(11), 2258–2267. https://doi.org/10.11869/j. (in Chinese with English abstract).
29. Dong, F., Yan, Q.Y., Duan, Z.Q., Li, F., Lu, J.X., Yang, F., Li, X., Wang, M., Jia, Y.Q., 2020. Effects of biochar addition on yield and water use efficiency of winter wheat under different irrigation conditions. Acta Agric Boreali-Sinica. 35(1), 149–157. https://doi.org/CNKI:SUN:HBNB.0.2020-01-024. (in Chinese with English abstract).
30. Zhang, H., Li, J.H., Gao, L.X., Chu, G.X., Jia, Q.Q., He, K., 2015. Effects of biochar on yield of drip irrigation spring wheat and soil fertility. Soil Fert. Sci. China. 2, 55–60. https://doi.org/10. 11838/sfsc.20160210. (in Chinese with English abstract).
31. Li, C.Z., Zhang, H., Yao, W.J., Xu, C., Wu, D., Wang, J.D., Ai, Y.C., Zhang, Y.C., 2024. Effects of biochar application combined with nitrogen fertilizer on soil physicochemical properties and winter wheat yield in the typical ancient region of Yellow River, China. Chin. J. Appl. Ecol. 35(2), 1–9. https://doi.org/10.13287/j.1001-9332.202010.028. (in Chinese with English abstract).
32. Zhang, M., Wang, G.E., Li, Y.H., Zhao, Y., Zhang, Y.C., Guo, L., 2025. Effects of biochar combined with wood vinegar on salinity soil nutrients and salt tolerance of winter wheat. J. Agro-Environ. Sci. 44(5), 1334–1342. https://doi.org/[10.11654/jaes.2024-0400](https://doi.org/10.11654/jaes.2024-0400). (in Chinese with English abstract).
33. Chen, J., Zhang, J.G., Zhao, Y., Zhang, A.F., Cheng, G., 2018. Effects of straw and biochar amendment on greenhouse gases emission in wheat-maize rotation cropland in Guanzhong area. Res. Soil Water Conserv. 25(5), 170–178. https://doi.org/10.13869/j.cnki.rswc.2018.05.024. (in Chinese with English abstract).
34. Yang, W.J., Yang, M., Guo, S., Song, S.L., Chen, Y.X., Wang, S., Zhao, H.M., 2024. Effects of reducing nitrogen fertilizer combined with biochar on the yield and quality of wheat and carbon sequestration and emission reduction in Northern Xinjiang of China. Trans. Chin. Soc. Agric. Mach. 40(4), 104–111. https://doi.org/10.11975/j.issn.1002-6819.202307171. (in Chinese with English abstract).
35. Sun, L.Y., Han, Y., 2024. Effects of biochar on N2O emissions and wheat yield in coastal saline wheat field. J. Triticeae Crops. 44(6), 780–786. https://doi.org/10.7606/j.issn.1009-1041.2021.06.11. (in Chinese with English abstract).
36. Feng, Z., Chen, Y.X., Wang, Y.L., Lin, Y., Wang, Q., Zhang, T.T., Geng, Z.C., 2017. Effect of biochar on CH4 and N2O emissions from Lou soil. J. Environ. Sci. 38(9), 3831–3839. https://doi.org/10.13227/j.hjkx.201612210. (in Chinese with English abstract).
37. He, J.Q., Yang, W.J., Jia, Y.H., Li, Y.H., Xing, D.J., Hui, C., Gao, W.C., 2021. Effect of reducing phosphate fertilizer and applying biochar on phosphorus utilization and yield of spring wheat in Northern Xinjiang. Chin. Agric. Sci. Bull. 37(3), 13–19. https://doi.org/10.11924/j.issn.1000-6850.casb20200300265. (in Chinese with English abstract).
38. Cheng, G., Chen, J., Liu, J.J., Zhang, A.F., Wang, X.D., Feng, H., Zhao, Y., 2017. Comparative analysis on effect of wheat straw and its biochar amendment on net global warming potential under wheat-maize rotation ecosystem in the Guanzhong Plain. J. Environ. Sci. 38(2), 792–801. https://doi.org/10.13227/j.hjkx.201607071. (in Chinese with English abstract).
39. Liu, Y., Khan, M.J., Jin, H.Y., Bai, X.Y., Xie, Y.X., Zhao, X., Wang, S.Q., Wang, C.Y., 2015. Effects of successive application of crop-straw biochar on crop yield and soil properties in cambosols. Acta Pedologica Sinica. 52(4), 849–858. https://doi.org/10.11766/trxb201412020611. (in Chinese with English abstract).
40. Liu, Z.W., Liu, J., Wu, J.S., Li, Y.F., Jiang, P.K., 2023. Effects of biochar and humic acid application on global warming potentials of CH4 and N2O in a rice-wheat rotation system. Trans. Chin. Soc. Agric. Mach. 39(11), 220–229. https://doi.org/10.11975/j.issn.1002-6819.202302028. (in Chinese with English abstract).
41. Liu, Y.J., Xie, Y.X., Dong, C., He, D.X., Ma, D.Y., Wang, C.Y., Guo, T.C., 2018. Effects of straw biochar application on grain yield of wheat and physicochemical properties in fluvio-aquatic soil. Acta Agric Boreali-Sinica. 33(3), 232–238. https://doi.org/10.7668/hbnxb.2018.03.034. (in Chinese with English abstract).
42. Liu, Y., Shi, W.Q., Zeng, F.S., Liu, M.L., Chen, T.T., Huang, Z.Y., Xue, M.F., Xiang, L.B., Gong, S.J., Yang, L.J., Yu, D.Z., 2020. Effect of biochar on controlling wheat Fusarium head blight and yield. Plant Protection. 46(4), 270–274. https://doi.org/10.16688/j.zwbh.2019506. (in Chinese with English abstract).
43. Lü, M.F., Ma, X.C., Cai, T., Jia, Z.K., 2022. Effects of biochar application on soil hydrothermal environment and soil respiration in winter wheat field. Agricultural Research in the Arid Areas. 40(3), 197–206. https://doi.org/10.606/j.issn.1000-7601.2022.03.24. (in Chinese with English abstract).
44. Zhang, Q.R., Liu, B., Xie, Y.H., Li, T.L., Li, S., Jiang, L.W., Dou, L., Liu, Y.F., 2021. Effects of straw carbon and silicon-potassium fertilizers on soil organic carbon components and wheat yield in dryland. Journal of Shanxi Agricultural Sciences. 49(3), 332–337. https://doi.org/10.3969/j.issn.1002-2481.2021.03.14. (in Chinese with English abstract).
45. Fu, M.X., Wu, M.Y., Han, B.B., Zhang, Q., Han, Y.L., Tan, J.F., Li, P.P., Zhang, T., Li, H., 2023. Effects of straw returning and biochar application on yield and nitrogen use efficiency of winter wheat. J. Plant Nutr. Fert. 29(4), 1–8. https://doi.org/10.11924/j.issn.1000-6850.casb2020-0732. (in Chinese with English abstract).
46. Li, T., Wang, X.G., Hu, T.X., et al., 2016. Impact of biochar on NO emission from cropland of purple soil. Soils. 48(5), 879–886. https://doi.org/10.13758/j.cnki.tr.2016.05.007. (in Chinese with English abstract).
47. Li, H., Zhen, Y.M., Zhang, Z.X., Wang, Y.Q., Fu, S., Liu, X.M., Zhen, W.C., Men, M.X., Peng, Z.P., 2022. Effects of nitrogen reduction combined with organic matter on soil properties and wheat yield. J. Soil Water Conserv. 36(2), 166–172. https://doi.org/10.13870/j.cnki.stbcxb.2022.02.022. (in Chinese with English abstract).
48. Zhao, L.N., Zhang, L.Y., Meng, X.R., Guo, S., Yang, M., Ma, L., Yang, W.J., 2023. Effects of different amounts of nitrogen fertilizer and appropriate amount of biochar on photosynthesis, dry matter accumulation and yield of spring wheat. Southwest China Journal of Agricultural Sciences. 36(5), 1–11. https://doi.org/10.16213/j.cnki.scjas.2024.6.005. (in Chinese with English abstract).
49. Li, C.Z., Zhang, H., Yao, W.J., Xu, C., Wu, D., Wang, J.D., Ai, Y.C., Zhang, Y.C., 2020. Effects of biochar application combined with nitrogen fertilizer on soil physicochemical properties and winter wheat yield in the typical ancient region of Yellow River, China. Chin. J. Appl. Ecol. 31(10), 3424–3432. https://doi.org/10.13287/j.1001-9332.202010.028. (in Chinese with English abstract).
50. Yao, L.R., Li, W., Zhu, Y.Z., Pubu, C.J., Han, J., 2023. Effects of biochar application on soil bacterial community diversity and winter wheat growth in wheat fields. J. Environ. Sci. 44(6), 3396–3407. https://doi.org/10.13227/j.hjkx.202207125. (in Chinese with English abstract).
51. Xu, H.W., Li, N., Feng, Y.Z., Ren, G.X., Xie, C.H., Lü, H.F., Ma, X.X., Hao, J.Q., 2020. Effects of nitrogen fertilizer and straw returning methods on N2O emissions in wheat-maize rotational soils. J. Environ. Sci. 41(12), 5668–5676. https://doi.org/10.13227/j.hjkx.202005151. (in Chinese with English abstract).
52. Yang, L., Xia, Y., Han, Z.Q., Wang, J., Song, H., Dong, Z.R., Che, Z., 2019. Effect of nitrogen regulation on nitrogen assimilation and yield of wheat. J. Triticeae Crops. 39(10), 1195–1201. https://doi.org/10.7606/j.issn.1009-1041.2019.10.08. (in Chinese with English abstract).
53. Li, P.P., Tong, H.T., Zhai, Q.H., Han, Y.L., Jiang, Y., 2019. Effects of water conservation measures on soil moisture, microbial biomass and wheat yield in a sandy fluvio-aquatic soil. Soil Fert. Sci. China. 4, 78–83. https://doi.org/10.11838/sfsc.1673-6257.18356. (in Chinese with English abstract).
54. Liu, J.Y., Qiu, H.S., Zhang, W.Z., Cai, J.M., Wang, X.S., Lü, M.C., 2021. The effects of irrigation and biochar amendment on yield and water and nitrogen use efficiency of winter wheat. Journal of Irrigation and Drainage. 40(6), 59–65. https://doi.org/10.13522/j.cnki.ggps.2020373. (in Chinese with English abstract).
55. Chen, Y.X., Yang, W.J., Zhao, H.M., Li, D.P., Song, S.L., Hui, C., Geng, H.W., 2024. Effects of nitrogen fertilizer and biochar on growth, yield and quality of spring wheat. J. Triticeae Crops. 44(1), 92–100. (in Chinese with English abstract).
56. Xie, Y.X., Liu, Y.J., Zhang, W.N., Dong, C., Zhao, X., He, D.X., Wang, C.Y., Guo, T.C., Wang, S.Q., 2018. Long-term application of biochar in fluvio-aquatic soil improving wheat yield and nitrogen utilization. Trans. Chin. Soc. Agric. Mach. 34(14), 115–123. https://doi.org/10.11975/j.issn.1002-6819.2018.14.01. (in Chinese with English abstract).
57. Yang, M., Zhao, H.M., Dilireba, X., Yang, W.J., Zhang, J.S., Hui, C., 2024. Effects of nitrogen fertilizer reduction and biochar application on population structure, photosynthetic characteristics and yield of spring wheat. Xinjiang Agricultural Sciences. 61(7), 1582–1589. https://doi.org/10.6048/j.issn.1001-4330.2024.07.003.(in Chinese with English abstract).
58. Li, C.Y., Lu, S.C., 2020. Combined application of biochar and alum in winter wheat-summer maize multiple cropping area in North China: Effects on soil carbon and nitrogen status. Chin. Agric. Sci. Bull. 36(18), 97–103. https://doi.org/[10.11924/j.issn.1000-6850.casb19030132](https://doi.org/10.11924/j.issn.1000-6850.casb19030132). (in Chinese with English abstract).
59. Shi, D.P., Ye, Z.Z., Li, J., Xu, Y.H., Lü, S.Q., Wang, L.Q., Zhou, C.J., 2023. Effects of nitrogen combined with biochar and urease inhibitor on yield and nitrogen utilization of summer maize-winter wheat system. Agricultural Research in the Arid Areas. 41(4), 51–60. https://doi.org/[10.7606/j.issn.1000-7601.2023.04.06](https://doi.org/10.7606/j.issn.1000-7601.2023.04.06). (in Chinese with English abstract).
60. Song, S.L., Yang, W.J., Chen, Y.X., Jia, Y.H., Li, D.P., Hui, C., Guo, S., Yang, M., 2022. Effect of reduced nitrogen fertilizer combined with biochar on photosynthetic characteristics, dry matter accumulation and distribution, and yield of spring wheat in irrigated area of Northern Xinjiang. J. Triticeae Crops. 43(3), 311–320. https://doi.org/10.7606/j.issn.1009-1041.2023.03.0. (in Chinese with English abstract).
61. Guo, S.Y., Shang, S., Tang, Q.N., Zhang, Y., Lu, G.Y., 2022. Effects of different rotation tillage methods and biochar on soil enzyme activity, soil nutrients and yield of wheat and maize. Crops. 3, 211–217. https://doi.org/10.16035/j.issn.1001-7283.2022.03.031. (in Chinese with English abstract).
62. Li, L., Zhou, Z.Q., Pan, X.J., Xiong, Z.Q., 2015. Effects of biochar on N2O and CH4 emissions from paddy field under rice-wheat rotation during rice and wheat growing seasons relative to timing of amendment. Acta Pedologica Sinica. 52(4), 839–848. https://doi.org/10.11766/trxb201407160357. (in Chinese with English abstract).
63. Feng, X.P., Yu, L., Han, Z.Q., Dai, X., Dong, X., Dong, Z.R., Che, Z., 2023. Effects of nitrogen regulation on N2O emission and yield of wheat-maize double cropping system. J. Yunnan Agric. Univ., Nat. Sci. 38(1), 9–17. https://doi.org/10.12101/j.issn.1004-390X(n).202205020. (in Chinese with English abstract).
64. Zheng, Y.Z., Nie, C., Tian, X.F., Zhai, S., Sun, S.C., 2022. Response of winter wheat yield and water use efficiency to biochar and straw returning. Water Saving Irrigation. 3, 20–26. (in Chinese with English abstract).
65. Hen, Y.X., Yang, M., Ma, A.J., Hui, C., Deng, T.C., Yang, W.J., 2022. Effects of nitrogen fertilizer with biochar on soil nutrient changes and winter wheat yield in fields. J. Xinjiang Agric. Univ. 45(4), 292–297. (in Chinese with English abstract).
66. Cheng, Y., Ma, D., Zhao, J., Zhang, Q., Li, X., Zhao, Y., Zheng, W., Zhang, B., Liu, Z., 2025. Biochar application does not improve crop growth and yield in a semi-humid region in the HuangHuaiHai Plain of China: A 7–year consecutive field experiment. Soil Tillage Res. 247, 106367. https://doi.org/10.1016/j.still.2024.106367.
67. Yang, W., Wang, Z., Zhao, H., Li, D., Jia, H., Xu, W., 2024. Biochar application influences the stability of soil aggregates and wheat yields. Plant Soil Environ. 70, 125–141. https://doi.org/10.17221/199/2023-pse.
68. Wu, W., Chai, H., Gao, P., Gao, P., Zhang, X., Li, M., Guo, X., Lv, Q., 2024. Effect of biochar addition on water–salt distribution and growth of winter wheat in coastal saline‐alkali soils under brackish water irrigation. Soil Use Manage. 40, 13028. https://doi.org/10.1111/sum.13028.
69. Wu, H., Wang, D., Zhang, D., Rao, W., Yuan, Q., Shen, X., Ma, G., Jie, X., Liu, S., 2024. Responses of N2O, CO2, and NH3 Emissions to Biochar and Nitrification Inhibitors Under a Delayed Nitrogen Application Regime. Agriculture. 14, 1986. https://doi.org/10.3390/agriculture14111986.
70. Wang, H., Fan, J., Shao, M.a., Wang, X., Wang, J., Hao, M., 2024. Effects of biochar versus film mulching on soil hydrothermal properties and wheat crop performance in semi‐arid loess. Eur. J. Soil Sci. 75, 13497. https://doi.org/10.1111/ejss.13497.
71. Elbagory, M., Shaker, E.M., El-Nahrawy, S., Omara, A.E.-D., Khalifa, T.H., 2024. The Concurrent Application of Phosphogypsum and Modified Biochar as Soil Amendments Influence Sandy Soil Quality and Wheat Productivity. Plants. 13, 1492. https://doi.org/10.3390/plants13111492.
72. Du, M., Zhang, J., Chen, Q., Lin, Y., Yan, H., Liu, C., Wang, Z., Wang, G., 2024. Effects of straw application on soil hydrothermal conditions and crop yield in a maize and wheat rotation system. Hydrol. Processes. 38, 15252. https://doi.org/10.1002/hyp.15252.
73. Blanco-Canqui, H., Creech, C.F., Easterly, A.C., 2024. How does biochar impact soils and crops in a semi-arid environment? A 5-year assessment. Field Crops Res. 310, 109340. https://doi.org/10.1016/j.fcr.2024.109340.
74. Aziz, M.A., Khan, K.S., Khalid, R., Shabaan, M., Alghamdi, A.G., Alasmary, Z., Majrashi, M.A., 2024. Integrated application of biochar and chemical fertilizers improves wheat (Triticum aestivum) productivity by enhancing soil microbial activities. Plant Soil. 502, 433–448. https://doi.org/10.1007/s11104-024-06556-3.
75. Zhu, X., Chen, L., Kong, X., Bao, S., Wu, S., Fang, L., Shen, Y., 2023. Biochar alters the morphology of plant roots to enable optimized and reduced nitrogen fertilizer applications. Plant Soil. 492, 655–673. https://doi.org/10.1007/s11104-023-06154-9.
76. Yang, W., Wang, Z., Guo, S., Yang, M., Zhao, L., Zhao, H., Jia, H., Xu, W., 2023. Evaluation of Soil Fertility Quality under Biochar Combined with Nitrogen in an Irrigated Wheat Field in Northern Xinjiang, China. Agron. 13, 2518. https://doi.org/10.3390/agronomy13102518.
77. Wu, Z., Dong, Y., Zhang, X., Xu, X., Xiong, Z., 2023. Biochar single application and reapplication decreased soil greenhouse gas and nitrogen oxide emissions from rice–wheat rotation: A three-year field observation. Geoderma. 435, 116498. https://doi.org/10.1016/j.geoderma.2023.116498.
78. Sarwar, N., Abbas, N., Farooq, O., Akram, M., Hassan, M.W., Mubeen, K., Rehman, A.U., Shehzad, M., Ahmad, M., Khaliq, A., 2023. Biochar Integrated Nutrient Application Improves Crop Productivity, Sustainability and Profitability of Maize–Wheat Cropping System. Sustain. 15, 2232. https://doi.org/10.3390/su15032232.
79. Liu, Y., Zhou, X., Du, C., Liu, Y., Xu, X., Ejaz, I., Hu, N., Zhao, X., Zhang, Y., Wang, Z., Sun, Z., 2023. Trade-off between soil carbon emission and sequestration for winter wheat under reduced irrigation: The role of soil amendments. Agric. Ecosyst Environ. 352, 108535. https://doi.org/10.1016/j.agee.2023.108535.
80. Liu, X., Yang, J., Tao, J., Yao, R., Li, W., Xie, W., Wang, X., 2023. Effects of the combined application of biochar and humic substances on the improvement of saline cropland in the Yellow River Delta of China. Land Degrad. Dev. 34, 4793–4809. https://doi.org/10.1002/ldr.4810.
81. Han, S., Li, H., Rengel, Z., Du, Z., Hu, N., Wang, Y., Zhang, A., 2023. Biochar application promotes crops yield through regulating root development and the community structure of root endophytic fungi in wheat-maize rotation. Soil Tillage Res. 234, 105827. https://doi.org/10.1016/j.still.2023.105827.
82. Ghazouani, H., Ibrahimi, K., Amami, R., Helaoui, S., Boughattas, I., Kanzari, S., Milham, P., Ansar, S., Sher, F., 2023. Integrative effect of activated biochar to reduce water stress impact and enhance antioxidant capacity in crops. Sci. Total Environ. 905, 166950. https://doi.org/10.1016/j.scitotenv.2023.166950.
83. Gao, S., Peng, Q., Liu, X., Xu, C., 2023. The Effect of Biochar and Straw Return on N2O Emissions and Crop Yield: A Three-Year Field Experiment. Agriculture. 13, 2091. https://doi.org/10.3390/agriculture13112091.
84. Baldoni, N., Francioni, M., Trozzo, L., Toderi, M., Fornasier, F., D'Ottavio, P., Corti, G., Cocco, S., 2023. Effect of wood gasification biochar on soil physicochemical properties and enzyme activities, and on crop yield in a wheat-production system with sub-alkaline soil. Biomass Bioenergy. 176, 106914. https://doi.org/10.1016/j.biombioe.2023.106914.
85. Bai, J., Song, J., Chen, D., Zhang, Z., Yu, Q., Ren, G., Han, X., Wang, X., Ren, C., Yang, G., Wang, X., Feng, Y., 2023. Biochar combined with N fertilization and straw return in wheat-maize agroecosystem: Key practices to enhance crop yields and minimize carbon and nitrogen footprints. Agric. Ecosyst Environ. 347, 108366. https://doi.org/10.1016/j.agee.2023.108366.
86. Wang, S., Gao, P., Zhang, Q., Shi, Y., Guo, X., Lv, Q., Wu, W., Zhang, X., Li, M., Meng, Q., 2022. Biochar improves soil quality and wheat yield in saline-alkali soils beyond organic fertilizer in a 3-year field trial. Environ. Sci. Pollut. Res. 30, 19097–19110. https://doi.org/10.1007/s11356-022-23499-3.
87. Sun, Y., Chen, X., Yang, J., Luo, Y., Yao, R., Wang, X., Xie, W., Zhang, X., 2022. Biochar Effects Coastal Saline Soil and Improves Crop Yields in a Maize-Barley Rotation System in the Tidal Flat Reclamation Zone, China. Water. 14, 3204. https://doi.org/10.3390/w14203204.
88. Nocentini, M., Mastrolonardo, G., Panettieri, M., Vignolini, P., Romani, A., Criscuoli, I., Knicker, H., Certini, G., 2022. Adding Biochar to a Fertile Temperate Soil Has No Impact on the Growth and Very Little on the Quality of Sunflower (Helianthus annuus L.) and Ancient and Modern Wheat Varieties (Triticum spp.). J. Soil Sci. Plant Nutr. 23, 953–964. https://doi.org/10.1007/s42729-022-01095-1.
89. Li, Y., Feng, H., Chen, J., Lu, J., Wu, W., Liu, X., Li, C., Dong, Q.g., Siddique, K.H.M., 2022. Biochar incorporation increases winter wheat (Triticum aestivum L.) production with significantly improving soil enzyme activities at jointing stage. Catena. 211, 105979. https://doi.org/10.1016/j.catena.2021.105979.
90. Khan, M.A., Basir, A., Fahad, S., Adnan, M., Saleem, M.H., Iqbal, A., Amanullah, Al-Huqail, A.A., Alosaimi, A.A., Saud, S., Liu, K., Harrison, M.T., Nawaz, T., 2022. Biochar Optimizes Wheat Quality, Yield, and Nitrogen Acquisition in Low Fertile Calcareous Soil Treated With Organic and Mineral Nitrogen Fertilizers. Front. Plant Sci. 13, 879788. https://doi.org/10.3389/fpls.2022.879788.
91. El-Sharkawy, M., El-Naggar, A.H., Al-Huqail, A.A., Ghoneim, A.M., 2022. Acid-Modified Biochar Impacts on Soil Properties and Biochemical Characteristics of Crops Grown in Saline-Sodic Soils. Sustain. 14, 8190. https://doi.org/10.3390/su14138190.
92. Dong, L., Yang, X., Shi, L., Shen, Y., Wang, L., Wang, J., Li, C., Zhang, H., 2022. Biochar and nitrogen fertilizer co-application changed SOC content and fraction composition in Huang-Huai-Hai plain, China. Chemosphere. 291, 132925. https://doi.org/10.1016/j.chemosphere.2021.132925.
93. Cong, 2022. Effects of Biochar Application and Irrigation on Soil Preferential Flow and Winter Wheat Productivity. Pol. J. Environ. 31, 625–635. https://doi.org/10.15244/pjoes/139103.
94. Xie, Y., Dong, C., Chen, Z., Liu, Y., Zhang, Y., Gou, P., Zhao, X., Ma, D., Kang, G., Wang, C., Zhu, Y., Guo, T., 2021. Successive biochar amendment affected crop yield by regulating soil nitrogen functional microbes in wheat-maize rotation farmland. Environ Res. 194, 110671. https://doi.org/10.1016/j.envres.2020.110671.
95. Wei, W., Liu, S., Cui, D., Ding, X., 2021. Interaction between nitrogen fertilizer and biochar fertilization on crop yield and soil chemical quality in a temperate region. J. Agric. Sci. 159, 106–115. https://doi.org/10.1017/s0021859621000277.
96. Wei, S., Bian, R.J., Li, L.Q., Lian, W.L., Liu, X.Y., Zheng, J.F., Cheng, K., Zhang, X.H., Drosos, M., Joseph, S., Pan, G.X., 2021. Assessing the impacts of biochar-blended urea on nitrogen use efficiency and soil retention in wheat production. GCB bioenergy. 159, 106–115. https://doi.org/10.1111/gcbb.12904.
97. Mian, I.A., Ahmad, B., Khan, S., Khan, B., Dawar, K., Tariq, M., Mussarat, M., Muhammad, M.W., Ali, S., Bibi, H., Muhammad, F., Khan, K., 2021. Improving wheat productivity and soil quality through integrated phosphorous management with residual effect of biochar. Journal of Saudi Chemical Society. 25, 101175. https://doi.org/10.1016/j.jscs.2020.11.008.
98. Mahmoud, E.K., Ibrahim, M., Khader, A., Solaiman, Z., 2021. Phosphorus fertilisation and biochar impacts on soil fertility and wheat. Crop Pasture Sci. 74, 21–30. https://doi.org/10.1071/cp21095.
99. Korai, P.K., Sial, T.A., Pan, G., Abdelrahman, H., Sikdar, A., Kumbhar, F., Channa, S.A., Ali, E.F., Zhang, J., Rinklebe, J., Shaheen, S.M., 2021. Wheat and maize-derived water-washed and unwashed biochar improved the nutrients phytoavailability and the grain and straw yield of rice and wheat: A field trial for sustainable management of paddy soils. J. Environ Manage. 297, 113250. https://doi.org/10.1016/j.jenvman.2021.113250.
100. Hu, Y.J., Sun, B.H., Wu, S.F., Feng, H., Gao, M.X., Zhang, B.B., Liu, Y.Y., 2021. After-effects of straw and straw-derived biochar application on crop growth, yield, and soil properties in wheat (Triticum aestivum L.) -maize (Zea mays L.) rotations: A four-year field experiment. Sci. Total Environ. 780, 146560. https://doi.org/10.1016/j.scitotenv.2021.146560.
101. El-sayed, M.E.A., Hazman, M., Abd El-Rady, A.G., Almas, L., McFarland, M., Shams El Din, A., Burian, S., 2021. Biochar Reduces the Adverse Effect of Saline Water on Soil Properties and Wheat Production Profitability. Agriculture. 11, 1112. https://doi.org/10.3390/agriculture11111112.
102. Dong, L., Wang, J., Shen, M., Zhang, H., Wang, L., Li, C., Lu, C., 2021. Biochar combined with nitrogen fertilizer affects soil properties and wheat yield in medium‐low‐yield farmland. Soil Use Manage. 38, 584–595. https://doi.org/10.1111/sum.12712.
103. Dawar, K., Saif ur, R., Fahad, S., Alam, S.S., Khan, S.A., Dawar, A., Younis, U., Danish, S., Datta, R., Dick, R.P., 2021. Influence of variable biochar concentration on yield-scaled nitrous oxide emissions, Wheat yield and nitrogen use efficiency. Sci. Rep. 11, 16774. https://doi.org/10.1038/s41598-021-96309-4.
104. Dawar, K., Fahad, S., Jahangir, M.M.R., Munir, I., Alam, S.S., Khan, S.A., Mian, I.A., Datta, R., Saud, S., Banout, J., Adnan, M., Ahmad, M.N., Khan, A., Dewil, R., Habib-ur-Rahman, M., Ansari, M.J., Danish, S., 2021. Biochar and urease inhibitor mitigate NH3 and N2O emissions and improve wheat yield in a urea fertilized alkaline soil. Sci. Rep. 11, 17413. https://doi.org/10.1038/s41598-021-96771-0.
105. Cong, X., Zhou, S., Wang, T., Pang, G., Xu, Z., 2021. Effects of Biochar Application and Irrigation on Soil Preferential Flow and Winter Wheat Productivity. Pol. J. Environ. Stud. 31, 625–635. https://doi.org/10.15244/pjoes/139103.
106. Brozović, B., Jug, I., Jug, D., Stipešević, B., Ravlić, M., Đurđević, B., 2021. Biochar and Fertilization Effects on Weed Incidence in Winter Wheat. Agronomy. 11, 2028. https://doi.org/10.3390/agronomy11102028.
107. Xiao, L., Yuan, G., Feng, L., Bi, D., Wei, J., 2020. Soil properties and the growth of wheat (Triticum aestivum L.) and maize (Zea mays L.) in response to reed (phragmites communis) biochar use in a salt-affected soil in the Yellow River Delta. Agric. Ecosyst. Environ. 303, 107124. https://doi.org/10.1016/j.agee.2020.107124.
108. Liu, H., Li. H., Zhang. A., Rahaman. M., Yang. Z., 2020. Inhibited effect of biochar application on N2O emissions is amount and time-dependent by regulating denitrification in a wheat-maize rotation system in North China. Sci. Total Environ. 721, 137636. https://doi.org/10.1016/j.scitotenv.2020.137636.
109. Liao, X., Niu, Y., Liu, D., Chen, Z., He, T., Luo, J., Lindsey, S., Ding, W., 2020. Four-year continuous residual effects of biochar application to a sandy loam soil on crop yield and N2O and NO emissions under maize-wheat rotation. Agric. Ecosyst. Environ. 302, 107109. https://doi.org/10.1016/j.agee.2020.107109.
110. Horák, J., Šimanský, V., Horák, J., Aydin, E., 2020. Benefits of biochar and its combination with nitrogen fertilization for soil quality and grain yields of barley, wheat and corn. Journal of Elementology. 25(2), 443–458. https://doi.org/10.5601/jelem.2019.24.3.1887.
111. Hafez, E.M., Kheir, A.M.S., Badawy, S.A., Rashwan, E., Farig, M., Osman, H.S., 2020. Differences in Physiological and Biochemical Attributes of Wheat in Response to Single and Combined Salicylic Acid and Biochar Subjected to Limited Water Irrigation in Saline Sodic Soil. Plants. 9, 1346. https://doi.org/10.3390/plants9101346.
112. Aydin, E., Šimanský, V., Horák, J., Igaz, D., 2020. Potential of Biochar to Alternate Soil Properties and Crop Yields 3 and 4 Years after the Application. Agronomy 10, 889. https://doi.org/10.3390/agronomy10060889.
113. Shahzad, K., Abid, M., Sintim, H.Y., Hussain, S., Nasim, W., 2019. Tillage and Biochar Effects on Wheat Productivity under Arid Conditions. Crop Sci. 59, 1191–1199. https://doi.org/10.2135/cropsci2018.08.0485.
114. Schillem, S., 2019. Effect of N-modified Lignite Granulates and Composted Biochar on Plant Growth, Nitrogen and Water Use Efficiency of Spring Wheat. Arch. Agron. Soil Sci. 158267. https://doi.org/ 10.1080/03650340.2019.1582767.
115. Liu, Y., Bi, Y., Xie, Y., Zhao, X., He, D., Wang, S., Wang, C., Guo, T., Xing, G., 2019. Successive straw biochar amendments reduce nitrous oxide emissions but do not improve the net ecosystem economic benefit in an alkaline sandy loam under a wheat–maize cropping system. Land Degrad. Dev. 31, 868–883. https://doi.org/10.1002/ldr.3495.
116. Ibrahim, M., Mahmoud, E., Gad, L., Khader, A., 2019. Effects of biochar and phosphorus fertilizer rates on soil physical properties and wheat yield on clay textured soil in middle Nile Delta of Egypt. Commun Soil Sci Plant Anal. 50, 2756–2766. https://doi.org/10.1080/00103624.2019.1679162.
117. Farahani, S.S., Asoodar, M.A., Moghadam, B.K., 2019. Short-term impacts of biochar, tillage practices, and irrigation systems on nitrate and phosphorus concentrations in subsurface drainage water. Environmental Sci Poll Res. 27, 761–771. https://doi.org/10.1007/s11356-019-06942-w.
118. Dong, X., Singh, B.P., Li, G., Lin, Q., Zhao, X., Aitkenhead, M., 2019. Biochar has little effect on soil dissolved organic carbon pool 5 years after biochar application under field condition. Soil Use Manage. 35, 466–477. https://doi.org/10.1111/sum.12474.
119. Dong, Y.B., Zheng, W., Zhang, X., Xiong, Z.Q., 2019. Dynamic responses of ammonia volatilization to different rates of fresh and field-aged biochar in a rice-wheat rotation system. Field Crops Res. 241, 107568. https://doi.org/10.1016/j.fcr.2019.107568.
120. Ali, K., Wang, X., Riaz, M., Islam, B., Khan, Z.H., Shah, F., Munsif, F., Haq, S.I.U., 2019. Biochar: an eco-friendly approach to improve wheat yield and associated soil properties on sustainable basis. Pak. J. Bot. 51. https://doi.org/10.30848/pjb2019-4(7).
121. Yeboah, S., Zhang, R., Cai, L., Jun, W., 2018. Different carbon sources enhance system productivity and reduce greenhouse gas intensity. Plant Soil Environ. 64, 463–469. https://doi.org/10.17221/83/2018-pse.
122. He, T., Liu, D., Yuan, J., Luo, J., Lindsey, S., Bolan, N., Ding, W., 2018. Effects of application of inhibitors and biochar to fertilizer on gaseous nitrogen emissions from an intensively managed wheat field. Sci. Total Environ. 628–629, 121–130. https://doi.org/10.1016/j.scitotenv.2018.02.048.
123. Zhang, A., Cheng, G., Hussain, Q., Zhang, M., Feng, H., Dyck, M., Sun, B., Zhao, Y., Chen, H., Chen, J., Wang, X., 2017. Contrasting effects of straw and straw–derived biochar application on net global warming potential in the Loess Plateau of China. Field Crops Res. 205, 45–54. https://doi.org/10.1016/j.fcr.2017.02.006.
124. Yeboah, S., Zhang, R., Cai, L., Li, L., Xie, J., Luo, Z., Wu, J., Antille, D.L., 2017. Soil water content and photosynthetic capacity of spring wheat as affected by soil application of nitrogen-enriched biochar in a semiarid environment. Photosynthetica. 55, 532–542. https://doi.org/10.1007/s11099-016-0672-1.
125. Sui, F., Zuo, J., Chen, D., Li, L., Pan, G., Crowley, D.E., 2017. Biochar effects on uptake of cadmium and lead by wheat in relation to annual precipitation: a 3-year field study. Environ. Sci. Pollut. Res. 25, 3368–3377. https://doi.org/10.1007/s11356-017-0652-4.
126. Niu, Y. H., Chen, Z.M., Muller, C., Zaman, M.M., Kim. D., Yu, H. Y., Ding, W. X., 2017. Yield-scaled N2O emissions were effectively reduced by biochar amendment of sandy loam soil under maize-wheat rotation in the North China Plain. Atmos. Environ. 170, 58–70. https://doi.org/10.1016/j.atmosenv.2017.09.050.
127. Kraska, P., Oleszczuk, P., Andruszczak, S., Kwiecińska-Poppe, E., Różyło, K., Pałys, E., Gierasimiuk, P., Michałojć, Z., 2016. Effect of various biochar rates on winter rye yield and the concentration of available nutrients in the soil. Plant Soil Environ. 62, 483–489. https://doi.org/10.17221/94/2016-pse.
128. Agegnehu, G., Nelson, P.N., Bird, M.I., 2016. The effects of biochar, compost and their mixture and nitrogen fertilizer on yield and nitrogen use efficiency of barley grown on a Nitisol in the highlands of Ethiopia. Sci. Total Environ. 569–570, 869–879. https://doi.org/10.1016/j.scitotenv.2016.05.033.
129. Xiang, J., Liu, D., Ding, W., Yuan, J., Lin, Y., 2015. Effects of biochar on nitrous oxide and nitric oxide emissions from paddy field during the wheat growth season. J. Clean. Prod. 104, 52–58. https://doi.org/10.1016/j.jclepro.2014.12.03.
130. Tammeorg, P., Simojoki, A., Mäkelä, P., Stoddard, F.L., Alakukku, L., Helenius, J., 2014. Short-term effects of biochar on soil properties and wheat yield formation with meat bone meal and inorganic fertiliser on a boreal loamy sand. Agric. Ecosyst. Environ. 191, 108–116. https://doi.org/10.1016/j.agee.2014.01.007.
131. Liu, X., Ye, Y., Liu, Y., Zhang, A., Zhang, X., Li, L., Pan, G., Kibue, G.W., Zheng, J., Zheng, J., 2014. Sustainable biochar effects for low carbon crop production: A 5-crop season field experiment on a low fertility soil from Central China. Agricultural Systems. 129, 22–29. https://doi.org/10.1016/j.agsy.2014.05.008.
132. Zhang, Q. Z., Wang, X. H., Du, Z.L., Liu, X. R., Wang, Y. D., 2013. Impact of biochar on nitrate accumulation in an alkaline soil. Soil Res. 51, 521–528. https://doi.org/10.1071/sr13153.
133. Zhang, A., Bian, R., Hussain, Q., Li, L., Pan, G., Zheng, J., Zhang, X., Zheng, J., 2013. Change in net global warming potential of a rice–wheat cropping system with biochar soil amendment in a rice paddy from China. Agric. Ecosyst Environ. 173, 37–45. https://doi.org/10.1016/j.agee.2013.04.001.
134. Tammeorg, P., Simojoki, A., Mäkelä, P., Stoddard, F.L., Alakukku, L., Helenius, J., 2013. Biochar application to a fertile sandy clay loam in boreal conditions: effects on soil properties and yield formation of wheat, turnip rape and faba bean. Plant Soil. 374, 89–107. https://doi.org/10.1007/s11104-013-1851-5.
135. Karer, J., Wimmer, B., Zehetner, F., Kloss, S., Soja, G., 2013. Biochar application to temperate soils: effects on nutrient uptake and crop yield under field conditions. Agric. Food Sci. 22, 390–403. https://doi.org/10.23986/afsci.8155.
136. Vaccari, F.P., Baronti, S., Lugato, E., Genesio, L., Castaldi, S., Fornasier, F., Miglietta, F., 2011. Biochar as a strategy to sequester carbon and increase yield in durum wheat. Eur. J. Agron. 34, 231–238. https://doi.org/10.1016/j.eja.2011.01.006.
137. Solaiman, Z.M., Blackwell, P., Abbott, L.K., Storer, P., 2010. Direct and residual effect of biochar application on mycorrhizal root colonisation, growth and nutrition of wheat. Soil Res. 48, 546–554. https://doi.org/10.1071/sr10002.
138. Blackwell, P., Krull, E., Bulter, G., Herbert, A., Solaiman, Z., 2010. Effect of banded biochar on dryland wheat production and fertiliser use in south-western Australia: an agronomic and economic perspective. Aust. J. Soil Res. 48, 531–545. https://doi.org/[10.1071/SR10014](http://dx.doi.org/10.1071/SR10014).
